# Milieu-specific differences in symptom severity and treatment outcome in psychosomatic rehabilitation in Germany

**DOI:** 10.3389/fpsyt.2023.1198146

**Published:** 2023-08-15

**Authors:** Henrika Kleineberg-Massuthe, Lilia Papst, Markus Bassler, Volker Köllner

**Affiliations:** ^1^Psychosomatic Rehabilitation Research Group, Department of Psychosomatic Medicine, Centrum für Innere Medizin und Dermatologie, Charité – Universitätsmedizin Berlin, Berlin, Germany; ^2^Institute for Social Medicine, Rehabilitation Sciences and Health Services Research (ISRV), Hochschule Nordhausen, Nordhausen, Germany; ^3^Center for Oncology and Psychosomatics, Rehazentrum Oberharz, Clausthal-Zellerfeld, Germany; ^4^Department of Psychosomatics and Behavioral Psychotherapy, Reha-Zentrum Seehof, Teltow, Germany

**Keywords:** psychosomatic medicine, rehabilitation, treatment outcome, social milieu, mental health disparities, social determinants of health, social inequality, health equity

## Abstract

**Introduction:**

Previous studies that focused on socioeconomic differences did not comprehensively explain existing inequalities in psychosomatic rehabilitation in Germany. We applied a social milieu approach, which additionally includes sociocultural factors such as lifestyles, attitudes and values, to investigate differences among patients in symptom severity, psychosocial impairment and improvement over the course of the intervention.

**Methods:**

As a model for social milieus, the empirical *Sinus milieus* were used. 2,000 patients of two psychosomatic rehabilitation clinics in Germany were included and their milieu was assessed with the *Sinus milieu indicator for Germany 10/2018* questionnaire. BDI-II (*N* = 1,832) and HEALTH-49 (*N* = 1,829) questionnaires were used to measure depressiveness and psychosocial impairment at admission (T0) and discharge after 5 weeks of treatment (T1). Milieu differences in severity and improvement were analyzed by mixed-model ANOVAs.

**Results:**

Milieu distribution was not representative of the overall population of Germany. We found significant differences between patients from different milieus in both BDI-II and HEALTH-49 (*p* < 0.001). Patients from the Precarious Milieu had the highest burden of depressive symptoms in BDI-II and the highest impairment on all HEALTH-49 scales at T0 and T1. Over the course of rehabilitation, patients from all milieus improved significantly in all domains (*p* < 0.001). Significant interaction effects showed milieu-dependent differences in improvement for depressiveness on the BDI-II [*F*(9, 1822) = 2.50, *p* = 0.008] and for three HEALTH-49 scales, namely Psychological well-being [*F*(9, 1819) = 3.30, *p*_adj_ = 0.005], Interactional difficulties [*F*(9, 1819) = 2.74, *p*_adj_ = 0.036] and Activity and Participation [*F*(9, 1819) = 4.94, *p*_adj_ < 0.001], while post-hoc tests only revealed two significant group differences for the last scale. In all domains, patients from the Precarious Milieu retained higher symptoms and impairment at T1 than patients from better-off milieus had at T0.

**Discussion:**

Social milieu was associated with symptom severity, treatment access and outcome of psychosomatic rehabilitation patients. Milieu-specific sociocultural habits, psychosocial needs and therapeutic demands may help describe differences and should be considered in therapy planning and implementation, to improve equal access, quality and effectiveness of rehabilitation. Therefore, further research on milieu-specific differences and needs is necessary.

## 1. Introduction

Health inequalities are systematic, avoidable differences in health outcomes between social groups (1) and may deprive people of life chances based on their position in society (2). Mental health inequalities, particularly those related to the negative impacts of socioeconomic disadvantage, have been widely documented: for instance, disadvantaged socioeconomic status was found to be related to higher prevalences of negative life events, chronic stress (3), mental health problems (4), and mental disorders (4, 5), such as depression (6). Living in deprived areas (e.g., with low neighborhood income) and low formal education were associated with higher rates of mood disorders (7, 8), psychotic disorders, self-harm, substance abuse, and dementia (8). Neighborhood deprivation was additionally related to higher prevalences of anxiety disorders and poor mental health in general (7). Low education was furthermore associated with higher rates of neurotic disorders (8) and suicide (9). Moreover, disadvantaged people assessed their own health (3, 4) and health-related quality of life more negatively (4).

Socioeconomic disadvantage may also affect the treatment of mental illness, for instance in psychosomatic rehabilitation. In Germany, psychosomatic rehabilitation is an important sector of mental health care, aiming at preventing, treating and compensating for (chronic) mental disorders. Rehabilitation is carried out in specialized clinics throughout the country. It is indicated when patients are impaired by mental illness to such an extent or for such a length of time so that their social or occupational participation is restricted or endangered (10). The overall effectiveness and treatment success of psychosomatic rehabilitation, in terms of symptom improvement, benefit assessment and work and earning capacity, have been demonstrated in many studies (11–13). Previous research has also shown that in psychosomatic rehabilitation, both symptom severity and treatment outcome differ in relation to socioeconomic factors. For instance, higher income, formal education and vocational status were associated with better subjective health at the beginning of the rehabilitation (14). Higher income was also correlated with stronger improvement in depressiveness (15) and higher vocational status was related to stronger improvement in subjective health after rehabilitation (14). While there was no relevant association between education and the improvement of well-being as well as work ability in one study (16), others found that lower formal education was negatively associated with the improvement of psychological stress, depressiveness (17) and subjective health (14). When a stratification index (income, formal education, and vocational status) was applied, the so-called lower class was the most impaired group with regard to almost all of the examined aspects of subjective health at the beginning of rehabilitation; these patients were not able to compensate for the initial differences compared with the so-called upper class (18).

While these studies showed an association between socioeconomic factors and the health status and treatment outcome of patients, they do not give sufficient evidence to comprehensively describe the dimensions and driving factors of inequalities in psychosomatic rehabilitation in Germany. Although there is no generally valid, theoretically sound and empirically proven explanatory model for the phenomenon of health inequality (19), there is agreement on the complexity of its causes (20). Explanatory approaches such as the consideration of socioeconomic factors can thus only partially contribute to the description of health inequalities (20). Comparable to the use of socioeconomic status, class and stratification approaches, milieu approaches have long been used in applied social science to structure large social groups (21). These approaches have the advantage of including other socially structuring factors in addition to socioeconomic ones, as people with similar economic backgrounds may still differ in sociocultural aspects, such as lifestyles, basic values and attitudes (22–24), which could affect both access to and needs regarding health care. In this way, milieu models attempt to represent social groups that better reflect everyday life than the sole categorization, e.g., into different social strata, would be able to do. The social milieus, analogous to different strata in a stratification model, each have different social privileges or disadvantages and thus also different health opportunities (25). Initial studies have shown different health outcomes for people from different social milieus and that these theoretical approaches can help to further describe the phenomenon of health inequality (25, 26).

To complement the existing body of research on socioeconomic differences in symptom severity and treatment outcome in psychosomatic rehabilitation in Germany, we conducted a study applying a milieu approach as it is introduced above. In order to use our available resources most efficiently and to obtain a high level of standardization for the survey, an existing milieu model was used: the *Sinus milieus* (in German: Sinus-Milieus) represent the first and so far only applied empirical milieu model in German-speaking countries (24). The model is theory-based, quantitatively post-modeled and validated and incorporates socioeconomic as well as sociocultural factors (24, 27). It can be used for surveys that aim to quantify different social milieus and determine differences between them. The available extensive characterization of the Sinus milieus offers approaches for theoretical considerations on the causes of observed statistical differences between people from different milieus. The application of the model could thus help to generate hypotheses about further relationships between social structuring and health outcomes, as well as mediating mechanisms (cf. chapter 2.2.).

This is of particular interest and relevance for the care setting of psychosomatic rehabilitation. The statutory pension insurance in Germany, as one of the main providers, has the task and responsibility to offer suitable and effective services to insured persons of different social backgrounds. Following a biopsychosocial model of disease, the therapies in psychosomatic rehabilitation already take into account social aspects of the development and maintenance of mental illness as well as social stresses that affect the patients. For example, stress in the workplace is generally addressed, since most patients are currently unable to work or are at risk of reduced earning capacity. However, it depends on the particular therapists to what extent and in what way they address the individual socioeconomic and sociocultural stresses and resources of the patients. So far, these aspects are neither recorded nor considered in a systematic and standardized way. Thus, current treatment plans, assignment to therapy groups and specific therapy content of patients are not yet systematically oriented to socially unequally distributed factors (cf. chapter 2.1.).

Against this background, we first examined which social milieus are represented in psychosomatic rehabilitation and how the milieu distribution of the study sample relates to that of a representative sample of the total population in Germany. Our aims were to investigate the association between social milieu and the severity of psychological symptoms, psychosocial impairments and symptom improvement over the course of rehabilitation. With our results we want to provide empirical evidence to the discussion whether psychosomatic rehabilitation is sufficiently adapted to milieu-specific differences and demands.

## 2. Materials and methods

### 2.1. Sample and intervention

Our survey was conducted between March 2019 and March 2020 in the psychosomatic departments of two rehabilitation clinics in Germany: the Seehof clinic near Berlin, run by the *Federal German Pension Agency*, and the Oberharz clinic in Lower Saxony, run by the regional *Pension Agency Braunschweig-Hannover*. Both clinics are specialized in the treatment of mood (affective), neurotic, stress-related and somatoform disorders. All adult patients who matched the criteria were consecutively included until the predefined sample size of *N* = 2,000 (*N* = 1,000 for each of the clinics) had been reached. The targeted sample size was chosen for, inter alia, economic and practical reasons. Notably, we estimated that a sample size of *N* = 1,000 could be achieved within the intended study duration of 1 year, given the usual admission rate in both clinics. Moreover, as the milieu shares in the study sample were unknown prior to the study, we assumed that this sample size would ensure sufficient group sizes for all milieus in order to enable a meaningful statistical analysis. The following inclusion criteria were applied: present indication for psychosomatic rehabilitation (taken as given on admission to the clinic), full participation in the five-week intervention (cf. next paragraph), adequate German language comprehension (assessed by medical staff), absence of severe cognitive impairment and medical emergencies including acute psychological crises (assessed by medical staff) as well as a completed milieu assignment (cf. chapter 2.3.).

Rehabilitation treatment in the two clinics has a regular duration of 5 weeks and addresses biological, psychological and social factors (although the latter are not considered in a systematic and standardized way; cf. chapter 1). The treatment aims at reducing symptoms, training capacities, helping to cope with chronic impairments and restoring well-being, everyday life and working abilities. Therefore, a multimodal and interdisciplinary treatment approach is applied (10): components of the 20 to 25 h per week program are psychotherapy, sports therapy, physiotherapy, occupational therapy, relaxation methods, creative therapy, socio-medical counseling as well as health, nutrition and psychoeducation. In addition, pharmacological treatment is provided when necessary. Psychotherapy takes place in individual and group sessions. Patients are assigned to two different psychotherapy groups, one on the basis of the time of admission (patients admitted on the same day are assigned to one group) and the second on the basis of individual diagnoses and personal impairments, i.e., there are groups for patients with anxiety, physical problems, and depression as well as life and workplace problems, among others. Most other therapies are also conducted in groups. The selection of specific further therapies, e.g., the type of sports therapy and relaxation methods, is made jointly by the treatment team and the patients, after which the patients are assigned to the respective groups.

### 2.2. Sinus milieus as an empirical milieu model

The Sinus milieus applied in this study represent an empirical model developed by the privately run *Sinus institute* in Germany (24). It is based on a large number of qualitative interviews and home visits to the country’s population. With a validated questionnaire called the *Sinus milieu indicator* (in German: Sinus-Milieuindikator), milieus can be assigned to the respondents. The milieus and questionnaire are continuously re-evaluated and adapted to changing social realities (24, 27). In our study the model of 2020 was applied; the latest version of 2021 was released after the study had been completed.

The Sinus model aims to describe people’s “lifeworld” in terms of socioeconomic and sociocultural factors and has the ambition to portray social realities as realistically as possible. To this end, social situations, basic value orientations, lifestyles, everyday attitudes, life strategies, aspirations, fears and future expectations were investigated in a large sample of the population in Germany. The empirical findings of the interviews were condensed into a basic typology, the Sinus milieus. The model groups people on the basis of similarities concerning their social situation, lifestyle and outlook on life, which is how different social milieus can be distinguished from one another and described, with each of them having characteristic features (27). In this way, the model depicts various aspects of social realities in a standardized way. The application thus allows for a complex combined socioeconomic and sociocultural clustering without the necessity and needed resources to collect a large number of individual factors.

**Figure 1:**
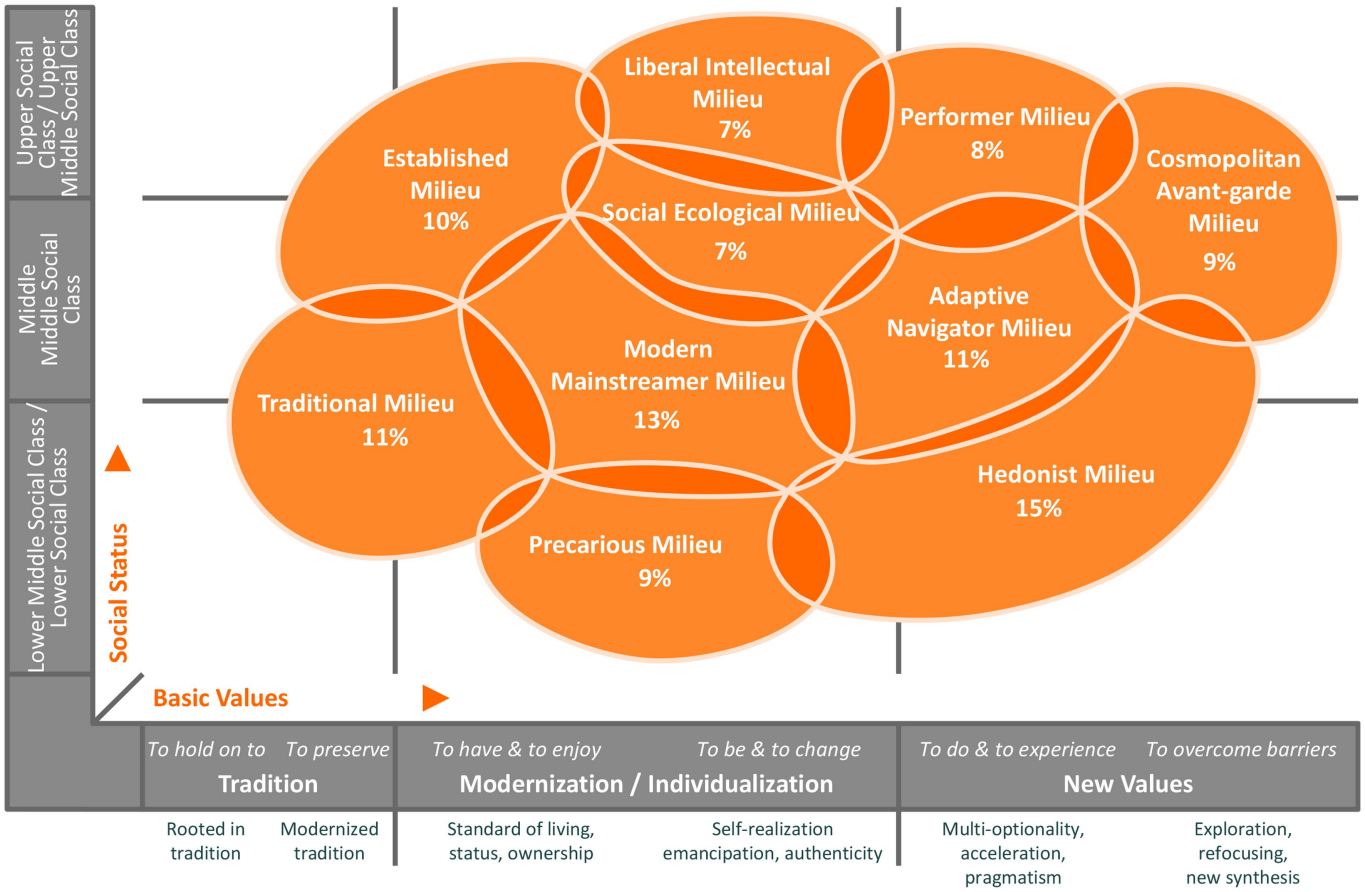
Sinus milieus in Germany in the 2020 model and their percentage share of the total population, *N* = 30,178 (© SINUS 2020).

In the model of 2020, ten Sinus milieus were defined for the general population in Germany. They can be visualized in a milieu diagram (cf. Figure 1), applying the dimensions “Social Status” (on the vertical axis) and “Basic Values” (on the horizontal axis). In the diagram, each of the two axes is divided into three sections. The dimension Social Status represents factors of the social situation, such as income, formal education level and occupational status (socioeconomic factors). From the bottom to the top, the axis is divided into the categories lower social class/lower middle social class, middle middle social class and upper middle social class/upper social class. The dimension Basic Values represents factors such as lifestyle, orientations, and lifegoals (sociocultural factors). From left to right, there is a classification into the categories tradition, modernization/individualization and new values. This reading direction also refers to the dynamic development of the predominant basic values in society over time (27), as people of higher age tend to be situated in milieus on the left and people of younger age tend to be situated in milieus on the right side of the diagram (28). The position of the Sinus milieus in the diagram is generally not restricted by the imaginary boundaries of the Social Status and Basic Values categories; indeed, most of the milieus reach across different social classes and value orientations. Theoretically, the milieus overlap with others at their edges. Nevertheless, the practical application of the model allows a clear assignment of the best-fitting Sinus milieu to each participant. The milieu designations have emerged from the sociological research tradition and do not comprehensively characterize the respective milieu. They are rather of illustrative character (27).

In the following, we provide a brief description of the 2020 model’s milieus as they are characterized by the Sinus institute (27): the “Established Milieu” is considered the classical establishment with status awareness, an ethic of responsibility and success, a claim to exclusivity and leadership and an increasing desire for order and balance. The “Liberal Intellectual Milieu” represents an informed and educated elite with a critical world view, a liberal attitude, post-material roots and desire for self-determination and self-expression. The “Performer Milieu”, a multi-optional, efficiency-oriented performance elite, is characterized by global economic thinking, a self-image as consumer and style avant-garde and a high affinity for technology and IT. The “Cosmopolitan Avant-garde Milieu” is regarded as the ambitious creative avant-garde, geographically, culturally and mentally mobile and networking, in search of new horizons and solutions and with an appearance as trendsetters. The “Modern Mainstreamer Milieu”, the middle class mainstream that is willing and ready to perform and adapt, affirms the social order, is described as having a desire for secure circumstances as well as for professional and social establishment and at the same time showing growing excessive demands and fears of social decline. The “Social Ecological Milieu” is an engaged and socially critical milieu with normative ideas of the right life, a strong ecological and social awareness, scepticism about globalisation and commitment to political correctness and diversity. The “Adaptive Navigator Milieu” is considered as modern, young and situated in the middle class with strong pragmatism and orientation towards usefulness; it is ambitious, flexible and cosmopolitan and shows a need for roots, belonging and entertainment. The “Traditional Milieu” is depicted as the security- and order-loving older generation, maintaining the petit-bourgeois world or traditional working-class culture, characterized by thriftiness, adaptation to necessities, increasing resignation and feelings of being left behind. The “Precarious Milieu” is regarded as people from the lower class striving for orientation and participation, with a desire to catch up with consumption standards of the broad middle class, with resentments and the experience of exclusion. Finally, the “Hedonist Milieu” represents fun- and adventure-oriented modern lower class or lower middle class people, who are described as spontaneous, often adapted at work and breaking out of everyday pressures in their free time (27).

### 2.3. Instruments and statistics

**Figure 2:**
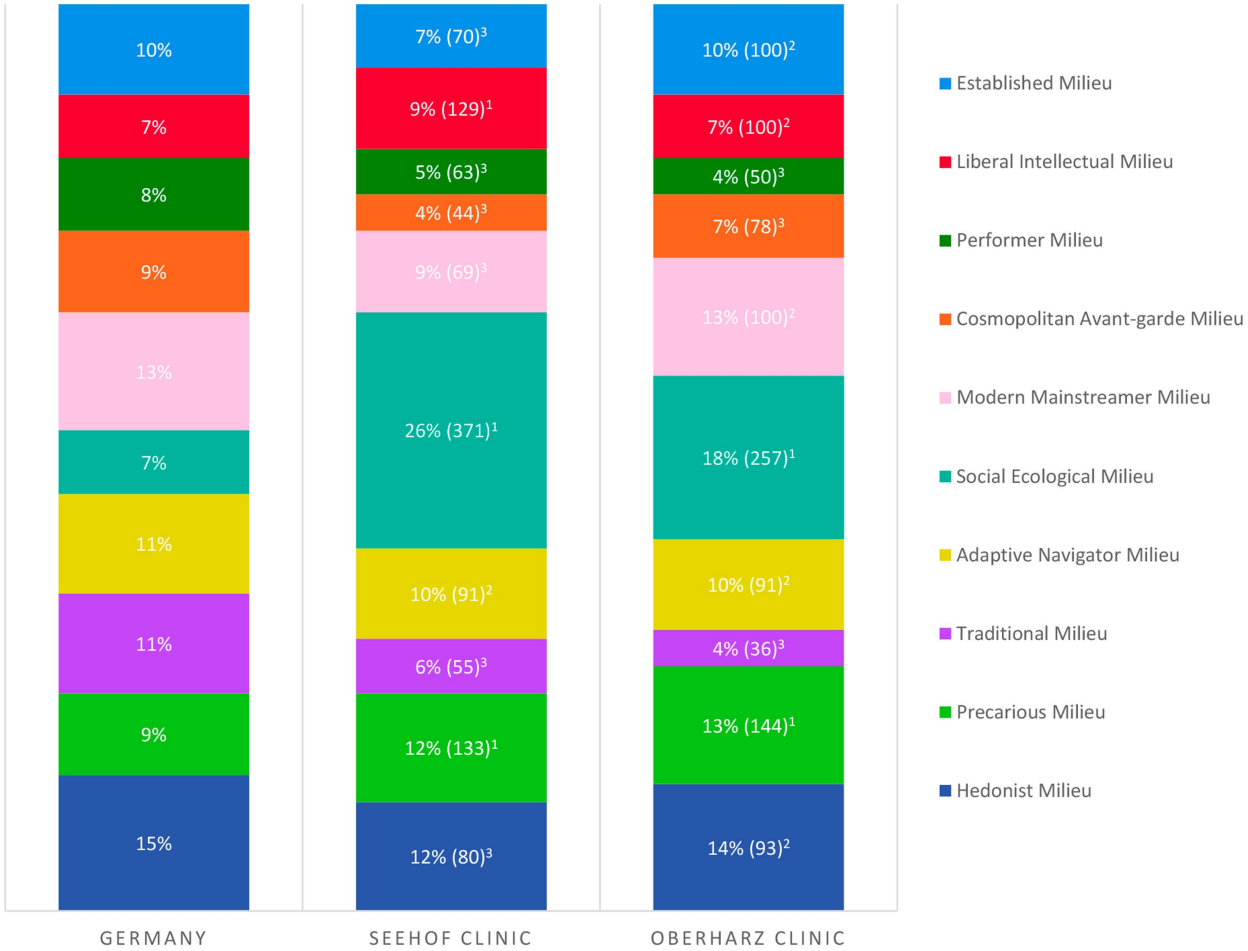
Milieu distribution in the rehabilitation clinics Seehof and Oberharz (*N* = 1,000 each) compared to a representative sample of the total population of Germany (cf. Figure 1). Number in percent: milieu share in the study sample; number in brackets: milieu share in the study sample ÷ milieu share in total population × 100; classification of representativeness: ^1^milieu overrepresented (≥ 120), ^2^representative (< 120, > 80), and ^3^underrepresented (≤ 80).

Medical staff of the rehabilitation clinics routinely documented the sociodemographic characteristics of the patients and their diagnoses of mental and behavioral disorders (F-diagnoses), according to the International Statistical Classification of Diseases and Related Health Problems, 10th Revision, German Modification (ICD-10-GM; 29). The *Sinus milieu indicator for Germany 10/2018* questionnaire was answered by the patients at T0 (clinic admission). The assignment of specific Sinus milieus was then performed by the Sinus institute using cluster analysis. For both clinic samples, the number of patients belonging to the respective milieus was counted. Based on the sample size of *N* = 1,000 per clinic, the percentage of the ten milieus in each sample was then determined. A rule of three was used to compare the milieu distribution of the two clinic samples with a representative sample of the total population of Germany. By doing so, the percentage share of a milieu in the clinic sample (e.g., percentage share of the Established Milieu in the Seehof clinic was 7%) was put in relation to the percentage share of the corresponding milieu in the representative reference sample (e.g., Established Milieu in reference sample was 10%). The reference milieu was set equal to 100 (e.g., 7% ÷ 10% × 100 = 70; cf. Figure 2). Classification of representativeness was derived from defined threshold values by the Sinus institute. A milieu in the clinic sample was considered as overrepresented compared to the corresponding milieu in the total population of Germany if a value ≥ 120 was calculated in the rule of three, as representative if a value < 120 and > 80 was calculated and as underrepresented if a value ≤ 80 was the case (e.g., with a value of 70, the Established Milieu in the Seehof clinic was underrepresented). To assess symptom severity and outcome, patients completed two questionnaires at T0 and T1 (clinic discharge): the *German version of the revised Beck Depression Inventory* (BDI-II; 30) and the *Hamburg Modules for the Assessment of Psychosocial Health in Clinical Practice* (HEALTH-49; 31).

The BDI-II assesses the presence and severity of depressive symptoms according to the diagnostic criteria of the Diagnostic and Statistical Manual of Mental Disorders, 4th Edition (DSM-IV; 30, 32). Patients are asked to evaluate the severity of their symptoms (e.g., sadness, loss of interest, changes in sleeping habits) over the last 2 weeks by rating 21 items on four-point Likert scales (0–3 points per item). The points are then added up to a total sum score, with higher scores indicating higher symptom severity, ranging from not present (0–8 points) over minimal (9–13 points), mild (14–19 points), and moderate (20–28 points) to severe (29–63 points). The German version of the BDI-II has been psychometrically tested and meets all relevant test quality criteria. The correlations with the construct-related, self-assessed German short form of the Inventory to Diagnose Depression (in German: Fragebogen zur Depressionsdiagnostik nach DSM-IV, FDD-DSM-IV) ranged from *r* = 0.72 for a sample of depressive patients to *r* = 0.81 for a healthy sample. The reliability (internal consistency; Cronbach’s alpha) was α = 0.93 in a sample of depressive patients in treatment, α = 0.92 in patients with primarily other mental disorders and α = 0.90 in a healthy population. Objectivity of execution, evaluation and interpretation are given with the computer-based application of the normed test (33).

The HEALTH-49 questionnaire consists of 49 items to be rated on five-point Likert scales by the patients (0–4 points per item). Ten scale scores are calculated from the means of multiple corresponding items, with higher scores expressing higher impairments in the specific areas: (1) Somatoform complaints (e.g., pain in muscles or joints), (2) Depressiveness (e.g., feeling of hopelessness), (3) Phobic anxiety (e.g., fear of leaving the house alone), (4) Psychological and somatoform complaints (sum of the previous three scales), (5) Psychological well-being (e.g., feeling of relaxation), (6) Interactional difficulties (e.g., difficulty in raising important concerns with others), (7) Self-efficacy (e.g., ability to achieve personal goals), (8) Activity and Participation (e.g., impairments in occupation, household and free time), (9) Social support (e.g., support from someone when it is needed), and (10) Social stress (e.g., problems are talked down by close persons). The reliability (internal consistency; Cronbach’s alpha) of the HEALTH-49 scales can be rated as predominantly high with α between 0.76 and 0.91 in a sample of patients in primary care and between 0.73 and 0.90 in rehabilitation patients with mental illness, respectively. Construct validity was demonstrated by correlations of *r* > 0.80 between the scales Somatoform complaints, Depressiveness, Phobic anxiety as well as Psychological and somatoform complaints and the respective corresponding scales of a short version of the Symptom Checklist 90-Revised (SCL-14) in a rehabilitation sample. For the Somatoform complaints scale, there was also a high correlation with the physical sum scale of the Short-Form Health Survey (SF-8; *r* = 0.71). The Interactional difficulties scale correlated particularly highly with the content-related total score on the German short version of an inventory for the assessment of interpersonal problems (in German: Inventar zur Erfassung interpersonaler Probleme, IIP-25; *r* = 0.75). All HEALTH-49 scales proved to be suitable for differentiating between healthy and mentally ill persons, with healthy persons having significantly lower impairment scores in each case and differences reaching the magnitude of large effect sizes (Cohen’s *d* > 0.80) except for the Social support scale (*d* = 0.34). With regard to sensitivity to change during inpatient psychosomatic or psychotherapeutic rehabilitation, seven of the scales showed changes to the extent of at least a medium effect size; changes on the Phobic anxiety scale reached the extent of a small effect size and on the Social support and Social stress scales only less than small change effects were found (34).

The statistical analyses were conducted with IBM SPSS Statistics 28. For this purpose, 168 (BDI-II) resp. 171 (HEALTH-49) patients had to be excluded from the total sample of *N* = 2,000 because of missing values or nonresponse to the questionnaires. The test requirements were examined in advance and a significance level of α ≤ 0.05 was assumed. Mixed-model ANOVAs (split-plot ANOVAs) were performed for the BDI-II (*N* = 1,832) and separately for each of the ten scales of the HEALTH-49 (*N* = 1,829), applying the Bonferroni correction for the latter and determining adjusted values of *p* (*p*_adj_). Interaction effects (time × milieu) and main effects (time, milieu) were analyzed. No covariates were included in the statistical procedure because there were no assumptions about factors affecting the dependent variables that were not already part of the milieu model. To assess effect sizes, partial eta-squared (η^2^_part_) was used (35), with η^2^_part_ ≥ 0.01 describing a weak, η^2^_part_ ≥ 0.06 a medium, and η^2^_part_ ≥ 0.14 a strong effect (36). To examine group differences (main effect of milieu), Tukey post-hoc tests (in case of variance homogeneity) or Games-Howell post-hoc tests (in case of variance inhomogeneity) were performed for the BDI-II and the ten scales of the HEALTH-49. For significant interaction effects, differences in improvement between patients from different milieus were examined by single-factor ANOVAs with subsequent post-hoc tests on pre-post differential values (variance homogeneity: Tukey test, variance inhomogeneity: Games-Howell test). In all post-hoc tests, the significance level was adjusted for 45 group comparisons in each case (each milieu was compared with each other) with α_adj_ ≤ 0.001 after the Bonferroni correction.

## 3. Results

### 3.1. Sample characteristics and milieu distribution

Sociodemographic characteristics of the patient collectives of the two clinics as well as the prevalence of F-diagnoses among the patients are depicted in Table 1. All Sinus milieus were represented in the samples, but the overall milieu distribution was not representative of the general population of Germany. Milieu proportions also differed between the clinics (cf. Figure 2). In both clinics, the Social Ecological Milieu and the Precarious Milieu were overrepresented and the Performer Milieu, the Cosmopolitan Avant-garde Milieu and the Traditional Milieu were underrepresented compared to the representative sample of the total population of Germany. The Social Ecological Milieu was by far the largest milieu in both samples.

**Table 1:** Sociodemographic characteristics and F-diagnoses among the patients of the two clinic samples.

	Seehof *N* = 1,000	Oberharz *N* = 1,000	Total *N* = 2,000
Age			
M in years (SD)	52.02 (8.99)	50.22 (9.18)	51.12 (9.13)
	*N* (%)		
Sex			
Female	660 (66.0)	473 (47.3)	1,133 (56.7)
Male	340 (34.0)	527 (52.7)	867 (43.4)
Graduation^a^			
Special school graduation, in education, without graduation	13 (1.3)	24 (2.4)	37 (1.9)
Basic secondary school graduation	90 (9.0)	293 (29.3)	383 (19.2)
Junior high school graduation	437 (43.7)	394 (39.4)	831 (41.6)
High school graduation	287 (28.7)	215 (21.5)	502 (25.1)
Other graduation	1 (0.1)	0 (0)	1 (0.05)
Missing	172 (17.2)	74 (7.4)	246 (12.3)
Professional Qualification			
Without professional qualification	32 (3.2)	115 (11.5)	147 (7.4)
In training	3 (0.3)	9 (0.9)	12 (0.6)
Apprenticeship, technical school, master school	543 (54.3)	636 (63.6)	1,179 (59.0)
University of applied sciences, university	215 (21.5)	86 (8.6)	301 (15.1)
Other degree	35 (3.5)	80 (8.0)	115 (5.8)
Missing	172 (17.2)	74 (7.4)	246 (12.3)
Mental and behavioral disorders according to ICD-10-GM^b^			
F00–F09: Organic, including symptomatic, mental disorders	9	2	11
F10–F19: Mental and behavioral disorders due to psychoactive substance use	111	158	269
F20–F29: Schizophrenia, schizotypal and delusional disorders	3	5	8
F30–F39: Mood (affective) disorders	752	447	1,199
F40–F48: Neurotic, stress-related and somatoform disorders	873	782	1,655
F50–F59: Behavioral syndromes associated with physiological disturbances and physical factors	158	21	179
F60–F69: Disorders of adult personality and behavior	84	27	111
F70–F79: Mental retardation	1	0	1
F80–F89: Disorders of psychological development	24	0	24
F90–F98: Behavioral and emotional disorders with onset usually occurring in childhood and adolescence	12	6	18
Average number of diagnoses per patient	2.03	1.45	1.74

M, mean; SD, standard deviation.

a All categories including equivalent and comparable graduation.

b The number of diagnoses assigned to a category is given here; it does not necessarily reflect the number of patients, since a patient can also have several diagnoses from one category.

### 3.2. Symptom severity and treatment outcome

All statements below about patients from a particular milieu refer to the mean of all patients from that milieu.

#### 3.2.1. Depressiveness (BDI-II)

At T0, patients from the Precarious Milieu (Mean 32.76) and the Traditional Milieu (Mean 28.69) had the highest scores, indicating severe depressive symptoms. Patients from the Liberal Intellectual Milieu (Mean 18.40) and the Established Milieu (Mean 18.92) had the lowest scores, corresponding to mild depressive symptoms. Patients from all other milieus had moderate symptoms at admission. At T1, patients from the Liberal Intellectual Milieu (Mean 7.36) and the Established Milieu (Mean 8.07) were in the symptoms “not present” range according to the BDI-II. Patients from the Precarious Milieu still had the highest score (Mean 23.37), indicating moderate symptom severity, with a higher burden remaining at discharge than patients from more socioeconomically privileged milieus, according to the Sinus model, had at the beginning of the treatment. Patients from the other milieus had minimal or mild symptoms after the intervention (cf. Table 2).

**Table 2:** Results of BDI-II and HEALTH-49: Scores at admission (T0) and discharge after five-week rehabilitation treatment (T1), pre-post differences and test statistics for mixed-model ANOVAs.

	EST	LIB	PER	COS	MOD	SOC	ADA	TRA	PRE	HED	df	*F*	*p*	*p*_adj_	η^2^_part_
BDI-II^a^, *N* = 1,832 (91.6)^b^															
*N* (%)^c^	144 (86.7)	157 (93.5)	76 (91.6)	97 (89.8)	196 (90.3)	412 (94.1)	182 (91.5)	98 (93.3)	229 (90.5)	241 (91.6)					
Depressiveness															
T0 M (SD)	18.92 (11.48)	18.40 (10.12)	22.17 (11.60)	20.08 (10.02)	23.07 (11.36)	23.69 (10.62)	25.79 (12.01)	28.69 (13.13)	32.76 (11.12)	27.57 (11.97)					
T1 M (SD)	8.07 (9.01)	7.36 (9.36)	11.83 (11.77)	11.75 (11.74)	14.35 (12.95)	12.36 (11.06)	16.37 (13.07)	16.62 (13.90)	23.37 (14.03)	16.61 (13.21)					
MD (SD)	−10.85 (8.22)	−11.04 (9.63)	−10.34 (7.69)	−8.33 (9.79)	−8.72 (10.09)	−11.33 (9.62)	−9.42 (10.44)	−12.07 (10.82)	−9.39 (10.17)	−10.97 (10.28)					
Time × Milieu											9, 1822	2.50	**0.008**		0.01
Time											1, 1822	1589.17	**<0.001**		0.47
Milieu											9, 1822	34.06	**<0.001**		0.14
HEALTH-49^a^, *N* = 1,829 (91.5)^b^															
*N* (%)^c^	144 (86.7)	157 (93.5)	76 (91.6)	97 (89.8)	195 (89.9)	410 (93.6)	182 (91.5)	98 (93.3)	229 (90.5)	241 (91.6)					
(1) Somatoform complaints															
T0 M (SD)	1.28 (0.89)	1.33 (0.93)	1.55 (0.85)	1.55 (0.81)	1.70 (0.95)	1.71 (0.88)	1.80 (0.92)	1.79 (1.02)	2.25 (0.96)	1.81 (0.93)					
T1 M (SD)	0.81 (0.78)	0.84 (0.79)	0.99 (0.87)	1.16 (0.90)	1.29 (0.99)	1.24 (0.89)	1.41 (0.93)	1.36 (0.98)	1.86 (0.98)	1.32 (0.92)					
MD (SD)	−0.47 (0.69)	−0.48 (0.80)	−0.56 (0.69)	−0.39 (0.75)	−0.40 (0.77)	−0.48 (0.72)	−0.39 (0.72)	−0.43 (0.70)	−0.38 (0.70)	−0.49 (0.87)					
Time × Milieu											9, 1819	0.86	0.562	1	
Time											1, 1819	518.54	**<0.001**	**<0.001**	0.22
Milieu											9, 1819	22.15	**<0.001**	**<0.001**	0.10
(2) Depressiveness															
T0 M (SD)	1.30 (0.93)	1.22 (0.91)	1.56 (1.01)	1.46 (0.92)	1.73 (0.98)	1.63 (0.96)	1.86 (0.99)	2.00 (0.99)	2.46 (0.90)	1.97 (0.95)					
T1 M (SD)	0.66 (0.78)	0.52 (0.71)	0.90 (0.97)	0.91 (0.94)	1.12 (1.04)	0.92 (0.87)	1.20 (0.99)	1.31 (1.01)	1.87 (1.08)	1.32 (1.04)					
MD (SD)	−0.64 (0.69)	−0.70 (0.80)	−0.66 (0.67)	−0.54 (0.78)	−0.60 (0.80)	−0.71 (0.82)	−0.67 (0.86)	−0.69 (0.85)	−0.58 (0.85)	−0.66 (0.84)					
Time × Milieu											9, 1819	0.79	0.625	1	
Time											1, 1819	927.28	**<0.001**	**<0.001**	0.34
Milieu											9, 1819	35.76	**<0.001**	**<0.001**	0.15
(3) Phobic anxiety															
T0 M (SD)	0.60 (0.84)	0.58 (0.86)	0.79 (0.96)	0.53 (0.78)	1.03 (1.03)	0.87 (1.00)	1.04 (1.06)	1.31 (1.08)	1.69 (1.14)	1.08 (1.03)					
T1 M (SD)	0.34 (0.65)	0.33 (0.68)	0.44 (0.80)	0.37 (0.69)	0.70 (0.94)	0.54 (0.85)	0.73 (0.94)	0.96 (1.04)	1.30 (1.16)	0.75 (0.92)					
MD (SD)	−0.27 (0.66)	−0.25 (0.65)	−0.34 (0.69)	−0.16 (0.63)	−0.33 (0.79)	−0.33 (0.72)	−0.31 (0.80)	−0.35 (0.69)	−0.39 (0.77)	−0.33 (0.83)					
Time × Milieu											9, 1819	1.03	0.410	1	
Time											1, 1819	249.26	**<0.001**	**<0.001**	0.12
Milieu											9, 1819	25.84	**<0.001**	**<0.001**	0.11
(4) Psychological and somatoform complaints															
T0 M (SD)	1.02 (0.75)	1.07 (0.78)	1.25 (0.80)	1.19 (0.69)	1.50 (0.86)	1.43 (0.78)	1.59 (0.82)	1.71 (0.85)	2.11 (0.80)	1.67 (0.77)					
T1 M (SD)	0.57 (0.68)	0.57 (0.69)	0.76 (0.75)	0.78 (0.70)	1.08 (0.92)	0.92 (0.76)	1.12 (0.85)	1.19 (0.83)	1.69 (0.93)	1.14 (0.80)					
MD (SD)	−0.46 (0.60)	−0.50 (0.65)	−0.48 (0.56)	−0.41 (0.60)	−0.42 (0.70)	−0.51 (0.63)	−0.47 (0.67)	−0.51 (0.63)	−0.42 (0.66)	−0.53 (0.72)					
Time × Milieu											9, 1819	0.79	0.628	1	
Time											1, 1819	756.94	**<0.001**	**<0.001**	0.29
Milieu											9, 1819	37.91	**<0.001**	**<0.001**	0.16
(5) Psychological well-being															
T0 M (SD)	2.28 (0.80)	2.33 (0.83)	2.50 (0.82)	2.44 (0.70)	2.61 (0.81)	2.62 (0.71)	2.68 (0.75)	2.85 (0.74)	3.03 (0.61)	2.69 (0.65)					
T1 M (SD)	1.39 (0.80)	1.37 (0.83)	1.56 (0.82)	1.62 (0.77)	1.87 (0.95)	1.73 (0.85)	1.96 (0.89)	2.10 (0.92)	2.41 (0.85)	1.91 (0.87)					
MD (SD)	−0.89 (0.77)	−0.95 (0.83)	−0.94 (0.87)	−0.82 (0.78)	−0.73 (0.86)	−0.89 (0.79)	−0.72 (0.80)	−0.75 (0.79)	−0.62 (0.74)	−0.78 (0.80)					
Time × Milieu											9, 1819	3.30	**<0.001**	**0.005**	0.02
Time											1, 1819	1489.33	**<0.001**	**<0.001**	0.45
Milieu											9, 1819	27.46	**<0.001**	**<0.001**	0.12
(6) Interactional difficulties															
T0 M (SD)	1.60 (1.00)	1.57 (1.06)	1.77 (0.92)	1.68 (0.83)	1.97 (0.95)	1.85 (0.94)	2.04 (0.95)	2.16 (0.96)	2.36 (0.94)	2.09 (0.94)					
T1 M (SD)	1.00 (0.87)	0.86 (0.93)	1.18 (0.91)	1.08 (0.88)	1.48 (1.01)	1.12 (0.92)	1.56 (1.00)	1.60 (1.00)	1.90 (1.02)	1.44 (0.95)					
MD (SD)	−0.60 (0.91)	−0.71 (0.94)	−0.58 (0.87)	−0.60 (0.88)	−0.49 (1.02)	−0.74 (0.86)	−0.47 (0.86)	−0.56 (0.96)	−0.45 (0.87)	−0.65 (1.01)					
Time × Milieu											9, 1819	2.74	**0.004**	**0.036**	0.01
Time											1, 1819	594.16	**<0.001**	**<0.001**	0.25
Milieu											9, 1819	21.26	**<0.001**	**<0.001**	0.10
(7) Self-efficacy															
T0 M (SD)	1.86 (0.86)	1.77 (0.90)	2.12 (0.87)	1.87 (0.87)	2.16 (0.94)	2.25 (0.83)	2.24 (0.85)	2.62 (0.98)	2.80 (0.75)	2.44 (0.79)					
T1 M (SD)	1.30 (0.86)	1.11 (0.82)	1.46 (0.93)	1.46 (0.83)	1.69 (0.92)	1.67 (0.93)	1.83 (0.94)	2.07 (1.02)	2.34 (0.78)	1.86 (0.89)					
MD (SD)	−0.56 (0.80)	−0.65 (0.81)	−0.66 (0.77)	−0.41 (0.87)	−0.47 (0.86)	−0.59 (0.87)	−0.41 (0.94)	−0.55 (0.77)	−0.46 (0.77)	−0.58 (0.85)					
Time × Milieu											9, 1819	1.79	0.066	0.657	
Time											1, 1819	589.55	**<0.001**	**<0.001**	0.25
Milieu											9, 1819	34.45	**<0.001**	**<0.001**	0.15
(8) Activity and Participation															
T0 M (SD)	1.90 (0.83)	1.82 (0.87)	2.01 (0.83)	2.09 (0.79)	2.14 (0.78)	2.24 (0.79)	2.17 (0.79)	2.41 (0.87)	2.46 (0.76)	2.31 (0.76)					
T1 M (SD)	1.41 (0.82)	1.24 (0.97)	1.44 (0.96)	1.59 (0.94)	1.89 (0.92)	1.66 (0.97)	1.85 (0.94)	1.84 (0.98)	2.24 (0.86)	1.87 (0.93)					
MD (SD)	−0.49 (0.87)	−0.58 (0.81)	−0.57 (0.83)	−0.50 (0.90)	−0.25 (0.84)	−0.57 (0.92)	−0.32 (0.89)	−0.58 (0.82)	−0.23 (0.86)	−0.44 (0.92)					
Time × Milieu											9, 1819	4.94	**<0.001**	**<0.001**	0.02
Time											1, 1819	387.92	**<0.001**	**<0.001**	0.18
Milieu											9, 1819	17.93	**<0.001**	**<0.001**	0.08
(9) Social support															
T0 M (SD)	1.51 (0.80)	1.33 (0.84)	1.64 (0.81)	1.60 (0.81)	1.65 (0.81)	1.48 (0.77)	1.68 (0.86)	1.59 (0.83)	1.85 (0.93)	1.81 (0.90)					
T1 M (SD)	1.43 (0.82)	1.30 (0.76)	1.46 (0.80)	1.54 (0.81)	1.62 (0.87)	1.41 (0.81)	1.59 (0.91)	1.57 (0.84)	1.82 (0.88)	1.77 (0.88)					
MD (SD)	−0.07 (0.73)	−0.04 (0.72)	−0.18 (0.73)	−0.05 (0.72)	−0.03 (0.67)	−0.07 (0.63)	−0.09 (0.75)	−0.02 (0.70)	−0.03 (0.67)	−0.05 (0.66)					
Time × Milieu											9, 1819	0.47	0.898	1	
Time											1, 1819	12.38	**<0.001**	**0.004**	0.01
Milieu											9, 1819	8.79	**<0.001**	**<0.001**	0.04
(10) Social stress															
T0 M (SD)	1.56 (0.84)	1.42 (0.84)	1.69 (0.86)	1.67 (0.77)	1.77 (0.77)	1.63 (0.74)	1.77 (0.81)	1.81 (0.83)	1.97 (0.85)	1.91 (0.80)					
T1 M (SD)	1.46 (0.78)	1.29 (0.77)	1.63 (0.81)	1.52 (0.68)	1.74 (0.77)	1.55 (0.77)	1.77 (0.83)	1.71 (0.85)	1.94 (0.84)	1.79 (0.73)					
MD (SD)	−0.10 (0.75)	−0.13 (0.70)	−0.06 (0.62)	−0.15 (0.78)	−0.03 (0.71)	−0.09 (0.70)	−0.01 (0.71)	−0.09 (0.72)	−0.03 (0.78)	−0.11 (0.69)					
Time × Milieu											9, 1819	0.79	0.627	1	
Time											1, 1819	17.82	**<0.001**	**<0.001**	0.01
Milieu											9, 1819	11.59	**<0.001**	**<0.001**	0.05

EST, Established Milieu; LIB, Liberal Intellectual Milieu; PER, Performer Milieu; COS, Cosmopolitan Avant-garde Milieu; MOD, Modern Mainstreamer Milieu; SOC, Social Ecological Milieu; ADA, Adaptive Navigator Milieu; TRA, Traditional Milieu; PRE, Precarious Milieu; HED, Hedonist Milieu; BDI-II, Beck Depression Inventory; HEALTH-49, Hamburg Modules for the Assessment of Psychosocial Health in Clinical Practice; M, mean; SD, standard deviation; MD, mean difference; df, number of degrees of freedom; *F*, F-statistic; *p*, value of *p*; *p*_adj_, Bonferroni adjusted value of *p*; η^2^_part_, partial eta-squared with η^2^_part_ ≥ 0.01: weak effect, η^2^_part_ ≥ 0.06: medium effect, η^2^_part_ ≥ 0.14: strong effect.

a A higher score indicates a higher burden.

b Number of patients responding to BDI-II resp. HEALTH-49 and their percentage out of the total sample of *N* = 2,000.

c Number of respondents from the corresponding milieu and percentage out of the total number of patients from this milieu.

Examination of the main effects showed that they were significant for both time and milieu, each with *p* < 0.001, implicating that depressiveness decreased significantly and that patients from different milieus differed significantly from each other. The effects were strong with η^2^_part_ = 0.47 for time and η^2^_part_ = 0.14 for milieu. The Games-Howell post-hoc test revealed twenty significant group differences, each with *p* < 0.001. Patients from the Established Milieu and the Liberal Intellectual Milieu showed significantly lower values than patients from six other milieus, most of whom are, according to the Sinus model, in a less socioeconomically privileged position. Patients from the Precarious Milieu showed significantly higher values than patients from eight other milieus, with the exception of patients from the Traditional Milieu. Detailed results of the post-hoc test are reported in Supplementary Table S1.

**Figure 3:**
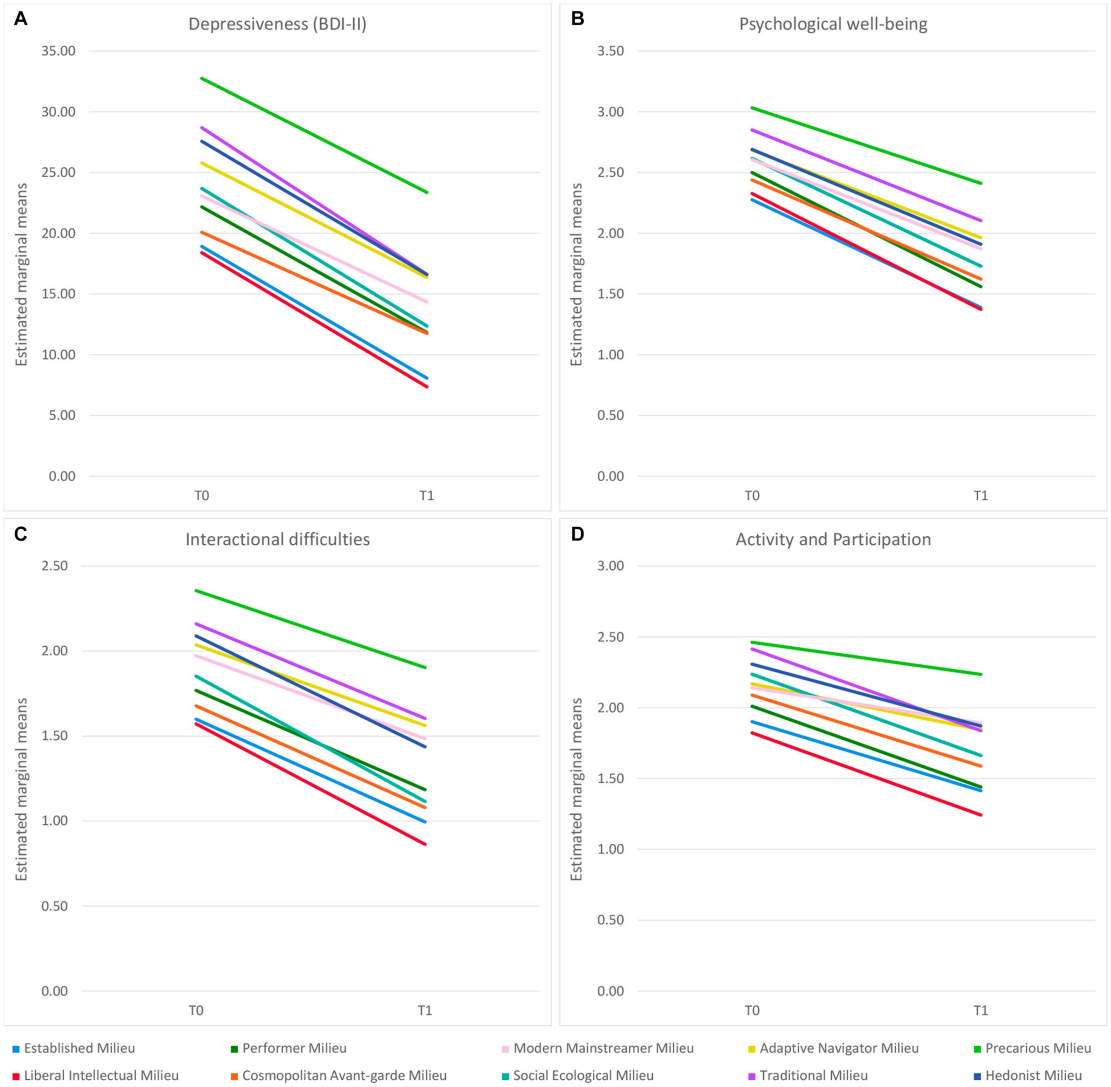
Differences in symptom and impairment improvement between patients from different milieus over the course of the five-week rehabilitation treatment in BDI-II Depressiveness **(A)** and HEALTH-49 Psychological well-being **(B)**, Interactional difficulties **(C)**, and Activity and Participation **(D)**. The (adjusted) values of *p* of the interaction effects can be found in Table 2 and the results of the post-hoc tests on pre-post differences in Supplementary Table S2.

We further found a statistically significant interaction between test time and milieu, i.e., score decrease from T0 to T1 differed significantly due to milieu: time × milieu *F*(9, 1822) = 2.50, *p* = 0.008 (cf. Figure 3). The effect was weak with η^2^_part_ = 0.01. In the Games-Howell post-hoc test, there were no significant group differences between patients from different milieus in terms of pre-post differences (cf. Supplementary Table S2).

#### 3.2.2. Psychosocial health (HEALTH-49)

Comparing among different scales, patients from most of the milieus had the highest impairment in Psychological well-being at both T0 and T1. Lowest symptom load was generally shown on the Phobic anxiety scale by patients from all milieus at both times. Patients from the Precarious Milieu had the highest scores on all ten scales at both time points, followed by patients from the Traditional Milieu or the Hedonist Milieu on most scales. Lowest scores were generally shown by patients from the Liberal Intellectual Milieu and the Established Milieu. Similar to BDI-II results, patients from the Precarious Milieu still had a higher burden on all HEALTH-49 scales after rehabilitation than patients from, according to the Sinus model, more socioeconomically privileged milieus had at baseline before the intervention (cf. Table 2).

On all scales, main effects for time and milieu were significant with *p*_adj_ < 0.001 (exception: Social support scale with *p*_adj_ for time = 0.004). The respective η^2^_part_ ranged between 0.01 and 0.45, indicating weak to strong effects on the different scales. For the main effect of time, the effects were strong for seven of the scales, whereas weak effect sizes were found for the Social support and Social stress scales. Effect sizes for the main effect of milieu were also weak only for these two scales, reflecting less relevant differences between patients from different milieus in these areas. Otherwise, effect sizes ranged from medium (on five scales) to strong (on three scales). In the Tukey and Games-Howell post-hoc tests, significant group differences with *p* ≤ 0.001 were found on all ten HEALTH-49 scales. As in the BDI-II, patients from the Established Milieu and the Liberal Intellectual Milieu had significantly lower values than patients from, according to the Sinus model, less socioeconomically privileged milieus. Apart from the Social support and Social stress scales, patients from the Precarious Milieu showed significantly higher values than patients from at least seven, up to all nine other milieus. Only five and nine significant group differences were found for the Social support and Social stress scales, respectively; these showed a similar pattern to those found for the other scales (cf. Supplementary Table S1).

On seven out of the ten scales, there were no significant interactions, indicating that impairment decreased similarly across all milieus. On three scales, there were significant interaction effects between test time and milieu, i.e., impairment decreased to different degrees depending on the milieu (cf. Figure 3). These were Psychological well-being [time × milieu *F*(9, 1819) = 3.30, *p*_adj_ = 0.005, η^2^_part_ = 0.02], Interactional difficulties [time × milieu *F*(9, 1819) = 2.74, *p*_adj_ = 0.036, η^2^_part_ = 0.01] and Activity and Participation [time × milieu *F*(9, 1819) = 4.94, *p*_adj_ < 0.001, η^2^_part_ = 0.02]. The sizes of η^2^_part_ corresponded to weak effects in all cases. For the first two of these scales, Tukey post-hoc tests yielded no significant group differences between patients from different milieus with respect to pre-post differences. In the Tukey post-hoc test for the Activity and Participation scale, patients from the Social Ecological Milieu improved significantly more than those from the Modern Mainstreamer Milieu (−0.32, *p* = 0.001) and the Precarious Milieu (−0.35, *p* < 0.001; cf. Supplementary Table S2).

## 4. Discussion

### 4.1. Summary of the results

Our study investigated the associations between social milieu and the severity of psychological symptoms and psychosocial impairments as well as treatment outcome of patients in two psychosomatic rehabilitation clinics in Germany. Empirical Sinus milieus were applied as a model for social milieus and symptoms and impairments were assessed by BDI-II (*N* = 1,832) and HEALTH-49 (*N* = 1,829). Milieu distribution was not representative for the overall population of Germany and the Social Ecological Milieu and the Precarious Milieu were overrepresented in both clinics. We found significant differences between patients from different milieus in symptom severity and impairment with mainly medium to strong effects. Patients from the Precarious Milieu had the highest severity of depressive symptoms in the BDI-II and the highest impairment on all HEALTH-49 scales at T0 and T1. Patients from the Precarious Milieu, the Established Milieu and the Liberal Intellectual Milieu were involved in most of the significant group differences, with the former showing higher burdens and patients from the latter two milieus showing lower burdens than patients from other milieus. Over the course of rehabilitation, patients from all milieus improved significantly in all domains with mainly strong effects. Significant differences in symptom improvement were found between patients from different milieus in BDI-II (Depressiveness) and on three HEALTH-49 scales (Psychological well-being, Interactional difficulties, Activity and Participation). Pre-post differences differed significantly only for the latter scale, where patients from the Social Ecological Milieu showed greater improvement than those from the Modern Mainstreamer Milieu and the Precarious Milieu. However, the weak effect sizes of the interactions generally imply that the differences were rather minor and that the improvement was thus overall comparable, just similar to how it was the case in all other domains for patients from all milieus. In all domains, patients from the Precarious Milieu retained higher symptoms and impairment at T1 than patients from, according to the Sinus model, more socioeconomically privileged milieus had at T0.

### 4.2. Comparison to other studies on inequalities in mental health care

In the following, we compare our results to other studies on inequalities in psychosomatic rehabilitation and psychiatric care. In some studies, low socioeconomic status was associated with higher claim of psychiatric services utilisation (37) and higher likelihood of being (compulsorily) admitted to psychiatric in-patient care (38). In others, low socioeconomic status was related to lower rates of seeing a psychiatrist (39), higher reports of personal barriers to access mental health services (e.g., having language barriers, being afraid to ask for help; 40) and limited access to outpatient psychotherapy in Germany (41) – which has the potential to prevent the need for rehabilitation. In a population-representative survey in Germany, socioeconomic status did not show any significant associations with the use of psychotherapeutic or psychiatric services when controlling for medical need (42). This ambiguity of results with respect to socioeconomic status can probably be attributed partly to the specific differences in study subjects and methodologies. Against the described background, however, it is not surprising that milieus with comparable socioeconomic conditions according to the Sinus model were represented differently in our study. For example, of the milieus with the most disadvantaged socioeconomic position, only the Precarious Milieu was overrepresented, while the Hedonist Milieu and the Traditional Milieu were on average or underrepresented, depending on the clinic sample.

In the study of Hofreuter-Gätgens et al., socioeconomically privileged patients had the least impairment in most areas of subjective health at the beginning of rehabilitation (14). In line with these results, patients from the Established Milieu, the Liberal Intellectual Milieu, the Performer Milieu and the Cosmopolitan Avant-garde Milieu, all of which are in a socioeconomically privileged position according to the Sinus model, showed the lowest symptom severity and impairment in our study. In the study of Deck, the so-called lower class was the most impaired group at the beginning of rehabilitation concerning different aspects of subjective health (18). In our study, the highest symptom severity and impairment were shown by patients from the Precarious Milieu, who were not able to compensate for the initial differences and remained with greater impairments, as did the lower class in Deck’s study. At the same time, our results were more differentiated and suggested more than just a status, class or stratification gradient. Thus, patients from different milieus, which are characterized by similar socioeconomic conditions according to the Sinus model, showed different levels of severity both at the beginning and at the end of rehabilitation. This was also evident in the improvement on the Activity and Participation scale, where patients from the Social Ecological Milieu benefited more than patients from the Modern Mainstreamer Milieu, who are socioeconomically similarly situated according to the Sinus model. To summarize, accessibility, symptom severity and to some extent improvement appear to be associated with other milieu-specific characteristics in addition to socioeconomic factors, which should be considered when describing existing inequalities.

### 4.3. Milieu-specific reflections on the basis of the study results

In the following, we present some exemplary milieu-specific reflections on our findings. To the best of our research and knowledge, there are almost no studies in the medical field and none at all in psychosomatic rehabilitation that previously used a milieu approach. Accordingly, derivations from or references to already existing literature can be made only to a limited extent. Our considerations are mainly based on the content characterizations of the Sinus milieus by the Sinus institute (cf. chapter 2.2.), which also means that further studies and empirical evidence are required to proof these theoretical hypotheses.

The lack of representativity of the milieu distribution compared to the overall population may indicate that psychosomatic rehabilitation does not reach and appeal to patients from all milieus equally. The overrepresentation of the Social Ecological Milieu and the Precarious Milieu in both clinics might, however, have different reasons. According to the Sinus model, patients from the Social Ecological Milieu are usually engaged in reflecting their feelings and behavior and in acquiring new methods to be in balance with themselves and their environment. The therapy setting in dedicated clinics in calm environments and the applied therapies including relaxation methods, creative therapy and socio-medical counseling might sound particularly attractive to them. On the other hand, according to the Sinus model, patients from the Precarious Milieu tend to be subjects to social exclusion, hidden discrimination and economic deprivation. These factors are likely to cause and increase psychosocial stress, which has been described as an essential mediator between deprived social conditions and adverse health outcomes (43). Increased mental morbidity and long-term impairment might then lead to higher admission rates to psychosomatic rehabilitation in the Precarious Milieu. By contrast, various reasons for the underrepresentation of some milieus in the patient collective are conceivable. Patients from the Traditional Milieu, which is underrepresented in both clinics, belong to one of the milieus that are, according to the Sinus model, primarily prevalent in older generations. Accordingly, people from this milieu likely tend to be of higher age and retired status, which may render them less suitable for admission to rehabilitation, as one major reason for the German pension insurance to grant payment for the treatment is to maintain earning capacity. Another possible reason may be that people from the underrepresented milieus are partly sceptical about psychotherapy, which is the core element of psychosomatic rehabilitation. This consideration is based on the results of population-representative surveys in Germany that showed different attitudes towards psychotherapy in different population groups. For instance, more negative attitudes were found among men (44, 45) and people with lower levels of formal education (45). In addition, more than a quarter of respondents categorically ruled out psychotherapy for themselves (44). Speerforck and Schomerus suggested that stigmatizing attitudes towards and different acceptances of mental health services might differ across social milieus, leading to different risks of underuse (46).

Regarding the single milieu-specific differences in treatment outcome of Activity and Participation, we would like to present the following assumptions. The applied therapies in psychosomatic rehabilitation might suit especially well to patients from the Social Ecological Milieu due to their specific values and needs, as described above. Practising mindfulness, learning to deal with oneself in an even more sustainable way and the slow pace in the quiet rehabilitation setting may especially help these patients increase their self-activation and participation opportunities. On the other hand, regarding the Precarious Milieu, patients could be affected by social exclusion and disadvantage in rehabilitation, as it tends to happen in their everyday life, according to the Sinus model. Perceived social status discrimination, known to be associated with psychological symptoms (47), might also be negatively associated with improvement. Moreover, since a great distance to intellectuality, know-it-all attitude and creativity is described for the Precarious Milieu, several therapy formats such as cognitive psychotherapy, health counseling and creative therapies might be perceived by these patients as inappropriate, patronizing and too abstract. This could then further reinforce resignation prevalent in the milieu, additionally preventing higher levels of activity and participation. The social position in the model also reveals the limited sociocultural and material resources of the Precarious Milieu, which, for example, could continuously restrict the coping capacities of the patients in our sample, despite positive effects of the treatment itself.

Concerning all milieus, the generally low improvements on the HEALTH-49 scales Social support and Social stress might be due to the fact that the corresponding items are predominantly influenced by contextual factors that are hardly affected by rehabilitation. Given the distinct characteristics of the milieus, further differences in improvement beyond those we found would have been conceivable, for example, a comparably stronger benefit of patients from the Liberal Intellectual Milieu. For these patients, according to the Sinus model, it is usually very important to do something for their health, shape life in a holistic way, act autonomously and realize themselves. Rehabilitation with its holistic, sophisticated therapy offer and its approach of strengthening self-efficacy may fit these prerequisites particularly well. In addition, patients from this milieu also have good preconditions for treatment success due to their socioeconomic privileges according to the Sinus model.

In general, patients from all milieus improved over the course of the treatment and essentially to a similar degree, which indicates overall success of the rehabilitation. Although the improvements themselves were comparable, differences in the severity of symptoms and impairments that existed at the beginning of rehabilitation remained. Our study results highlight that the observed differences between patients from different social milieus could be related to a variety of factors and not solely to socioeconomic determinants. In our discussion, we have provided examples of hypotheses that demonstrate the complexity of potential relationships and mediating mechanisms. Such considerations would hardly be possible on the basis of socioeconomic factors alone. The question remains open as to which of the various individual factors included in the model are independently associated with symptom severity and treatment outcome and, more generally, whether and, if so, in what direct or indirect ways they exert causal influence, which could also be of interest for further studies.

### 4.4. Future perspectives in psychosomatic rehabilitation

The demand for psychosomatic rehabilitation is projected to increase in the future (48, 49) and we do not expect social inequalities to decline substantially in the short term. In addition to improvements at the level of care structure, perhaps the particular care services should be adapted and communicated in a way that is more appropriate and appealing to persons from different milieus (46). In any case, our findings argue for even greater and especially systematic inclusion of socioeconomic and sociocultural aspects in psychosomatic rehabilitation to address and reduce structural inequalities. To improve and maintain equal access, quality and effectiveness of the treatment, the social milieu approach could be incorporated into therapy planning and implementation. With an appropriate approach, socioeconomic and sociocultural factors could be systematically recorded and binding rules established for their quality-assured consideration. In this way, structural disadvantages of specific patient groups due to institutions or therapists could be alleviated. In practical implementation, the assignment of patients to therapy groups and specific therapy content could be more closely aligned with the different socioeconomic and sociocultural stresses and resources of patients. Not only may it be unjust to offer the same treatment to different patients with unequal preconditions (14), but it could also reinforce existing inequalities. Therefore, disadvantaged social groups should be considered with particular care, which is not yet the case in psychosomatic rehabilitation. Accordingly, the social milieu could also be used to identify disadvantaged patients and to develop treatment formats that address their specific demands. In addition, the duration of rehabilitation, the intensity of therapy plans and the design of rehabilitation aftercare could be adjusted. However, inequality affects not only rehabilitation and health care, but life chances in general and it cannot be changed without broader, integrated policy efforts (50).

### 4.5. Strengths and limitations

Strengths of the study design were the standardized testing, the high response rates (91.60% out of the total sample for BDI-II and 91.45% for HEALTH-49) and the application of instruments of good psychometric quality. In both, BDI-II and HEALTH-49, patients from all milieus clearly showed lower scores at the end of rehabilitation compared to the beginning, which speaks for the instruments’ high sensitivity to change. All ten milieus were represented, in part due to the large sample size (*N* = 2,000). It can be assumed that the collective of patients in psychosomatic rehabilitation in Germany was well represented in the sample. In addition, the respective group sizes of the different milieus were large enough for good statistical power. Importantly, the large total sample size was not chosen to foster overestimation of the statistical effects, which is supported by the fact that the single milieu group sizes were comparably small, such as *N* = 76 in the smallest milieu (the Performer Milieu) and *N* = 412 in the largest milieu (the Social Ecological Milieu; *N* here reflects the number of patients from the milieu for whom an evaluable BDI-II and/or HEALTH-49 was available). The examination of samples of two clinics in different federal states and with different providers further improved representation of the overall sociodemographic structure and milieu distribution among patients in psychosomatic rehabilitation in Germany. Altogether, with the included instruments, patient characteristics and milieu model, we considered several widely used and recommended public mental health indicators (e.g., prevalence of mental disorders, mental health risks such as income inequality in society, treatment success, mental health resources such as self-efficacy, positive mental health indicators such as well-being; 51).

The newly introduced milieu approach extends former research on health inequalities by sociocultural differentiation in terms of specific knowledge, perceptions, values, attitudes and behaviors of patients (52). As described above, the inclusion of such factors has the potential to deliver a more comprehensive understanding compared to approaches that merely focus on socioeconomic aspects. Nevertheless, as common when applying models to describe reality, only a specific selection of factors with potential associations could be analyzed by using the Sinus model. For instance, although sociodemographic characteristics are implicitly included (e.g., in some cases, people of certain age groups are more frequently represented in a milieu than those of other age groups), the model itself does not allow for the analysis of individual factors such as age and gender – even though they may have the potential to independently cause and increase inequalities. Looking at individual factors, on the other hand, would not necessarily be in line with the approach and goal of the milieu model, which integrates several factors in order to be able to describe large social groups on the basis of various dimensions and social realities. The characterization of the different Sinus milieus enabled us to hypothesize possible explanations for assumed relationships between social factors and health outcomes. Thus, the application of the milieu model in the context of this study added value to the description of observable differences, particularly at the conceptual and theoretical levels. At the same time, the study made it possible to derive concrete practical implications for the care setting of psychosomatic rehabilitation. However, our considerations require empirical verification, especially in light of the fact that hardly any studies in the medical field have used a milieu approach so far. Another limitation is that methodical details of the milieu assignment are not published by the Sinus institute for intellectual property reasons (22). This limits reproducibility of the study, but as the Sinus milieus are a validated model that is commonly used in milieu research across Europe (24), this seems tolerable in favor of the high reliability and actuality of the model. Furthermore, our study did not include long-term treatment outcomes which might differ from the immediate rehabilitation effects due to the re-emergence of contextual stressors in everyday life and differences in the consolidation of new skills across patients from different milieus.

In summary, this study has shown differences between patients from different social milieus in terms of representation in psychosomatic rehabilitation, severity of psychological symptoms and psychosocial impairments, and to some extent treatment outcomes regarding improvement. Besides socioeconomic factors, milieu-specific sociocultural habits, psychosocial needs and therapeutic demands should be considered in therapy planning and implementation, for which further research is necessary.

## Data availability statement

The raw data supporting the conclusions of this article will be made available by the authors, without undue reservation.

## Ethics statement

The studies involving human participants were reviewed and approved by the Landesärztekammer Brandenburg (Brandenburg State Medical Association), Geschäftsstelle Cottbus. The patients/participants provided their written informed consent to participate in this study.

## Author contributions

MB and VK conceptualized the study. HK-M ran statistical analyses, interpreted results, wrote the manuscript, and generated tables and figures. LP reviewed statistical analyses. LP, MB, and VK provided feedback on the manuscript. All authors contributed to the article and approved the submitted version.

## Funding

The study was funded by the Deutsche Rentenversicherung Bund (Federal German Pension Agency), grant number 0421/40-64-50-01.

## Conflict of interest

The authors declare that the research was conducted in the absence of any commercial or financial relationships that could be construed as a potential conflict of interest.

## Publisher’s note

All claims expressed in this article are solely those of the authors and do not necessarily represent those of their affiliated organizations, or those of the publisher, the editors and the reviewers. Any product that may be evaluated in this article, or claim that may be made by its manufacturer, is not guaranteed or endorsed by the publisher.
