# Supplementary material for: Milieu-specific differences in symptom severity and treatment outcome in psychosomatic rehabilitation in Germany
Source: Front Psychiatry. 2023 Aug 15;14:1198146. doi: 10.3389/fpsyt.2023.1198146 (PMC10465793; doi:10.3389/fpsyt.2023.1198146)
Supplement: Supplementary file 1 [file Table_1.docx]

Supplementary Material

Milieu-specific differences in symptom severity and treatment outcome in psychosomatic rehabilitation in Germany

Henrika Kleineberg-Massuthe^*^, Lilia Papst, Markus Bassler, Volker Köllner

*** Correspondence:** Henrika Kleineberg-Massuthe: henrika.kleineberg-massuthe@charite.de

**Supplementary Table 1.** Group differences for the main effect of milieu in Tukey and Games-Howell post-hoc tests for the BDI-II and the ten scales of the HEALTH-49.

|  |  |  |  |  |  | **99.9% CI** | |
| --- | --- | --- | --- | --- | --- | --- | --- |
|  | **Milieu (A)** | **Milieu (B)** | **MD (A-B)** | **SE** | ***p*** | **LL** | **UL** |
| **BDI-II (*N* = 1,832)** | | | | | | | |
| **Games-Howell post-hoc test** | | | | | | | |
|  | Established Milieu (*N* = 144) | Liberal Intellectual Milieu | 0.61 | 1.04 | 1.000 | -3.84 | 5.07 |
|  |  | Performer Milieu | -3.51 | 1.49 | 0.364 | -10.02 | 3.01 |
|  |  | Cosmopolitan Avant-garde Milieu | -2.42 | 1.27 | 0.659 | -7.89 | 3.05 |
|  |  | Adaptive Navigator Milieu | -7.59* | 1.16 | **< 0.001** | -12.54 | -2.63 |
|  |  | Social Ecological Milieu | -4.53* | 0.92 | **< 0.001** | -8.50 | -0.57 |
|  |  | Modern Mainstreamer Milieu | -5.22* | 1.12 | **< 0.001** | -10.00 | -0.43 |
|  |  | Traditional Milieu | -9.17* | 1.48 | **< 0.001** | -15.58 | -2.75 |
|  |  | Precarious Milieu | -14.57* | 1.10 | **< 0.001** | -19.27 | -9.86 |
|  |  | Hedonist Milieu | -8.60* | 1.08 | **< 0.001** | -13.23 | -3.96 |
|  | Liberal Intellectual Milieu (*N* = 157) | Established Milieu | -0.61 | 1.04 | 1.000 | -5.07 | 3.84 |
|  |  | Performer Milieu | -4.12 | 1.43 | 0.125 | -10.42 | 2.18 |
|  |  | Cosmopolitan Avant-garde Milieu | -3.04 | 1.20 | 0.258 | -8.23 | 2.16 |
|  |  | Adaptive Navigator Milieu | -8.20* | 1.08 | **< 0.001** | -12.84 | -3.56 |
|  |  | Social Ecological Milieu | -5.15* | 0.83 | **< 0.001** | -8.70 | -1.60 |
|  |  | Modern Mainstreamer Milieu | -5.83* | 1.04 | **< 0.001** | -10.29 | -1.37 |
|  |  | Traditional Milieu | -9.78* | 1.42 | **< 0.001** | -15.97 | -3.59 |
|  |  | Precarious Milieu | -15.18* | 1.02 | **< 0.001** | -19.55 | -10.82 |
|  |  | Hedonist Milieu | -9.21* | 1.00 | **< 0.001** | -13.50 | -4.92 |
|  | Performer Milieu (*N* = 76) | Established Milieu | 3.51 | 1.49 | 0.364 | -3.01 | 10.02 |
|  |  | Liberal Intellectual Milieu | 4.12 | 1.43 | 0.125 | -2.18 | 10.42 |
|  |  | Cosmopolitan Avant-garde Milieu | 1.08 | 1.61 | 1.000 | -5.92 | 8.08 |
|  |  | Adaptive Navigator Milieu | -4.08 | 1.52 | 0.191 | -10.72 | 2.56 |
|  |  | Social Ecological Milieu | -1.03 | 1.35 | 0.999 | -7.02 | 4.96 |
|  |  | Modern Mainstreamer Milieu | -1.71 | 1.49 | 0.979 | -8.23 | 4.81 |
|  |  | Traditional Milieu | -5.66 | 1.78 | 0.054 | -13.38 | 2.07 |
|  |  | Precarious Milieu | -11.06* | 1.48 | **< 0.001** | -17.53 | -4.60 |
|  |  | Hedonist Milieu | -5.09 | 1.47 | 0.024 | -11.51 | 1.33 |
|  | Cosmopolitan Avant-garde Milieu (*N* = 97) | Established Milieu | 2.42 | 1.27 | 0.659 | -3.05 | 7.89 |
|  |  | Liberal Intellectual Milieu | 3.04 | 1.20 | 0.258 | -2.16 | 8.23 |
|  |  | Performer Milieu | -1.08 | 1.61 | 1.000 | -8.08 | 5.92 |
|  |  | Adaptive Navigator Milieu | -5.16 | 1.30 | 0.004 | -10.78 | 0.45 |
|  |  | Social Ecological Milieu | -2.11 | 1.10 | 0.657 | -6.91 | 2.69 |
|  |  | Modern Mainstreamer Milieu | -2.79 | 1.27 | 0.459 | -8.26 | 2.68 |
|  |  | Traditional Milieu | -6.74 | 1.60 | 0.002 | -13.65 | 0.17 |
|  |  | Precarious Milieu | -12.14* | 1.25 | **< 0.001** | -17.55 | -6.74 |
|  |  | Hedonist Milieu | -6.17* | 1.24 | **< 0.001** | -11.51 | -0.83 |
|  | Adaptive Navigator Milieu (*N* = 182) | Established Milieu | 7.59* | 1.16 | **< 0.001** | 2.63 | 12.54 |
|  |  | Liberal Intellectual Milieu | 8.20* | 1.08 | **< 0.001** | 3.56 | 12.84 |
|  |  | Performer Milieu | 4.08 | 1.52 | 0.191 | -2.56 | 10.72 |
|  |  | Cosmopolitan Avant-garde Milieu | 5.16 | 1.30 | 0.004 | -0.45 | 10.78 |
|  |  | Social Ecological Milieu | 3.06 | 0.97 | 0.057 | -1.11 | 7.23 |
|  |  | Modern Mainstreamer Milieu | 2.37 | 1.16 | 0.566 | -2.58 | 7.33 |
|  |  | Traditional Milieu | -1.58 | 1.51 | 0.989 | -8.12 | 4.97 |
|  |  | Precarious Milieu | -6.98* | 1.14 | **< 0.001** | -11.86 | -2.10 |
|  |  | Hedonist Milieu | -1.01 | 1.13 | 0.997 | -5.82 | 3.80 |
|  | Social Ecological Milieu (*N* = 412) | Established Milieu | 4.53* | 0.92 | **< 0.001** | 0.57 | 8.50 |
|  |  | Liberal Intellectual Milieu | 5.15* | 0.83 | **< 0.001** | 1.60 | 8.70 |
|  |  | Performer Milieu | 1.03 | 1.35 | 0.999 | -4.96 | 7.02 |
|  |  | Cosmopolitan Avant-garde Milieu | 2.11 | 1.10 | 0.657 | -2.69 | 6.91 |
|  |  | Adaptive Navigator Milieu | -3.06 | 0.97 | 0.057 | -7.23 | 1.11 |
|  |  | Modern Mainstreamer Milieu | -0.68 | 0.93 | 0.999 | -4.64 | 3.28 |
|  |  | Traditional Milieu | -4.63 | 1.34 | 0.025 | -10.50 | 1.24 |
|  |  | Precarious Milieu | -10.03* | 0.90 | **< 0.001** | -13.89 | -6.18 |
|  |  | Hedonist Milieu | -4.06* | 0.88 | **< 0.001** | -7.83 | -0.30 |
|  | Modern Mainstreamer Milieu (*N* = 196) | Established Milieu | 5.22* | 1.12 | **< 0.001** | 0.43 | 10.00 |
|  |  | Liberal Intellectual Milieu | 5.83* | 1.04 | **< 0.001** | 1.37 | 10.29 |
|  |  | Performer Milieu | 1.71 | 1.49 | 0.979 | -4.81 | 8.23 |
|  |  | Cosmopolitan Avant-garde Milieu | 2.79 | 1.27 | 0.459 | -2.68 | 8.26 |
|  |  | Adaptive Navigator Milieu | -2.37 | 1.16 | 0.566 | -7.33 | 2.58 |
|  |  | Social Ecological Milieu | 0.68 | 0.93 | 0.999 | -3.28 | 4.64 |
|  |  | Traditional Milieu | -3.95 | 1.48 | 0.196 | -10.37 | 2.47 |
|  |  | Precarious Milieu | -9.35* | 1.10 | **< 0.001** | -14.06 | -4.65 |
|  |  | Hedonist Milieu | -3.38 | 1.08 | 0.060 | -8.01 | 1.25 |
|  | Traditional Milieu (*N* = 98) | Established Milieu | 9.17* | 1.48 | **< 0.001** | 2.75 | 15.58 |
|  |  | Liberal Intellectual Milieu | 9.78* | 1.42 | **< 0.001** | 3.59 | 15.97 |
|  |  | Performer Milieu | 5.66 | 1.78 | 0.054 | -2.07 | 13.38 |
|  |  | Cosmopolitan Avant-garde Milieu | 6.74 | 1.60 | 0.002 | -0.17 | 13.65 |
|  |  | Adaptive Navigator Milieu | 1.58 | 1.51 | 0.989 | -4.97 | 8.12 |
|  |  | Social Ecological Milieu | 4.63 | 1.34 | 0.025 | -1.24 | 10.50 |
|  |  | Modern Mainstreamer Milieu | 3.95 | 1.48 | 0.196 | -2.47 | 10.37 |
|  |  | Precarious Milieu | -5.40 | 1.47 | 0.011 | -11.77 | 0.96 |
|  |  | Hedonist Milieu | 0.57 | 1.45 | 1.000 | -5.74 | 6.88 |
|  | Precarious Milieu (*N* = 229) | Established Milieu | 14.57* | 1.10 | **< 0.001** | 9.86 | 19.27 |
|  |  | Liberal Intellectual Milieu | 15.18* | 1.02 | **< 0.001** | 10.82 | 19.55 |
|  |  | Performer Milieu | 11.06* | 1.48 | **< 0.001** | 4.60 | 17.53 |
|  |  | Cosmopolitan Avant-garde Milieu | 12.14* | 1.25 | **< 0.001** | 6.74 | 17.55 |
|  |  | Adaptive Navigator Milieu | 6.98* | 1.14 | **< 0.001** | 2.10 | 11.86 |
|  |  | Social Ecological Milieu | 10.03* | 0.90 | **< 0.001** | 6.18 | 13.89 |
|  |  | Modern Mainstreamer Milieu | 9.35* | 1.10 | **< 0.001** | 4.65 | 14.06 |
|  |  | Traditional Milieu | 5.40 | 1.47 | 0.011 | -0.96 | 11.77 |
|  |  | Hedonist Milieu | 5.97* | 1.07 | **< 0.001** | 1.43 | 10.52 |
|  | Hedonist Milieu (*N* = 241) | Established Milieu | 8.60* | 1.08 | **< 0.001** | 3.96 | 13.23 |
|  |  | Liberal Intellectual Milieu | 9.21* | 1.00 | **< 0.001** | 4.92 | 13.50 |
|  |  | Performer Milieu | 5.09 | 1.47 | 0.024 | -1.33 | 11.51 |
|  |  | Cosmopolitan Avant-garde Milieu | 6.17* | 1.24 | **< 0.001** | 0.83 | 11.51 |
|  |  | Adaptive Navigator Milieu | 1.01 | 1.13 | 0.997 | -3.80 | 5.82 |
|  |  | Social Ecological Milieu | 4.06* | 0.88 | **< 0.001** | 0.30 | 7.83 |
|  |  | Modern Mainstreamer Milieu | 3.38 | 1.08 | 0.060 | -1.25 | 8.01 |
|  |  | Traditional Milieu | -0.57 | 1.45 | 1.000 | -6.88 | 5.74 |
|  |  | Precarious Milieu | -5.97* | 1.07 | **< 0.001** | -10.52 | -1.43 |
| **HEALTH-49 (*N* = 1,829) – (1) Somatoform complaints** | | | | | | | |
| **Games-Howell post-hoc test** | | | | | | | |
|  | Established Milieu (*N* = 144) | Liberal Intellectual Milieu | -0.04 | 0.09 | 1.000 | -0.42 | 0.33 |
|  |  | Performer Milieu | -0.23 | 0.11 | 0.547 | -0.71 | 0.25 |
|  |  | Cosmopolitan Avant-garde Milieu | -0.31 | 0.10 | 0.067 | -0.74 | 0.12 |
|  |  | Adaptive Navigator Milieu | -0.56* | 0.09 | **< 0.001** | -0.94 | -0.18 |
|  |  | Social Ecological Milieu | -0.43* | 0.07 | **< 0.001** | -0.76 | -0.11 |
|  |  | Modern Mainstreamer Milieu | -0.45* | 0.09 | **< 0.001** | -0.84 | -0.07 |
|  |  | Traditional Milieu | -0.53* | 0.11 | **< 0.001** | -1.03 | -0.04 |
|  |  | Precarious Milieu | -1.01* | 0.09 | **< 0.001** | -1.39 | -0.64 |
|  |  | Hedonist Milieu | -0.52* | 0.08 | **< 0.001** | -0.88 | -0.17 |
|  | Liberal Intellectual Milieu (*N* = 157) | Established Milieu | 0.04 | 0.09 | 1.000 | -0.33 | 0.42 |
|  |  | Performer Milieu | -0.19 | 0.11 | 0.793 | -0.66 | 0.29 |
|  |  | Cosmopolitan Avant-garde Milieu | -0.27 | 0.10 | 0.181 | -0.69 | 0.16 |
|  |  | Adaptive Navigator Milieu | -0.52* | 0.09 | **< 0.001** | -0.89 | -0.14 |
|  |  | Social Ecological Milieu | -0.39* | 0.07 | **< 0.001** | -0.70 | -0.08 |
|  |  | Modern Mainstreamer Milieu | -0.41* | 0.09 | **< 0.001** | -0.79 | -0.03 |
|  |  | Traditional Milieu | -0.49* | 0.11 | **0.001** | -0.98 | 0.00 |
|  |  | Precarious Milieu | -0.97* | 0.09 | **< 0.001** | -1.33 | -0.60 |
|  |  | Hedonist Milieu | -0.48* | 0.08 | **< 0.001** | -0.82 | -0.14 |
|  | Performer Milieu (*N* = 76) | Established Milieu | 0.23 | 0.11 | 0.547 | -0.25 | 0.71 |
|  |  | Liberal Intellectual Milieu | 0.19 | 0.11 | 0.793 | -0.29 | 0.66 |
|  |  | Cosmopolitan Avant-garde Milieu | -0.08 | 0.12 | 1.000 | -0.60 | 0.44 |
|  |  | Adaptive Navigator Milieu | -0.33 | 0.11 | 0.088 | -0.81 | 0.15 |
|  |  | Social Ecological Milieu | -0.20 | 0.10 | 0.552 | -0.64 | 0.23 |
|  |  | Modern Mainstreamer Milieu | -0.22 | 0.11 | 0.581 | -0.71 | 0.26 |
|  |  | Traditional Milieu | -0.31 | 0.13 | 0.372 | -0.87 | 0.26 |
|  |  | Precarious Milieu | -0.78* | 0.11 | **< 0.001** | -1.26 | -0.31 |
|  |  | Hedonist Milieu | -0.29 | 0.10 | 0.141 | -0.75 | 0.16 |
|  | Cosmopolitan Avant-garde Milieu (*N* = 97) | Established Milieu | 0.31 | 0.10 | 0.067 | -0.12 | 0.74 |
|  |  | Liberal Intellectual Milieu | 0.27 | 0.10 | 0.181 | -0.16 | 0.69 |
|  |  | Performer Milieu | 0.08 | 0.12 | 1.000 | -0.44 | 0.60 |
|  |  | Adaptive Navigator Milieu | -0.25 | 0.10 | 0.277 | -0.68 | 0.18 |
|  |  | Social Ecological Milieu | -0.12 | 0.09 | 0.922 | -0.50 | 0.26 |
|  |  | Modern Mainstreamer Milieu | -0.14 | 0.10 | 0.919 | -0.58 | 0.29 |
|  |  | Traditional Milieu | -0.22 | 0.12 | 0.715 | -0.75 | 0.31 |
|  |  | Precarious Milieu | -0.70* | 0.10 | **< 0.001** | -1.13 | -0.28 |
|  |  | Hedonist Milieu | -0.21 | 0.09 | 0.412 | -0.62 | 0.19 |
|  | Adaptive Navigator Milieu (*N* = 182) | Established Milieu | -0.56* | 0.09 | **< 0.001** | 0.18 | 0.94 |
|  |  | Liberal Intellectual Milieu | -0.52* | 0.09 | **< 0.001** | 0.14 | 0.89 |
|  |  | Performer Milieu | 0.33 | 0.11 | 0.088 | -0.15 | 0.81 |
|  |  | Cosmopolitan Avant-garde Milieu | 0.25 | 0.10 | 0.277 | -0.18 | 0.68 |
|  |  | Social Ecological Milieu | 0.13 | 0.07 | 0.795 | -0.19 | 0.45 |
|  |  | Modern Mainstreamer Milieu | 0.11 | 0.09 | 0.972 | -0.28 | 0.49 |
|  |  | Traditional Milieu | 0.03 | 0.11 | 1.000 | -0.47 | 0.52 |
|  |  | Precarious Milieu | -0.45* | 0.09 | **< 0.001** | -0.82 | -0.08 |
|  |  | Hedonist Milieu | 0.04 | 0.08 | 1.000 | -0.31 | 0.39 |
|  | Social Ecological Milieu (*N* = 410) | Established Milieu | -0.43* | 0.07 | **< 0.001** | 0.11 | 0.76 |
|  |  | Liberal Intellectual Milieu | -0.39* | 0.07 | **< 0.001** | 0.08 | 0.70 |
|  |  | Performer Milieu | 0.20 | 0.10 | 0.552 | -0.23 | 0.64 |
|  |  | Cosmopolitan Avant-garde Milieu | 0.12 | 0.09 | 0.922 | -0.26 | 0.50 |
|  |  | Adaptive Navigator Milieu | -0.13 | 0.07 | 0.795 | -0.45 | 0.19 |
|  |  | Modern Mainstreamer Milieu | -0.02 | 0.07 | 1.000 | -0.34 | 0.30 |
|  |  | Traditional Milieu | -0.10 | 0.10 | 0.993 | -0.55 | 0.35 |
|  |  | Precarious Milieu | 0.58* | 0.07 | **< 0.001** | -0.88 | -0.27 |
|  |  | Hedonist Milieu | -0.09 | 0.07 | 0.936 | -0.37 | 0.19 |
|  | Modern Mainstreamer Milieu (*N* = 195) | Established Milieu | 0.45* | 0.09 | **< 0.001** | 0.07 | 0.84 |
|  |  | Liberal Intellectual Milieu | 0.41* | 0.09 | **< 0.001** | 0.03 | 0.79 |
|  |  | Performer Milieu | 0.22 | 0.11 | 0.581 | -0.26 | 0.71 |
|  |  | Cosmopolitan Avant-garde Milieu | 0.14 | 0.10 | 0.919 | -0.29 | 0.58 |
|  |  | Adaptive Navigator Milieu | -0.11 | 0.09 | 0.972 | -0.49 | 0.28 |
|  |  | Social Ecological Milieu | 0.02 | 0.07 | 1.000 | -0.30 | 0.34 |
|  |  | Traditional Milieu | -0.08 | 0.11 | 0.999 | -0.57 | 0.41 |
|  |  | Precarious Milieu | 0.56* | 0.09 | **< 0.001** | -0.93 | -0.19 |
|  |  | Hedonist Milieu | -0.07 | 0.08 | 0.998 | -0.42 | 0.28 |
|  | Traditional Milieu (*N* = 98) | Established Milieu | 0.53* | 0.11 | **< 0.001** | 0.04 | 1.03 |
|  |  | Liberal Intellectual Milieu | 0.49* | 0.11 | **0.001** | 0.00 | 0.98 |
|  |  | Performer Milieu | 0.31 | 0.13 | 0.372 | -0.26 | 0.87 |
|  |  | Cosmopolitan Avant-garde Milieu | 0.22 | 0.12 | 0.715 | -0.31 | 0.75 |
|  |  | Adaptive Navigator Milieu | -0.03 | 0.11 | 1.000 | -0.52 | 0.47 |
|  |  | Social Ecological Milieu | 0.10 | 0.10 | 0.993 | -0.35 | 0.55 |
|  |  | Modern Mainstreamer Milieu | 0.08 | 0.11 | 0.999 | -0.41 | 0.57 |
|  |  | Precarious Milieu | -0.48* | 0.11 | **0.001** | -0.96 | 0.01 |
|  |  | Hedonist Milieu | 0.01 | 0.11 | 1.000 | -0.46 | 0.48 |
|  | Precarious Milieu (*N* = 229) | Established Milieu | 1.01* | 0.09 | **< 0.001** | 0.64 | 1.39 |
|  |  | Liberal Intellectual Milieu | 0.97* | 0.09 | **< 0.001** | 0.60 | 1.33 |
|  |  | Performer Milieu | 0.78* | 0.11 | **< 0.001** | 0.31 | 1.26 |
|  |  | Cosmopolitan Avant-garde Milieu | 0.70* | 0.10 | **< 0.001** | 0.28 | 1.13 |
|  |  | Adaptive Navigator Milieu | 0.45* | 0.09 | **< 0.001** | 0.08 | 0.82 |
|  |  | Social Ecological Milieu | 0.58* | 0.07 | **< 0.001** | 0.27 | 0.88 |
|  |  | Modern Mainstreamer Milieu | 0.56* | 0.09 | **< 0.001** | 0.19 | 0.93 |
|  |  | Traditional Milieu | 0.48* | 0.11 | **0.001** | -0.01 | 0.96 |
|  |  | Hedonist Milieu | 0.49* | 0.08 | **< 0.001** | 0.15 | 0.83 |
|  | Hedonist Milieu (*N* = 241) | Established Milieu | 0.52* | 0.08 | **< 0.001** | 0.17 | 0.88 |
|  |  | Liberal Intellectual Milieu | 0.48* | 0.08 | **< 0.001** | 0.14 | 0.82 |
|  |  | Performer Milieu | 0.29 | 0.10 | 0.141 | -0.16 | 0.75 |
|  |  | Cosmopolitan Avant-garde Milieu | 0.21 | 0.09 | 0.412 | -0.19 | 0.62 |
|  |  | Adaptive Navigator Milieu | -0.04 | 0.08 | 1.000 | -0.39 | 0.31 |
|  |  | Social Ecological Milieu | 0.09 | 0.07 | 0.936 | -0.19 | 0.37 |
|  |  | Modern Mainstreamer Milieu | 0.07 | 0.08 | 0.998 | -0.28 | 0.42 |
|  |  | Traditional Milieu | -0.01 | 0.11 | 1.000 | -0.48 | 0.46 |
|  |  | Precarious Milieu | -0.49* | 0.08 | **< 0.001** | -0.83 | -0.15 |
| **HEALTH-49 (*N* = 1,829) – (2) Depressiveness** | | | | | | | |
| **Games-Howell post-hoc test** | | | | | | | |
|  | Established Milieu (*N* = 144) | Liberal Intellectual Milieu | 0.11 | 0.09 | 0.955 | -0.26 | 0.48 |
|  |  | Performer Milieu | -0.24 | 0.13 | 0.639 | -0.79 | 0.31 |
|  |  | Cosmopolitan Avant-garde Milieu | -0.20 | 0.11 | 0.680 | -0.67 | 0.26 |
|  |  | Adaptive Navigator Milieu | -0.55* | 0.09 | **< 0.001** | -0.95 | -0.15 |
|  |  | Social Ecological Milieu | -0.29 | 0.08 | 0.007 | -0.63 | 0.04 |
|  |  | Modern Mainstreamer Milieu | -0.44* | 0.09 | **< 0.001** | -0.84 | -0.04 |
|  |  | Traditional Milieu | -0.67* | 0.11 | **< 0.001** | -1.16 | -0.18 |
|  |  | Precarious Milieu | -1.18* | 0.09 | **< 0.001** | -1.56 | -0.80 |
|  |  | Hedonist Milieu | -0.66* | 0.09 | **< 0.001** | -1.04 | -0.29 |
|  | Liberal Intellectual Milieu (*N* = 157) | Established Milieu | -0.11 | 0.09 | 0.955 | -0.48 | 0.26 |
|  |  | Performer Milieu | -0.36 | 0.12 | 0.106 | -0.89 | 0.18 |
|  |  | Cosmopolitan Avant-garde Milieu | -0.32 | 0.10 | 0.074 | -0.76 | 0.13 |
|  |  | Adaptive Navigator Milieu | -0.66* | 0.09 | **< 0.001** | -1.03 | -0.29 |
|  |  | Social Ecological Milieu | -0.41* | 0.07 | **< 0.001** | -0.70 | -0.11 |
|  |  | Modern Mainstreamer Milieu | -0.56* | 0.09 | **< 0.001** | -0.93 | -0.18 |
|  |  | Traditional Milieu | -0.78* | 0.11 | **< 0.001** | -1.25 | -0.32 |
|  |  | Precarious Milieu | -1.29* | 0.08 | **< 0.001** | -1.65 | -0.94 |
|  |  | Hedonist Milieu | -0.78* | 0.08 | **< 0.001** | -1.12 | -0.43 |
|  | Performer Milieu (*N* = 76) | Established Milieu | 0.24 | 0.13 | 0.639 | -0.31 | 0.79 |
|  |  | Liberal Intellectual Milieu | 0.36 | 0.12 | 0.106 | -0.18 | 0.89 |
|  |  | Cosmopolitan Avant-garde Milieu | 0.04 | 0.14 | 1.000 | -0.56 | 0.64 |
|  |  | Adaptive Navigator Milieu | -0.30 | 0.13 | 0.327 | -0.85 | 0.25 |
|  |  | Social Ecological Milieu | -0.05 | 0.11 | 1.000 | -0.56 | 0.46 |
|  |  | Modern Mainstreamer Milieu | -0.20 | 0.13 | 0.859 | -0.75 | 0.35 |
|  |  | Traditional Milieu | -0.43 | 0.14 | 0.083 | -1.04 | 0.19 |
|  |  | Precarious Milieu | -0.94* | 0.12 | **< 0.001** | -1.47 | -0.40 |
|  |  | Hedonist Milieu | -0.42 | 0.12 | 0.026 | -0.95 | 0.12 |
|  | Cosmopolitan Avant-garde Milieu (*N* = 97) | Established Milieu | 0.20 | 0.11 | 0.680 | -0.26 | 0.67 |
|  |  | Liberal Intellectual Milieu | 0.32 | 0.10 | 0.074 | -0.13 | 0.76 |
|  |  | Performer Milieu | -0.04 | 0.14 | 1.000 | -0.64 | 0.56 |
|  |  | Adaptive Navigator Milieu | -0.34 | 0.11 | 0.055 | -0.81 | 0.13 |
|  |  | Social Ecological Milieu | -0.09 | 0.10 | 0.995 | -0.50 | 0.33 |
|  |  | Modern Mainstreamer Milieu | -0.24 | 0.11 | 0.467 | -0.71 | 0.23 |
|  |  | Traditional Milieu | -0.47 | 0.13 | 0.010 | -1.01 | 0.08 |
|  |  | Precarious Milieu | -0.98* | 0.10 | **< 0.001** | -1.43 | -0.52 |
|  |  | Hedonist Milieu | -0.46* | 0.10 | **0.001** | -0.91 | -0.01 |
|  | Adaptive Navigator Milieu (*N* = 182) | Established Milieu | 0.55* | 0.09 | **< 0.001** | 0.15 | 0.95 |
|  |  | Liberal Intellectual Milieu | 0.66* | 0.09 | **< 0.001** | 0.29 | 1.03 |
|  |  | Performer Milieu | 0.30 | 0.13 | 0.327 | -0.25 | 0.85 |
|  |  | Cosmopolitan Avant-garde Milieu | 0.34 | 0.11 | 0.055 | -0.13 | 0.81 |
|  |  | Social Ecological Milieu | 0.25 | 0.08 | 0.036 | -0.08 | 0.59 |
|  |  | Modern Mainstreamer Milieu | 0.10 | 0.09 | 0.983 | -0.30 | 0.51 |
|  |  | Traditional Milieu | -0.12 | 0.11 | 0.985 | -0.61 | 0.36 |
|  |  | Precarious Milieu | -0.63* | 0.09 | **< 0.001** | -1.01 | -0.26 |
|  |  | Hedonist Milieu | -0.12 | 0.09 | 0.948 | -0.49 | 0.26 |
|  | Social Ecological Milieu (*N* = 410) | Established Milieu | 0.29 | 0.08 | 0.007 | -0.04 | 0.63 |
|  |  | Liberal Intellectual Milieu | 0.41* | 0.07 | **< 0.001** | 0.11 | 0.70 |
|  |  | Performer Milieu | 0.05 | 0.11 | 1.000 | -0.46 | 0.56 |
|  |  | Cosmopolitan Avant-garde Milieu | 0.09 | 0.10 | 0.995 | -0.33 | 0.50 |
|  |  | Adaptive Navigator Milieu | -0.25 | 0.08 | 0.036 | -0.59 | 0.08 |
|  |  | Modern Mainstreamer Milieu | -0.15 | 0.08 | 0.657 | -0.48 | 0.18 |
|  |  | Traditional Milieu | -0.38 | 0.10 | 0.009 | -0.81 | 0.06 |
|  |  | Precarious Milieu | -0.89* | 0.07 | **< 0.001** | -1.20 | -0.58 |
|  |  | Hedonist Milieu | -0.37* | 0.07 | **< 0.001** | -0.67 | -0.07 |
|  | Modern Mainstreamer Milieu (*N* = 195) | Established Milieu | 0.44* | 0.09 | **< 0.001** | 0.04 | 0.84 |
|  |  | Liberal Intellectual Milieu | 0.56* | 0.09 | **< 0.001** | 0.18 | 0.93 |
|  |  | Performer Milieu | 0.20 | 0.13 | 0.859 | -0.35 | 0.75 |
|  |  | Cosmopolitan Avant-garde Milieu | 0.24 | 0.11 | 0.467 | -0.23 | 0.71 |
|  |  | Adaptive Navigator Milieu | -0.10 | 0.09 | 0.983 | -0.51 | 0.30 |
|  |  | Social Ecological Milieu | 0.15 | 0.08 | 0.657 | -0.18 | 0.48 |
|  |  | Traditional Milieu | -0.23 | 0.11 | 0.593 | -0.72 | 0.26 |
|  |  | Precarious Milieu | -0.74* | 0.09 | **< 0.001** | -1.12 | -0.36 |
|  |  | Hedonist Milieu | -0.22 | 0.09 | 0.272 | -0.60 | 0.16 |
|  | Traditional Milieu (*N* = 98) | Established Milieu | 0.67* | 0.11 | **< 0.001** | 0.18 | 1.16 |
|  |  | Liberal Intellectual Milieu | 0.78* | 0.11 | **< 0.001** | 0.32 | 1.25 |
|  |  | Performer Milieu | 0.43 | 0.14 | 0.083 | -0.19 | 1.04 |
|  |  | Cosmopolitan Avant-garde Milieu | 0.47 | 0.13 | 0.010 | -0.08 | 1.01 |
|  |  | Adaptive Navigator Milieu | 0.12 | 0.11 | 0.985 | -0.36 | 0.61 |
|  |  | Social Ecological Milieu | 0.38 | 0.10 | 0.009 | -0.06 | 0.81 |
|  |  | Modern Mainstreamer Milieu | 0.23 | 0.11 | 0.593 | -0.26 | 0.72 |
|  |  | Precarious Milieu | -0.51* | 0.11 | **< 0.001** | -0.98 | -0.04 |
|  |  | Hedonist Milieu | 0.01 | 0.11 | 1.000 | -0.46 | 0.48 |
|  | Precarious Milieu (*N* = 229) | Established Milieu | 1.18* | 0.09 | **< 0.001** | 0.80 | 1.56 |
|  |  | Liberal Intellectual Milieu | 1.29* | 0.08 | **< 0.001** | 0.94 | 1.65 |
|  |  | Performer Milieu | 0.94* | 0.12 | **< 0.001** | 0.40 | 1.47 |
|  |  | Cosmopolitan Avant-garde Milieu | 0.98* | 0.10 | **< 0.001** | 0.52 | 1.43 |
|  |  | Adaptive Navigator Milieu | 0.63* | 0.09 | **< 0.001** | 0.26 | 1.01 |
|  |  | Social Ecological Milieu | 0.89* | 0.07 | **< 0.001** | 0.58 | 1.20 |
|  |  | Modern Mainstreamer Milieu | 0.74* | 0.09 | **< 0.001** | 0.36 | 1.12 |
|  |  | Traditional Milieu | 0.51* | 0.11 | **< 0.001** | 0.04 | 0.98 |
|  |  | Hedonist Milieu | 0.52* | 0.08 | **< 0.001** | 0.16 | 0.87 |
|  | Hedonist Milieu (*N* = 241) | Established Milieu | 0.66* | 0.09 | **< 0.001** | 0.29 | 1.04 |
|  |  | Liberal Intellectual Milieu | 0.78* | 0.08 | **< 0.001** | 0.43 | 1.12 |
|  |  | Performer Milieu | 0.42 | 0.12 | 0.026 | -0.12 | 0.95 |
|  |  | Cosmopolitan Avant-garde Milieu | 0.46* | 0.10 | **0.001** | 0.01 | 0.91 |
|  |  | Adaptive Navigator Milieu | 0.12 | 0.09 | 0.948 | -0.26 | 0.49 |
|  |  | Social Ecological Milieu | 0.37* | 0.07 | **< 0.001** | 0.07 | 0.67 |
|  |  | Modern Mainstreamer Milieu | 0.22 | 0.09 | 0.272 | -0.16 | 0.60 |
|  |  | Traditional Milieu | -0.01 | 0.11 | 1.000 | -0.48 | 0.46 |
|  |  | Precarious Milieu | -0.52* | 0.08 | **< 0.001** | -0.87 | -0.16 |
| **HEALTH-49 (*N* = 1,829) – (3) Phobic anxiety** | | | | | | | |
| **Games-Howell post-hoc test** | | | | | | | |
|  | Established Milieu (*N* = 144) | Liberal Intellectual Milieu | 0.01 | 0.08 | 1.000 | -0.33 | 0.36 |
|  |  | Performer Milieu | -0.15 | 0.11 | 0.935 | -0.62 | 0.33 |
|  |  | Cosmopolitan Avant-garde Milieu | 0.02 | 0.09 | 1.000 | -0.36 | 0.40 |
|  |  | Adaptive Navigator Milieu | -0.41* | 0.09 | **< 0.001** | -0.79 | -0.04 |
|  |  | Social Ecological Milieu | -0.23 | 0.07 | 0.035 | -0.53 | 0.07 |
|  |  | Modern Mainstreamer Milieu | -0.39* | 0.09 | **< 0.001** | -0.76 | -0.03 |
|  |  | Traditional Milieu | -0.66* | 0.12 | **< 0.001** | -1.17 | -0.16 |
|  |  | Precarious Milieu | -1.03* | 0.09 | **< 0.001** | -1.42 | -0.64 |
|  |  | Hedonist Milieu | -0.45* | 0.08 | **< 0.001** | -0.79 | -0.11 |
|  | Liberal Intellectual Milieu (*N* = 157) | Established Milieu | -0.01 | 0.08 | 1.000 | -0.36 | 0.33 |
|  |  | Performer Milieu | -0.16 | 0.11 | 0.891 | -0.64 | 0.31 |
|  |  | Cosmopolitan Avant-garde Milieu | 0.00 | 0.09 | 1.000 | -0.38 | 0.38 |
|  |  | Adaptive Navigator Milieu | -0.43* | 0.09 | **< 0.001** | -0.81 | -0.05 |
|  |  | Social Ecological Milieu | -0.25 | 0.07 | 0.018 | -0.55 | 0.05 |
|  |  | Modern Mainstreamer Milieu | -0.41* | 0.09 | **< 0.001** | -0.78 | -0.04 |
|  |  | Traditional Milieu | -0.68* | 0.12 | **< 0.001** | -1.18 | -0.17 |
|  |  | Precarious Milieu | -1.04* | 0.09 | **< 0.001** | -1.43 | -0.65 |
|  |  | Hedonist Milieu | -0.46* | 0.08 | **< 0.001** | -0.80 | -0.12 |
|  | Performer Milieu (*N* = 76) | Established Milieu | 0.15 | 0.11 | 0.935 | -0.33 | 0.62 |
|  |  | Liberal Intellectual Milieu | 0.16 | 0.11 | 0.891 | -0.31 | 0.64 |
|  |  | Cosmopolitan Avant-garde Milieu | 0.17 | 0.12 | 0.913 | -0.34 | 0.67 |
|  |  | Adaptive Navigator Milieu | -0.27 | 0.12 | 0.387 | -0.77 | 0.23 |
|  |  | Social Ecological Milieu | -0.08 | 0.10 | 0.998 | -0.53 | 0.37 |
|  |  | Modern Mainstreamer Milieu | -0.25 | 0.11 | 0.486 | -0.74 | 0.25 |
|  |  | Traditional Milieu | -0.51 | 0.14 | 0.009 | -1.11 | 0.08 |
|  |  | Precarious Milieu | -0.88* | 0.12 | **< 0.001** | -1.39 | -0.37 |
|  |  | Hedonist Milieu | -0.30 | 0.11 | 0.166 | -0.78 | 0.18 |
|  | Cosmopolitan Avant-garde Milieu (*N* = 97) | Established Milieu | -0.02 | 0.09 | 1.000 | -0.40 | 0.36 |
|  |  | Liberal Intellectual Milieu | 0.00 | 0.09 | 1.000 | -0.38 | 0.38 |
|  |  | Performer Milieu | -0.17 | 0.12 | 0.913 | -0.67 | 0.34 |
|  |  | Adaptive Navigator Milieu | -0.43* | 0.10 | **< 0.001** | -0.84 | -0.02 |
|  |  | Social Ecological Milieu | -0.25 | 0.08 | 0.063 | -0.59 | 0.10 |
|  |  | Modern Mainstreamer Milieu | -0.41* | 0.09 | **0.001** | -0.81 | -0.01 |
|  |  | Traditional Milieu | -0.68* | 0.12 | **< 0.001** | -1.21 | -0.15 |
|  |  | Precarious Milieu | -1.05* | 0.10 | **< 0.001** | -1.47 | -0.62 |
|  |  | Hedonist Milieu | -0.47* | 0.09 | **< 0.001** | -0.85 | -0.08 |
|  | Adaptive Navigator Milieu (*N* = 182) | Established Milieu | 0.41* | 0.09 | **< 0.001** | 0.04 | 0.79 |
|  |  | Liberal Intellectual Milieu | 0.43* | 0.09 | **< 0.001** | 0.05 | 0.81 |
|  |  | Performer Milieu | 0.27 | 0.12 | 0.387 | -0.23 | 0.77 |
|  |  | Cosmopolitan Avant-garde Milieu | 0.43* | 0.10 | **< 0.001** | 0.02 | 0.84 |
|  |  | Social Ecological Milieu | 0.18 | 0.08 | 0.401 | -0.16 | 0.53 |
|  |  | Modern Mainstreamer Milieu | 0.02 | 0.09 | 1.000 | -0.38 | 0.42 |
|  |  | Traditional Milieu | -0.25 | 0.12 | 0.576 | -0.78 | 0.28 |
|  |  | Precarious Milieu | -0.61* | 0.10 | **< 0.001** | -1.03 | -0.19 |
|  |  | Hedonist Milieu | -0.03 | 0.09 | 1.000 | -0.41 | 0.35 |
|  | Social Ecological Milieu (*N* = 410) | Established Milieu | 0.23 | 0.07 | 0.035 | -0.07 | 0.53 |
|  |  | Liberal Intellectual Milieu | 0.25 | 0.07 | 0.018 | -0.05 | 0.55 |
|  |  | Performer Milieu | 0.08 | 0.10 | 0.998 | -0.37 | 0.53 |
|  |  | Cosmopolitan Avant-garde Milieu | 0.25 | 0.08 | 0.063 | -0.10 | 0.59 |
|  |  | Adaptive Navigator Milieu | -0.18 | 0.08 | 0.401 | -0.53 | 0.16 |
|  |  | Modern Mainstreamer Milieu | -0.16 | 0.08 | 0.535 | -0.49 | 0.17 |
|  |  | Traditional Milieu | -0.43 | 0.11 | 0.005 | -0.91 | 0.05 |
|  |  | Precarious Milieu | -0.80* | 0.08 | **< 0.001** | -1.15 | -0.44 |
|  |  | Hedonist Milieu | -0.22 | 0.07 | 0.072 | -0.52 | 0.09 |
|  | Modern Mainstreamer Milieu (*N* = 195) | Established Milieu | 0.39* | 0.09 | **< 0.001** | 0.03 | 0.76 |
|  |  | Liberal Intellectual Milieu | 0.41* | 0.09 | **< 0.001** | 0.04 | 0.78 |
|  |  | Performer Milieu | 0.25 | 0.11 | 0.486 | -0.25 | 0.74 |
|  |  | Cosmopolitan Avant-garde Milieu | 0.41* | 0.09 | **0.001** | 0.01 | 0.81 |
|  |  | Adaptive Navigator Milieu | -0.02 | 0.09 | 1.000 | -0.42 | 0.38 |
|  |  | Social Ecological Milieu | 0.16 | 0.08 | 0.535 | -0.17 | 0.49 |
|  |  | Traditional Milieu | -0.27 | 0.12 | 0.436 | -0.79 | 0.25 |
|  |  | Precarious Milieu | -0.63* | 0.10 | **< 0.001** | -1.05 | -0.22 |
|  |  | Hedonist Milieu | -0.05 | 0.09 | 1.000 | -0.42 | 0.31 |
|  | Traditional Milieu (*N* = 98) | Established Milieu | 0.66* | 0.12 | **< 0.001** | 0.16 | 1.17 |
|  |  | Liberal Intellectual Milieu | 0.68* | 0.12 | **< 0.001** | 0.17 | 1.18 |
|  |  | Performer Milieu | 0.51 | 0.14 | 0.009 | -0.08 | 1.11 |
|  |  | Cosmopolitan Avant-garde Milieu | 0.68* | 0.12 | **< 0.001** | 0.15 | 1.21 |
|  |  | Adaptive Navigator Milieu | 0.25 | 0.12 | 0.576 | -0.28 | 0.78 |
|  |  | Social Ecological Milieu | 0.43 | 0.11 | 0.005 | -0.05 | 0.91 |
|  |  | Modern Mainstreamer Milieu | 0.27 | 0.12 | 0.436 | -0.25 | 0.79 |
|  |  | Precarious Milieu | -0.36 | 0.12 | 0.101 | -0.90 | 0.17 |
|  |  | Hedonist Milieu | 0.22 | 0.12 | 0.701 | -0.29 | 0.72 |
|  | Precarious Milieu (*N* = 229) | Established Milieu | 1.03* | 0.09 | **< 0.001** | 0.64 | 1.42 |
|  |  | Liberal Intellectual Milieu | 1.04* | 0.09 | **< 0.001** | 0.65 | 1.43 |
|  |  | Performer Milieu | 0.88* | 0.12 | **< 0.001** | 0.37 | 1.39 |
|  |  | Cosmopolitan Avant-garde Milieu | 1.05* | 0.10 | **< 0.001** | 0.62 | 1.47 |
|  |  | Adaptive Navigator Milieu | 0.61* | 0.10 | **< 0.001** | 0.19 | 1.03 |
|  |  | Social Ecological Milieu | 0.80* | 0.08 | **< 0.001** | 0.44 | 1.15 |
|  |  | Modern Mainstreamer Milieu | 0.63* | 0.10 | **< 0.001** | 0.22 | 1.05 |
|  |  | Traditional Milieu | 0.36 | 0.12 | 0.101 | -0.17 | 0.90 |
|  |  | Hedonist Milieu | 0.58* | 0.09 | **< 0.001** | 0.19 | 0.97 |
|  | Hedonist Milieu (*N* = 241) | Established Milieu | 0.45* | 0.08 | **< 0.001** | 0.11 | 0.79 |
|  |  | Liberal Intellectual Milieu | 0.46* | 0.08 | **< 0.001** | 0.12 | 0.80 |
|  |  | Performer Milieu | 0.30 | 0.11 | 0.166 | -0.18 | 0.78 |
|  |  | Cosmopolitan Avant-garde Milieu | 0.47* | 0.09 | **< 0.001** | 0.08 | 0.85 |
|  |  | Adaptive Navigator Milieu | 0.03 | 0.09 | 1.000 | -0.35 | 0.41 |
|  |  | Social Ecological Milieu | 0.22 | 0.07 | 0.072 | -0.09 | 0.52 |
|  |  | Modern Mainstreamer Milieu | 0.05 | 0.09 | 1.000 | -0.31 | 0.42 |
|  |  | Traditional Milieu | -0.22 | 0.12 | 0.701 | -0.72 | 0.29 |
|  |  | Precarious Milieu | -0.58* | 0.09 | **< 0.001** | -0.97 | -0.19 |
| **HEALTH-49 (*N* = 1,829) – (4) Psychological and somatoform complaints** | | | | | | | |
| **Games-Howell post-hoc test** | | | | | | | |
|  | Established Milieu (*N* = 144) | Liberal Intellectual Milieu | -0.02 | 0.08 | 1.000 | -0.35 | 0.30 |
|  |  | Performer Milieu | -0.21 | 0.10 | 0.510 | -0.64 | 0.22 |
|  |  | Cosmopolitan Avant-garde Milieu | -0.19 | 0.08 | 0.428 | -0.55 | 0.17 |
|  |  | Adaptive Navigator Milieu | -0.56* | 0.08 | **< 0.001** | -0.89 | -0.23 |
|  |  | Social Ecological Milieu | -0.38* | 0.06 | **< 0.001** | -0.66 | -0.11 |
|  |  | Modern Mainstreamer Milieu | -0.50* | 0.08 | **< 0.001** | -0.84 | -0.16 |
|  |  | Traditional Milieu | -0.66* | 0.10 | **< 0.001** | -1.07 | -0.24 |
|  |  | Precarious Milieu | -1.11* | 0.08 | **< 0.001** | -1.43 | -0.78 |
|  |  | Hedonist Milieu | -0.61* | 0.07 | **< 0.001** | -0.91 | -0.31 |
|  | Liberal Intellectual Milieu (*N* = 157) | Established Milieu | 0.02 | 0.08 | 1.000 | -0.30 | 0.35 |
|  |  | Performer Milieu | -0.19 | 0.10 | 0.669 | -0.62 | 0.24 |
|  |  | Cosmopolitan Avant-garde Milieu | -0.16 | 0.08 | 0.614 | -0.52 | 0.19 |
|  |  | Adaptive Navigator Milieu | -0.54* | 0.08 | **< 0.001** | -0.87 | -0.21 |
|  |  | Social Ecological Milieu | -0.36* | 0.06 | **< 0.001** | -0.63 | -0.09 |
|  |  | Modern Mainstreamer Milieu | -0.47* | 0.08 | **< 0.001** | -0.81 | -0.14 |
|  |  | Traditional Milieu | -0.63* | 0.10 | **< 0.001** | -1.04 | -0.22 |
|  |  | Precarious Milieu | -1.08* | 0.07 | **< 0.001** | -1.40 | -0.76 |
|  |  | Hedonist Milieu | -0.59* | 0.07 | **< 0.001** | -0.88 | -0.29 |
|  | Performer Milieu (*N* = 76) | Established Milieu | 0.21 | 0.10 | 0.510 | -0.22 | 0.64 |
|  |  | Liberal Intellectual Milieu | 0.19 | 0.10 | 0.669 | -0.24 | 0.62 |
|  |  | Cosmopolitan Avant-garde Milieu | 0.02 | 0.10 | 1.000 | -0.43 | 0.48 |
|  |  | Adaptive Navigator Milieu | -0.35 | 0.10 | 0.022 | -0.79 | 0.09 |
|  |  | Social Ecological Milieu | -0.17 | 0.09 | 0.671 | -0.57 | 0.23 |
|  |  | Modern Mainstreamer Milieu | -0.29 | 0.10 | 0.137 | -0.73 | 0.16 |
|  |  | Traditional Milieu | -0.45 | 0.11 | 0.006 | -0.94 | 0.05 |
|  |  | Precarious Milieu | -0.89* | 0.10 | **< 0.001** | -1.32 | -0.46 |
|  |  | Hedonist Milieu | -0.40 | 0.09 | 0.002 | -0.81 | 0.01 |
|  | Cosmopolitan Avant-garde Milieu (*N* = 97) | Established Milieu | 0.19 | 0.08 | 0.428 | -0.17 | 0.55 |
|  |  | Liberal Intellectual Milieu | 0.16 | 0.08 | 0.614 | -0.19 | 0.52 |
|  |  | Performer Milieu | -0.02 | 0.10 | 1.000 | -0.48 | 0.43 |
|  |  | Adaptive Navigator Milieu | -0.37* | 0.09 | **0.001** | -0.74 | 0.00 |
|  |  | Social Ecological Milieu | -0.19 | 0.07 | 0.202 | -0.51 | 0.12 |
|  |  | Modern Mainstreamer Milieu | -0.31 | 0.09 | 0.015 | -0.68 | 0.06 |
|  |  | Traditional Milieu | -0.47* | 0.10 | **< 0.001** | -0.91 | -0.03 |
|  |  | Precarious Milieu | -0.92* | 0.08 | **< 0.001** | -1.28 | -0.56 |
|  |  | Hedonist Milieu | -0.42* | 0.08 | **< 0.001** | -0.76 | -0.08 |
|  | Adaptive Navigator Milieu (*N* = 182) | Established Milieu | 0.56* | 0.08 | **< 0.001** | 0.23 | 0.89 |
|  |  | Liberal Intellectual Milieu | 0.54* | 0.08 | **< 0.001** | 0.21 | 0.87 |
|  |  | Performer Milieu | 0.35 | 0.10 | 0.022 | -0.09 | 0.79 |
|  |  | Cosmopolitan Avant-garde Milieu | 0.37* | 0.09 | **0.001** | 0.00 | 0.74 |
|  |  | Social Ecological Milieu | 0.18 | 0.07 | 0.180 | -0.11 | 0.46 |
|  |  | Modern Mainstreamer Milieu | 0.06 | 0.08 | 0.999 | -0.29 | 0.41 |
|  |  | Traditional Milieu | -0.10 | 0.10 | 0.992 | -0.52 | 0.32 |
|  |  | Precarious Milieu | -0.55* | 0.08 | **< 0.001** | -0.88 | -0.21 |
|  |  | Hedonist Milieu | -0.05 | 0.07 | 0.999 | -0.36 | 0.26 |
|  | Social Ecological Milieu (*N* = 410) | Established Milieu | 0.38* | 0.06 | **< 0.001** | 0.11 | 0.66 |
|  |  | Liberal Intellectual Milieu | 0.36* | 0.06 | **< 0.001** | 0.09 | 0.63 |
|  |  | Performer Milieu | 0.17 | 0.09 | 0.671 | -0.23 | 0.57 |
|  |  | Cosmopolitan Avant-garde Milieu | 0.19 | 0.07 | 0.202 | -0.12 | 0.51 |
|  |  | Adaptive Navigator Milieu | -0.18 | 0.07 | 0.180 | -0.46 | 0.11 |
|  |  | Modern Mainstreamer Milieu | -0.12 | 0.07 | 0.790 | -0.41 | 0.18 |
|  |  | Traditional Milieu | -0.28 | 0.09 | 0.055 | -0.65 | 0.10 |
|  |  | Precarious Milieu | -0.72* | 0.06 | **< 0.001** | -0.99 | -0.45 |
|  |  | Hedonist Milieu | -0.23 | 0.06 | 0.003 | -0.47 | 0.01 |
|  | Modern Mainstreamer Milieu (*N* = 195) | Established Milieu | 0.50* | 0.08 | **< 0.001** | 0.16 | 0.84 |
|  |  | Liberal Intellectual Milieu | 0.47* | 0.08 | **< 0.001** | 0.14 | 0.81 |
|  |  | Performer Milieu | 0.29 | 0.10 | 0.137 | -0.16 | 0.73 |
|  |  | Cosmopolitan Avant-garde Milieu | 0.31 | 0.09 | 0.015 | -0.06 | 0.68 |
|  |  | Adaptive Navigator Milieu | -0.06 | 0.08 | 0.999 | -0.41 | 0.29 |
|  |  | Social Ecological Milieu | 0.12 | 0.07 | 0.790 | -0.18 | 0.41 |
|  |  | Traditional Milieu | -0.16 | 0.10 | 0.842 | -0.58 | 0.27 |
|  |  | Precarious Milieu | -0.61* | 0.08 | **< 0.001** | -0.94 | -0.27 |
|  |  | Hedonist Milieu | -0.11 | 0.07 | 0.881 | -0.43 | 0.20 |
|  | Traditional Milieu (*N* = 98) | Established Milieu | 0.66* | 0.10 | **< 0.001** | 0.24 | 1.07 |
|  |  | Liberal Intellectual Milieu | 0.63* | 0.10 | **< 0.001** | 0.22 | 1.04 |
|  |  | Performer Milieu | 0.45 | 0.11 | 0.006 | -0.05 | 0.94 |
|  |  | Cosmopolitan Avant-garde Milieu | 0.47* | 0.10 | **< 0.001** | 0.03 | 0.91 |
|  |  | Adaptive Navigator Milieu | 0.10 | 0.10 | 0.992 | -0.32 | 0.52 |
|  |  | Social Ecological Milieu | 0.28 | 0.09 | 0.055 | -0.10 | 0.65 |
|  |  | Modern Mainstreamer Milieu | 0.16 | 0.10 | 0.842 | -0.27 | 0.58 |
|  |  | Precarious Milieu | -0.45* | 0.10 | **< 0.001** | -0.86 | -0.04 |
|  |  | Hedonist Milieu | 0.05 | 0.09 | 1.000 | -0.35 | 0.44 |
|  | Precarious Milieu (*N* = 229) | Established Milieu | 1.11* | 0.08 | **< 0.001** | 0.78 | 1.43 |
|  |  | Liberal Intellectual Milieu | 1.08* | 0.07 | **< 0.001** | 0.76 | 1.40 |
|  |  | Performer Milieu | 0.89* | 0.10 | **< 0.001** | 0.46 | 1.32 |
|  |  | Cosmopolitan Avant-garde Milieu | 0.92* | 0.08 | **< 0.001** | 0.56 | 1.28 |
|  |  | Adaptive Navigator Milieu | 0.55* | 0.08 | **< 0.001** | 0.21 | 0.88 |
|  |  | Social Ecological Milieu | 0.72* | 0.06 | **< 0.001** | 0.45 | 0.99 |
|  |  | Modern Mainstreamer Milieu | 0.61* | 0.08 | **< 0.001** | 0.27 | 0.94 |
|  |  | Traditional Milieu | 0.45* | 0.10 | **< 0.001** | 0.04 | 0.86 |
|  |  | Hedonist Milieu | 0.49* | 0.07 | **< 0.001** | 0.20 | 0.79 |
|  | Hedonist Milieu (*N* = 241) | Established Milieu | 0.61* | 0.07 | **< 0.001** | 0.31 | 0.91 |
|  |  | Liberal Intellectual Milieu | 0.59* | 0.07 | **< 0.001** | 0.29 | 0.88 |
|  |  | Performer Milieu | 0.40 | 0.09 | 0.002 | -0.01 | 0.81 |
|  |  | Cosmopolitan Avant-garde Milieu | 0.42* | 0.08 | **< 0.001** | 0.08 | 0.76 |
|  |  | Adaptive Navigator Milieu | 0.05 | 0.07 | 0.999 | -0.26 | 0.36 |
|  |  | Social Ecological Milieu | 0.23 | 0.06 | 0.003 | -0.01 | 0.47 |
|  |  | Modern Mainstreamer Milieu | 0.11 | 0.07 | 0.881 | -0.20 | 0.43 |
|  |  | Traditional Milieu | -0.05 | 0.09 | 1.000 | -0.44 | 0.35 |
|  |  | Precarious Milieu | -0.49* | 0.07 | **< 0.001** | -0.79 | -0.20 |
| **HEALTH-49 (*N* = 1,829) – (5) Psychological well-being** | | | | | | | |
| **Games-Howell post-hoc test** | | | | | | | |
|  | Established Milieu (*N* = 144) | Liberal Intellectual Milieu | -0.02 | 0.08 | 1.000 | -0.37 | 0.33 |
|  |  | Performer Milieu | -0.20 | 0.10 | 0.601 | -0.63 | 0.23 |
|  |  | Cosmopolitan Avant-garde Milieu | -0.20 | 0.09 | 0.390 | -0.57 | 0.17 |
|  |  | Adaptive Navigator Milieu | -0.49* | 0.08 | **< 0.001** | -0.83 | -0.15 |
|  |  | Social Ecological Milieu | -0.34* | 0.07 | **< 0.001** | -0.63 | -0.05 |
|  |  | Modern Mainstreamer Milieu | -0.41* | 0.08 | **< 0.001** | -0.75 | -0.06 |
|  |  | Traditional Milieu | -0.64* | 0.09 | **< 0.001** | -1.05 | -0.24 |
|  |  | Precarious Milieu | -0.89* | 0.07 | **< 0.001** | -1.20 | -0.58 |
|  |  | Hedonist Milieu | -0.47* | 0.07 | **< 0.001** | -0.78 | -0.16 |
|  | Liberal Intellectual Milieu (*N* = 157) | Established Milieu | 0.02 | 0.08 | 1.000 | -0.33 | 0.37 |
|  |  | Performer Milieu | -0.18 | 0.10 | 0.713 | -0.61 | 0.25 |
|  |  | Cosmopolitan Avant-garde Milieu | -0.18 | 0.09 | 0.516 | -0.55 | 0.19 |
|  |  | Adaptive Navigator Milieu | -0.47* | 0.08 | **< 0.001** | -0.81 | -0.14 |
|  |  | Social Ecological Milieu | -0.32* | 0.07 | **< 0.001** | -0.61 | -0.04 |
|  |  | Modern Mainstreamer Milieu | -0.39* | 0.08 | **< 0.001** | -0.73 | -0.05 |
|  |  | Traditional Milieu | -0.63* | 0.09 | **< 0.001** | -1.03 | -0.22 |
|  |  | Precarious Milieu | -0.87* | 0.07 | **< 0.001** | -1.18 | -0.57 |
|  |  | Hedonist Milieu | -0.45* | 0.07 | **< 0.001** | -0.75 | -0.14 |
|  | Performer Milieu (*N* = 76) | Established Milieu | 0.20 | 0.10 | 0.601 | -0.23 | 0.63 |
|  |  | Liberal Intellectual Milieu | 0.18 | 0.10 | 0.713 | -0.25 | 0.61 |
|  |  | Cosmopolitan Avant-garde Milieu | 0.00 | 0.10 | 1.000 | -0.44 | 0.44 |
|  |  | Adaptive Navigator Milieu | -0.29 | 0.10 | 0.076 | -0.71 | 0.12 |
|  |  | Social Ecological Milieu | -0.14 | 0.09 | 0.821 | -0.52 | 0.24 |
|  |  | Modern Mainstreamer Milieu | -0.21 | 0.10 | 0.489 | -0.63 | 0.21 |
|  |  | Traditional Milieu | -0.45 | 0.11 | 0.003 | -0.92 | 0.03 |
|  |  | Precarious Milieu | -0.69* | 0.09 | **< 0.001** | -1.09 | -0.30 |
|  |  | Hedonist Milieu | -0.27 | 0.09 | 0.094 | -0.67 | 0.13 |
|  | Cosmopolitan Avant-garde Milieu (*N* = 97) | Established Milieu | 0.20 | 0.09 | 0.390 | -0.17 | 0.57 |
|  |  | Liberal Intellectual Milieu | 0.18 | 0.09 | 0.516 | -0.19 | 0.55 |
|  |  | Performer Milieu | 0.00 | 0.10 | 1.000 | -0.44 | 0.44 |
|  |  | Adaptive Navigator Milieu | -0.29 | 0.08 | 0.017 | -0.65 | 0.06 |
|  |  | Social Ecological Milieu | -0.14 | 0.07 | 0.611 | -0.45 | 0.17 |
|  |  | Modern Mainstreamer Milieu | -0.21 | 0.08 | 0.280 | -0.57 | 0.15 |
|  |  | Traditional Milieu | -0.45* | 0.10 | **< 0.001** | -0.87 | -0.02 |
|  |  | Precarious Milieu | -0.69* | 0.08 | **< 0.001** | -1.02 | -0.36 |
|  |  | Hedonist Milieu | -0.27 | 0.08 | 0.018 | -0.60 | 0.06 |
|  | Adaptive Navigator Milieu (*N* = 182) | Established Milieu | 0.49* | 0.08 | **< 0.001** | 0.15 | 0.83 |
|  |  | Liberal Intellectual Milieu | 0.47* | 0.08 | **< 0.001** | 0.14 | 0.81 |
|  |  | Performer Milieu | 0.29 | 0.10 | 0.076 | -0.12 | 0.71 |
|  |  | Cosmopolitan Avant-garde Milieu | 0.29 | 0.08 | 0.017 | -0.06 | 0.65 |
|  |  | Social Ecological Milieu | 0.15 | 0.06 | 0.322 | -0.12 | 0.42 |
|  |  | Modern Mainstreamer Milieu | 0.08 | 0.08 | 0.985 | -0.24 | 0.41 |
|  |  | Traditional Milieu | -0.15 | 0.09 | 0.808 | -0.55 | 0.24 |
|  |  | Precarious Milieu | -0.40* | 0.07 | **< 0.001** | -0.69 | -0.11 |
|  |  | Hedonist Milieu | 0.02 | 0.07 | 1.000 | -0.27 | 0.32 |
|  | Social Ecological Milieu (*N* = 410) | Established Milieu | 0.34* | 0.07 | **< 0.001** | 0.05 | 0.63 |
|  |  | Liberal Intellectual Milieu | 0.32* | 0.07 | **< 0.001** | 0.04 | 0.61 |
|  |  | Performer Milieu | 0.14 | 0.09 | 0.821 | -0.24 | 0.52 |
|  |  | Cosmopolitan Avant-garde Milieu | 0.14 | 0.07 | 0.611 | -0.17 | 0.45 |
|  |  | Adaptive Navigator Milieu | -0.15 | 0.06 | 0.322 | -0.42 | 0.12 |
|  |  | Modern Mainstreamer Milieu | -0.07 | 0.06 | 0.989 | -0.34 | 0.21 |
|  |  | Traditional Milieu | -0.31 | 0.08 | 0.010 | -0.66 | 0.05 |
|  |  | Precarious Milieu | -0.55* | 0.05 | **< 0.001** | -0.78 | -0.32 |
|  |  | Hedonist Milieu | -0.13 | 0.05 | 0.349 | -0.36 | 0.10 |
|  | Modern Mainstreamer Milieu (*N* = 195) | Established Milieu | 0.41* | 0.08 | **< 0.001** | 0.06 | 0.75 |
|  |  | Liberal Intellectual Milieu | 0.39* | 0.08 | **< 0.001** | 0.05 | 0.73 |
|  |  | Performer Milieu | 0.21 | 0.10 | 0.489 | -0.21 | 0.63 |
|  |  | Cosmopolitan Avant-garde Milieu | 0.21 | 0.08 | 0.280 | -0.15 | 0.57 |
|  |  | Adaptive Navigator Milieu | -0.08 | 0.08 | 0.985 | -0.41 | 0.24 |
|  |  | Social Ecological Milieu | 0.07 | 0.06 | 0.989 | -0.21 | 0.34 |
|  |  | Traditional Milieu | -0.24 | 0.09 | 0.244 | -0.64 | 0.16 |
|  |  | Precarious Milieu | -0.48* | 0.07 | **< 0.001** | -0.78 | -0.18 |
|  |  | Hedonist Milieu | -0.06 | 0.07 | 0.997 | -0.36 | 0.24 |
|  | Traditional Milieu (*N* = 98) | Established Milieu | 0.64* | 0.09 | **< 0.001** | 0.24 | 1.05 |
|  |  | Liberal Intellectual Milieu | 0.63* | 0.09 | **< 0.001** | 0.22 | 1.03 |
|  |  | Performer Milieu | 0.45 | 0.11 | 0.003 | -0.03 | 0.92 |
|  |  | Cosmopolitan Avant-garde Milieu | 0.45* | 0.10 | **< 0.001** | 0.02 | 0.87 |
|  |  | Adaptive Navigator Milieu | 0.15 | 0.09 | 0.808 | -0.24 | 0.55 |
|  |  | Social Ecological Milieu | 0.31 | 0.08 | 0.010 | -0.05 | 0.66 |
|  |  | Modern Mainstreamer Milieu | 0.24 | 0.09 | 0.244 | -0.16 | 0.64 |
|  |  | Precarious Milieu | -0.24 | 0.09 | 0.127 | -0.62 | 0.13 |
|  |  | Hedonist Milieu | 0.18 | 0.09 | 0.549 | -0.19 | 0.55 |
|  | Precarious Milieu (*N* = 229) | Established Milieu | 0.89* | 0.07 | **< 0.001** | 0.58 | 1.20 |
|  |  | Liberal Intellectual Milieu | 0.87* | 0.07 | **< 0.001** | 0.57 | 1.18 |
|  |  | Performer Milieu | 0.69* | 0.09 | **< 0.001** | 0.30 | 1.09 |
|  |  | Cosmopolitan Avant-garde Milieu | 0.69* | 0.08 | **< 0.001** | 0.36 | 1.02 |
|  |  | Adaptive Navigator Milieu | 0.40* | 0.07 | **< 0.001** | 0.11 | 0.69 |
|  |  | Social Ecological Milieu | 0.55* | 0.05 | **< 0.001** | 0.32 | 0.78 |
|  |  | Modern Mainstreamer Milieu | 0.48* | 0.07 | **< 0.001** | 0.18 | 0.78 |
|  |  | Traditional Milieu | 0.24 | 0.09 | 0.127 | -0.13 | 0.62 |
|  |  | Hedonist Milieu | 0.42* | 0.06 | **< 0.001** | 0.17 | 0.68 |
|  | Hedonist Milieu (*N* = 241) | Established Milieu | 0.47* | 0.07 | **< 0.001** | 0.16 | 0.78 |
|  |  | Liberal Intellectual Milieu | 0.45* | 0.07 | **< 0.001** | 0.14 | 0.75 |
|  |  | Performer Milieu | 0.27 | 0.09 | 0.094 | -0.13 | 0.67 |
|  |  | Cosmopolitan Avant-garde Milieu | 0.27 | 0.08 | 0.018 | -0.06 | 0.60 |
|  |  | Adaptive Navigator Milieu | -0.02 | 0.07 | 1.000 | -0.32 | 0.27 |
|  |  | Social Ecological Milieu | 0.13 | 0.05 | 0.349 | -0.10 | 0.36 |
|  |  | Modern Mainstreamer Milieu | 0.06 | 0.07 | 0.997 | -0.24 | 0.36 |
|  |  | Traditional Milieu | -0.18 | 0.09 | 0.549 | -0.55 | 0.19 |
|  |  | Precarious Milieu | -0.42* | 0.06 | **< 0.001** | -0.68 | -0.17 |
| **HEALTH-49 (*N* = 1,829) – (6) Interactional difficulties** | | | | | | | |
| **Games-Howell post-hoc test** | | | | | | | |
|  | Established Milieu (*N* = 144) | Liberal Intellectual Milieu | 0.08 | 0.10 | 0.998 | -0.34 | 0.50 |
|  |  | Performer Milieu | -0.18 | 0.12 | 0.866 | -0.68 | 0.32 |
|  |  | Cosmopolitan Avant-garde Milieu | -0.08 | 0.10 | 0.998 | -0.52 | 0.36 |
|  |  | Adaptive Navigator Milieu | -0.50* | 0.09 | **< 0.001** | -0.91 | -0.10 |
|  |  | Social Ecological Milieu | -0.19 | 0.08 | 0.359 | -0.53 | 0.15 |
|  |  | Modern Mainstreamer Milieu | -0.43* | 0.09 | **< 0.001** | -0.82 | -0.04 |
|  |  | Traditional Milieu | -0.58* | 0.11 | **< 0.001** | -1.06 | -0.11 |
|  |  | Precarious Milieu | -0.83* | 0.09 | **< 0.001** | -1.22 | -0.45 |
|  |  | Hedonist Milieu | -0.47* | 0.09 | **< 0.001** | -0.83 | -0.10 |
|  | Liberal Intellectual Milieu (*N* = 157) | Established Milieu | -0.08 | 0.10 | 0.998 | -0.50 | 0.34 |
|  |  | Performer Milieu | -0.26 | 0.12 | 0.444 | -0.76 | 0.25 |
|  |  | Cosmopolitan Avant-garde Milieu | -0.16 | 0.10 | 0.862 | -0.60 | 0.28 |
|  |  | Adaptive Navigator Milieu | -0.58* | 0.10 | **< 0.001** | -0.99 | -0.17 |
|  |  | Social Ecological Milieu | -0.27 | 0.08 | 0.038 | -0.62 | 0.08 |
|  |  | Modern Mainstreamer Milieu | -0.51* | 0.09 | **< 0.001** | -0.91 | -0.12 |
|  |  | Traditional Milieu | -0.66* | 0.11 | **< 0.001** | -1.14 | -0.18 |
|  |  | Precarious Milieu | -0.91* | 0.09 | **< 0.001** | -1.30 | -0.52 |
|  |  | Hedonist Milieu | -0.55* | 0.09 | **< 0.001** | -0.92 | -0.17 |
|  | Performer Milieu (*N* = 76) | Established Milieu | 0.18 | 0.12 | 0.866 | -0.32 | 0.68 |
|  |  | Liberal Intellectual Milieu | 0.26 | 0.12 | 0.444 | -0.25 | 0.76 |
|  |  | Cosmopolitan Avant-garde Milieu | 0.10 | 0.12 | 0.998 | -0.42 | 0.62 |
|  |  | Adaptive Navigator Milieu | -0.32 | 0.11 | 0.129 | -0.82 | 0.17 |
|  |  | Social Ecological Milieu | -0.01 | 0.10 | 1.000 | -0.45 | 0.44 |
|  |  | Modern Mainstreamer Milieu | -0.25 | 0.11 | 0.406 | -0.73 | 0.23 |
|  |  | Traditional Milieu | -0.41 | 0.13 | 0.050 | -0.95 | 0.14 |
|  |  | Precarious Milieu | -0.65* | 0.11 | **< 0.001** | -1.13 | -0.18 |
|  |  | Hedonist Milieu | -0.29 | 0.11 | 0.187 | -0.75 | 0.18 |
|  | Cosmopolitan Avant-garde Milieu (*N* = 97) | Established Milieu | 0.08 | 0.10 | 0.998 | -0.36 | 0.52 |
|  |  | Liberal Intellectual Milieu | 0.16 | 0.10 | 0.862 | -0.28 | 0.60 |
|  |  | Performer Milieu | -0.10 | 0.12 | 0.998 | -0.62 | 0.42 |
|  |  | Adaptive Navigator Milieu | -0.42* | 0.10 | **0.001** | -0.85 | 0.01 |
|  |  | Social Ecological Milieu | -0.11 | 0.09 | 0.965 | -0.48 | 0.26 |
|  |  | Modern Mainstreamer Milieu | -0.35 | 0.10 | 0.012 | -0.76 | 0.06 |
|  |  | Traditional Milieu | -0.50* | 0.11 | **0.001** | -1.00 | -0.01 |
|  |  | Precarious Milieu | -0.75* | 0.09 | **< 0.001** | -1.16 | -0.34 |
|  |  | Hedonist Milieu | -0.38 | 0.09 | 0.002 | -0.78 | 0.01 |
|  | Adaptive Navigator Milieu (*N* = 182) | Established Milieu | 0.50* | 0.09 | **< 0.001** | 0.10 | 0.91 |
|  |  | Liberal Intellectual Milieu | 0.58* | 0.10 | **< 0.001** | 0.17 | 0.99 |
|  |  | Performer Milieu | 0.32 | 0.11 | 0.129 | -0.17 | 0.82 |
|  |  | Cosmopolitan Avant-garde Milieu | 0.42* | 0.10 | **0.001** | -0.01 | 0.85 |
|  |  | Social Ecological Milieu | 0.32 | 0.08 | 0.002 | -0.01 | 0.64 |
|  |  | Modern Mainstreamer Milieu | 0.07 | 0.09 | 0.999 | -0.31 | 0.45 |
|  |  | Traditional Milieu | -0.08 | 0.11 | 0.999 | -0.55 | 0.38 |
|  |  | Precarious Milieu | -0.33 | 0.09 | 0.007 | -0.70 | 0.04 |
|  |  | Hedonist Milieu | 0.04 | 0.08 | 1.000 | -0.32 | 0.39 |
|  | Social Ecological Milieu (*N* = 410) | Established Milieu | 0.19 | 0.08 | 0.359 | -0.15 | 0.53 |
|  |  | Liberal Intellectual Milieu | 0.27 | 0.08 | 0.038 | -0.08 | 0.62 |
|  |  | Performer Milieu | 0.01 | 0.10 | 1.000 | -0.44 | 0.45 |
|  |  | Cosmopolitan Avant-garde Milieu | 0.11 | 0.09 | 0.965 | -0.26 | 0.48 |
|  |  | Adaptive Navigator Milieu | -0.32 | 0.08 | 0.002 | -0.64 | 0.01 |
|  |  | Modern Mainstreamer Milieu | -0.24 | 0.07 | 0.028 | -0.55 | 0.07 |
|  |  | Traditional Milieu | -0.40 | 0.10 | 0.002 | -0.81 | 0.02 |
|  |  | Precarious Milieu | -0.65* | 0.07 | **< 0.001** | -0.95 | -0.34 |
|  |  | Hedonist Milieu | -0.28* | 0.07 | **0.001** | -0.56 | 0.00 |
|  | Modern Mainstreamer Milieu (*N* = 195) | Established Milieu | 0.43* | 0.09 | **< 0.001** | 0.04 | 0.82 |
|  |  | Liberal Intellectual Milieu | 0.51* | 0.09 | **< 0.001** | 0.12 | 0.91 |
|  |  | Performer Milieu | 0.25 | 0.11 | 0.406 | -0.23 | 0.73 |
|  |  | Cosmopolitan Avant-garde Milieu | 0.35 | 0.10 | 0.012 | -0.06 | 0.76 |
|  |  | Adaptive Navigator Milieu | -0.07 | 0.09 | 0.999 | -0.45 | 0.31 |
|  |  | Social Ecological Milieu | 0.24 | 0.07 | 0.028 | -0.07 | 0.55 |
|  |  | Traditional Milieu | -0.15 | 0.10 | 0.906 | -0.61 | 0.30 |
|  |  | Precarious Milieu | -0.40* | 0.08 | **< 0.001** | -0.76 | -0.04 |
|  |  | Hedonist Milieu | -0.03 | 0.08 | 1.000 | -0.37 | 0.30 |
|  | Traditional Milieu (*N* = 98) | Established Milieu | 0.58* | 0.11 | **< 0.001** | 0.11 | 1.06 |
|  |  | Liberal Intellectual Milieu | 0.66* | 0.11 | **< 0.001** | 0.18 | 1.14 |
|  |  | Performer Milieu | 0.41 | 0.13 | 0.050 | -0.14 | 0.95 |
|  |  | Cosmopolitan Avant-garde Milieu | 0.50* | 0.11 | **0.001** | 0.01 | 1.00 |
|  |  | Adaptive Navigator Milieu | 0.08 | 0.11 | 0.999 | -0.38 | 0.55 |
|  |  | Social Ecological Milieu | 0.40 | 0.10 | 0.002 | -0.02 | 0.81 |
|  |  | Modern Mainstreamer Milieu | 0.15 | 0.10 | 0.906 | -0.30 | 0.61 |
|  |  | Precarious Milieu | -0.25 | 0.10 | 0.342 | -0.70 | 0.20 |
|  |  | Hedonist Milieu | 0.12 | 0.10 | 0.974 | -0.32 | 0.56 |
|  | Precarious Milieu (*N* = 229) | Established Milieu | 0.83* | 0.09 | **< 0.001** | 0.45 | 1.22 |
|  |  | Liberal Intellectual Milieu | 0.91* | 0.09 | **< 0.001** | 0.52 | 1.30 |
|  |  | Performer Milieu | 0.65* | 0.11 | **< 0.001** | 0.18 | 1.13 |
|  |  | Cosmopolitan Avant-garde Milieu | 0.75* | 0.09 | **< 0.001** | 0.34 | 1.16 |
|  |  | Adaptive Navigator Milieu | 0.33 | 0.09 | 0.007 | -0.04 | 0.70 |
|  |  | Social Ecological Milieu | 0.65* | 0.07 | **< 0.001** | 0.34 | 0.95 |
|  |  | Modern Mainstreamer Milieu | 0.40* | 0.08 | **< 0.001** | 0.04 | 0.76 |
|  |  | Traditional Milieu | 0.25 | 0.10 | 0.342 | -0.20 | 0.70 |
|  |  | Hedonist Milieu | 0.37* | 0.08 | **< 0.001** | 0.03 | 0.70 |
|  | Hedonist Milieu (*N* = 241) | Established Milieu | 0.47* | 0.09 | **< 0.001** | 0.10 | 0.83 |
|  |  | Liberal Intellectual Milieu | 0.55* | 0.09 | **< 0.001** | 0.17 | 0.92 |
|  |  | Performer Milieu | 0.29 | 0.11 | 0.187 | -0.18 | 0.75 |
|  |  | Cosmopolitan Avant-garde Milieu | 0.38 | 0.09 | 0.002 | -0.01 | 0.78 |
|  |  | Adaptive Navigator Milieu | -0.04 | 0.08 | 1.000 | -0.39 | 0.32 |
|  |  | Social Ecological Milieu | 0.28* | 0.07 | **0.001** | 0.00 | 0.56 |
|  |  | Modern Mainstreamer Milieu | 0.03 | 0.08 | 1.000 | -0.30 | 0.37 |
|  |  | Traditional Milieu | -0.12 | 0.10 | 0.974 | -0.56 | 0.32 |
|  |  | Precarious Milieu | -0.37* | 0.08 | **< 0.001** | -0.70 | -0.03 |
| **HEALTH-49 (*N* = 1,829) – (7) Self-efficacy** | | | | | | | |
| **Games-Howell post-hoc test** | | | | | | | |
|  | Established Milieu (*N* = 144) | Liberal Intellectual Milieu | 0.14 | 0.09 | 0.854 | -0.24 | 0.52 |
|  |  | Performer Milieu | -0.21 | 0.11 | 0.714 | -0.70 | 0.28 |
|  |  | Cosmopolitan Avant-garde Milieu | -0.08 | 0.10 | 0.997 | -0.51 | 0.34 |
|  |  | Adaptive Navigator Milieu | -0.45* | 0.08 | **< 0.001** | -0.82 | -0.09 |
|  |  | Social Ecological Milieu | -0.38* | 0.07 | **< 0.001** | -0.70 | -0.06 |
|  |  | Modern Mainstreamer Milieu | -0.34 | 0.09 | 0.004 | -0.71 | 0.03 |
|  |  | Traditional Milieu | -0.76* | 0.11 | **< 0.001** | -1.25 | -0.28 |
|  |  | Precarious Milieu | -0.99* | 0.08 | **< 0.001** | -1.32 | -0.66 |
|  |  | Hedonist Milieu | -0.57* | 0.08 | **< 0.001** | -0.91 | -0.23 |
|  | Liberal Intellectual Milieu (*N* = 157) | Established Milieu | -0.14 | 0.09 | 0.854 | -0.52 | 0.24 |
|  |  | Performer Milieu | -0.35 | 0.11 | 0.068 | -0.83 | 0.14 |
|  |  | Cosmopolitan Avant-garde Milieu | -0.22 | 0.10 | 0.376 | -0.64 | 0.19 |
|  |  | Adaptive Navigator Milieu | -0.59* | 0.08 | **< 0.001** | -0.95 | -0.24 |
|  |  | Social Ecological Milieu | -0.52* | 0.07 | **< 0.001** | -0.83 | -0.21 |
|  |  | Modern Mainstreamer Milieu | -0.48* | 0.08 | **< 0.001** | -0.84 | -0.12 |
|  |  | Traditional Milieu | -0.90* | 0.11 | **< 0.001** | -1.38 | -0.42 |
|  |  | Precarious Milieu | -1.13* | 0.07 | **< 0.001** | -1.45 | -0.80 |
|  |  | Hedonist Milieu | -0.71* | 0.08 | **< 0.001** | -1.04 | -0.38 |
|  | Performer Milieu (*N* = 76) | Established Milieu | 0.21 | 0.11 | 0.714 | -0.28 | 0.70 |
|  |  | Liberal Intellectual Milieu | 0.35 | 0.11 | 0.068 | -0.14 | 0.83 |
|  |  | Cosmopolitan Avant-garde Milieu | 0.12 | 0.12 | 0.990 | -0.40 | 0.64 |
|  |  | Adaptive Navigator Milieu | -0.25 | 0.11 | 0.427 | -0.72 | 0.23 |
|  |  | Social Ecological Milieu | -0.17 | 0.10 | 0.783 | -0.62 | 0.27 |
|  |  | Modern Mainstreamer Milieu | -0.14 | 0.11 | 0.966 | -0.62 | 0.35 |
|  |  | Traditional Milieu | -0.56 | 0.13 | 0.002 | -1.13 | 0.02 |
|  |  | Precarious Milieu | -0.78* | 0.10 | **< 0.001** | -1.23 | -0.33 |
|  |  | Hedonist Milieu | -0.36 | 0.10 | 0.024 | -0.82 | 0.10 |
|  | Cosmopolitan Avant-garde Milieu (*N* = 97) | Established Milieu | 0.08 | 0.10 | 0.997 | -0.34 | 0.51 |
|  |  | Liberal Intellectual Milieu | 0.22 | 0.10 | 0.376 | -0.19 | 0.64 |
|  |  | Performer Milieu | -0.12 | 0.12 | 0.990 | -0.64 | 0.40 |
|  |  | Adaptive Navigator Milieu | -0.37 | 0.09 | 0.004 | -0.77 | 0.04 |
|  |  | Social Ecological Milieu | -0.29 | 0.08 | 0.019 | -0.66 | 0.07 |
|  |  | Modern Mainstreamer Milieu | -0.26 | 0.10 | 0.176 | -0.67 | 0.15 |
|  |  | Traditional Milieu | -0.68* | 0.12 | **< 0.001** | -1.19 | -0.16 |
|  |  | Precarious Milieu | -0.90* | 0.09 | **< 0.001** | -1.28 | -0.53 |
|  |  | Hedonist Milieu | -0.48* | 0.09 | **< 0.001** | -0.87 | -0.10 |
|  | Adaptive Navigator Milieu (*N* = 182) | Established Milieu | 0.45* | 0.08 | **< 0.001** | 0.09 | 0.82 |
|  |  | Liberal Intellectual Milieu | 0.59* | 0.08 | **< 0.001** | 0.24 | 0.95 |
|  |  | Performer Milieu | 0.25 | 0.11 | 0.427 | -0.23 | 0.72 |
|  |  | Cosmopolitan Avant-garde Milieu | 0.37 | 0.09 | 0.004 | -0.04 | 0.77 |
|  |  | Social Ecological Milieu | 0.07 | 0.07 | 0.987 | -0.22 | 0.36 |
|  |  | Modern Mainstreamer Milieu | 0.11 | 0.08 | 0.943 | -0.24 | 0.46 |
|  |  | Traditional Milieu | -0.31 | 0.11 | 0.127 | -0.78 | 0.16 |
|  |  | Precarious Milieu | -0.53* | 0.07 | **< 0.001** | -0.84 | -0.23 |
|  |  | Hedonist Milieu | -0.12 | 0.07 | 0.847 | -0.43 | 0.20 |
|  | Social Ecological Milieu (*N* = 410) | Established Milieu | 0.38* | 0.07 | **< 0.001** | 0.06 | 0.70 |
|  |  | Liberal Intellectual Milieu | 0.52* | 0.07 | **< 0.001** | 0.21 | 0.83 |
|  |  | Performer Milieu | 0.17 | 0.10 | 0.783 | -0.27 | 0.62 |
|  |  | Cosmopolitan Avant-garde Milieu | 0.29 | 0.08 | 0.019 | -0.07 | 0.66 |
|  |  | Adaptive Navigator Milieu | -0.07 | 0.07 | 0.987 | -0.36 | 0.22 |
|  |  | Modern Mainstreamer Milieu | 0.04 | 0.07 | 1.000 | -0.26 | 0.34 |
|  |  | Traditional Milieu | -0.38 | 0.10 | 0.008 | -0.82 | 0.06 |
|  |  | Precarious Milieu | -0.61* | 0.06 | **< 0.001** | -0.85 | -0.36 |
|  |  | Hedonist Milieu | -0.19 | 0.06 | 0.053 | -0.45 | 0.07 |
|  | Modern Mainstreamer Milieu (*N* = 195) | Established Milieu | 0.34 | 0.09 | 0.004 | -0.03 | 0.71 |
|  |  | Liberal Intellectual Milieu | 0.48* | 0.08 | **< 0.001** | 0.12 | 0.84 |
|  |  | Performer Milieu | 0.14 | 0.11 | 0.966 | -0.35 | 0.62 |
|  |  | Cosmopolitan Avant-garde Milieu | 0.26 | 0.10 | 0.176 | -0.15 | 0.67 |
|  |  | Adaptive Navigator Milieu | -0.11 | 0.08 | 0.943 | -0.46 | 0.24 |
|  |  | Social Ecological Milieu | -0.04 | 0.07 | 1.000 | -0.34 | 0.26 |
|  |  | Traditional Milieu | -0.42 | 0.11 | 0.007 | -0.90 | 0.06 |
|  |  | Precarious Milieu | -0.64* | 0.07 | **< 0.001** | -0.96 | -0.33 |
|  |  | Hedonist Milieu | -0.23 | 0.08 | 0.080 | -0.55 | 0.09 |
|  | Traditional Milieu (*N* = 98) | Established Milieu | 0.76* | 0.11 | **< 0.001** | 0.28 | 1.25 |
|  |  | Liberal Intellectual Milieu | 0.90* | 0.11 | **< 0.001** | 0.42 | 1.38 |
|  |  | Performer Milieu | 0.56 | 0.13 | 0.002 | -0.02 | 1.13 |
|  |  | Cosmopolitan Avant-garde Milieu | 0.68* | 0.12 | **< 0.001** | 0.16 | 1.19 |
|  |  | Adaptive Navigator Milieu | 0.31 | 0.11 | 0.127 | -0.16 | 0.78 |
|  |  | Social Ecological Milieu | 0.38 | 0.10 | 0.008 | -0.06 | 0.82 |
|  |  | Modern Mainstreamer Milieu | 0.42 | 0.11 | 0.007 | -0.06 | 0.90 |
|  |  | Precarious Milieu | -0.22 | 0.10 | 0.476 | -0.67 | 0.22 |
|  |  | Hedonist Milieu | 0.19 | 0.10 | 0.698 | -0.26 | 0.65 |
|  | Precarious Milieu (*N* = 229) | Established Milieu | 0.99* | 0.08 | **< 0.001** | 0.66 | 1.32 |
|  |  | Liberal Intellectual Milieu | 1.13* | 0.07 | **< 0.001** | 0.80 | 1.45 |
|  |  | Performer Milieu | 0.78* | 0.10 | **< 0.001** | 0.33 | 1.23 |
|  |  | Cosmopolitan Avant-garde Milieu | 0.90* | 0.09 | **< 0.001** | 0.53 | 1.28 |
|  |  | Adaptive Navigator Milieu | 0.53* | 0.07 | **< 0.001** | 0.23 | 0.84 |
|  |  | Social Ecological Milieu | 0.61* | 0.06 | **< 0.001** | 0.36 | 0.85 |
|  |  | Modern Mainstreamer Milieu | 0.64* | 0.07 | **< 0.001** | 0.33 | 0.96 |
|  |  | Traditional Milieu | 0.22 | 0.10 | 0.476 | -0.22 | 0.67 |
|  |  | Hedonist Milieu | 0.42* | 0.06 | **< 0.001** | 0.14 | 0.69 |
|  | Hedonist Milieu (*N* = 241) | Established Milieu | 0.57* | 0.08 | **< 0.001** | 0.23 | 0.91 |
|  |  | Liberal Intellectual Milieu | 0.71* | 0.08 | **< 0.001** | 0.38 | 1.04 |
|  |  | Performer Milieu | 0.36 | 0.10 | 0.024 | -0.10 | 0.82 |
|  |  | Cosmopolitan Avant-garde Milieu | 0.48* | 0.09 | **< 0.001** | 0.10 | 0.87 |
|  |  | Adaptive Navigator Milieu | 0.12 | 0.07 | 0.847 | -0.20 | 0.43 |
|  |  | Social Ecological Milieu | 0.19 | 0.06 | 0.053 | -0.07 | 0.45 |
|  |  | Modern Mainstreamer Milieu | 0.23 | 0.08 | 0.080 | -0.09 | 0.55 |
|  |  | Traditional Milieu | -0.19 | 0.10 | 0.698 | -0.65 | 0.26 |
|  |  | Precarious Milieu | -0.42* | 0.06 | **< 0.001** | -0.69 | -0.14 |
| **HEALTH-49 (*N* = 1,829) – (8) Activity and Participation** | | | | | | | |
| **Tukey post-hoc test** | | | | | | | |
|  | Established Milieu (*N* = 144) | Liberal Intellectual Milieu | 0.13 | 0.09 | 0.908 | -0.24 | 0.49 |
|  |  | Performer Milieu | -0.07 | 0.11 | 1.000 | -0.52 | 0.38 |
|  |  | Cosmopolitan Avant-garde Milieu | -0.18 | 0.10 | 0.708 | -0.60 | 0.23 |
|  |  | Adaptive Navigator Milieu | -0.35* | 0.08 | **0.001** | -0.70 | 0.00 |
|  |  | Social Ecological Milieu | -0.29 | 0.07 | 0.002 | -0.60 | 0.01 |
|  |  | Modern Mainstreamer Milieu | -0.36* | 0.08 | **0.001** | -0.71 | -0.01 |
|  |  | Traditional Milieu | -0.47* | 0.10 | **< 0.001** | -0.88 | -0.05 |
|  |  | Precarious Milieu | -0.69* | 0.08 | **< 0.001** | -1.03 | -0.35 |
|  |  | Hedonist Milieu | -0.43* | 0.08 | **< 0.001** | -0.76 | -0.10 |
|  | Liberal Intellectual Milieu (*N* = 157) | Established Milieu | -0.13 | 0.09 | 0.908 | -0.49 | 0.24 |
|  |  | Performer Milieu | -0.19 | 0.10 | 0.701 | -0.63 | 0.25 |
|  |  | Cosmopolitan Avant-garde Milieu | -0.31 | 0.10 | 0.049 | -0.71 | 0.10 |
|  |  | Adaptive Navigator Milieu | -0.47* | 0.08 | **< 0.001** | -0.82 | -0.13 |
|  |  | Social Ecological Milieu | -0.42* | 0.07 | **< 0.001** | -0.71 | -0.12 |
|  |  | Modern Mainstreamer Milieu | -0.48* | 0.08 | **< 0.001** | -0.82 | -0.15 |
|  |  | Traditional Milieu | -0.59* | 0.10 | **< 0.001** | -1.00 | -0.19 |
|  |  | Precarious Milieu | -0.82* | 0.08 | **< 0.001** | -1.14 | -0.49 |
|  |  | Hedonist Milieu | -0.56* | 0.08 | **< 0.001** | -0.88 | -0.23 |
|  | Performer Milieu (*N* = 76) | Established Milieu | 0.07 | 0.11 | 1.000 | -0.38 | 0.52 |
|  |  | Liberal Intellectual Milieu | 0.19 | 0.10 | 0.701 | -0.25 | 0.63 |
|  |  | Cosmopolitan Avant-garde Milieu | -0.11 | 0.11 | 0.993 | -0.60 | 0.37 |
|  |  | Adaptive Navigator Milieu | -0.28 | 0.10 | 0.151 | -0.71 | 0.15 |
|  |  | Social Ecological Milieu | -0.22 | 0.09 | 0.324 | -0.62 | 0.17 |
|  |  | Modern Mainstreamer Milieu | -0.29 | 0.10 | 0.110 | -0.72 | 0.14 |
|  |  | Traditional Milieu | -0.40 | 0.11 | 0.016 | -0.88 | 0.08 |
|  |  | Precarious Milieu | -0.62* | 0.10 | **< 0.001** | -1.04 | -0.21 |
|  |  | Hedonist Milieu | -0.36 | 0.10 | 0.008 | -0.78 | 0.05 |
|  | Cosmopolitan Avant-garde Milieu (*N* = 97) | Established Milieu | 0.18 | 0.10 | 0.708 | -0.23 | 0.60 |
|  |  | Liberal Intellectual Milieu | 0.31 | 0.10 | 0.049 | -0.10 | 0.71 |
|  |  | Performer Milieu | 0.11 | 0.11 | 0.993 | -0.37 | 0.60 |
|  |  | Adaptive Navigator Milieu | -0.17 | 0.09 | 0.739 | -0.57 | 0.23 |
|  |  | Social Ecological Milieu | -0.11 | 0.08 | 0.949 | -0.47 | 0.25 |
|  |  | Modern Mainstreamer Milieu | -0.18 | 0.09 | 0.653 | -0.57 | 0.21 |
|  |  | Traditional Milieu | -0.29 | 0.11 | 0.177 | -0.74 | 0.16 |
|  |  | Precarious Milieu | -0.51* | 0.09 | **< 0.001** | -0.89 | -0.13 |
|  |  | Hedonist Milieu | -0.25 | 0.09 | 0.139 | -0.63 | 0.13 |
|  | Adaptive Navigator Milieu (*N* = 182) | Established Milieu | 0.35* | 0.08 | **0.001** | 0.00 | 0.70 |
|  |  | Liberal Intellectual Milieu | 0.47* | 0.08 | **< 0.001** | 0.13 | 0.82 |
|  |  | Performer Milieu | 0.28 | 0.10 | 0.151 | -0.15 | 0.71 |
|  |  | Cosmopolitan Avant-garde Milieu | 0.17 | 0.09 | 0.739 | -0.23 | 0.57 |
|  |  | Social Ecological Milieu | 0.06 | 0.07 | 0.997 | -0.22 | 0.34 |
|  |  | Modern Mainstreamer Milieu | -0.01 | 0.08 | 1.000 | -0.34 | 0.32 |
|  |  | Traditional Milieu | -0.12 | 0.09 | 0.958 | -0.52 | 0.28 |
|  |  | Precarious Milieu | -0.34* | 0.07 | **< 0.001** | -0.66 | -0.03 |
|  |  | Hedonist Milieu | -0.08 | 0.07 | 0.982 | -0.39 | 0.23 |
|  | Social Ecological Milieu (*N* = 410) | Established Milieu | 0.29 | 0.07 | 0.002 | -0.01 | 0.60 |
|  |  | Liberal Intellectual Milieu | 0.42* | 0.07 | **< 0.001** | 0.12 | 0.71 |
|  |  | Performer Milieu | 0.22 | 0.09 | 0.324 | -0.17 | 0.62 |
|  |  | Cosmopolitan Avant-garde Milieu | 0.11 | 0.08 | 0.949 | -0.25 | 0.47 |
|  |  | Adaptive Navigator Milieu | -0.06 | 0.07 | 0.997 | -0.34 | 0.22 |
|  |  | Modern Mainstreamer Milieu | -0.07 | 0.06 | 0.990 | -0.34 | 0.21 |
|  |  | Traditional Milieu | -0.18 | 0.08 | 0.522 | -0.53 | 0.18 |
|  |  | Precarious Milieu | -0.40* | 0.06 | **< 0.001** | -0.66 | -0.14 |
|  |  | Hedonist Milieu | -0.14 | 0.06 | 0.385 | -0.40 | 0.12 |
|  | Modern Mainstreamer Milieu (*N* = 195) | Established Milieu | 0.36* | 0.08 | **0.001** | 0.01 | 0.71 |
|  |  | Liberal Intellectual Milieu | 0.48* | 0.08 | **< 0.001** | 0.15 | 0.82 |
|  |  | Performer Milieu | 0.29 | 0.10 | 0.110 | -0.14 | 0.72 |
|  |  | Cosmopolitan Avant-garde Milieu | 0.18 | 0.09 | 0.653 | -0.21 | 0.57 |
|  |  | Adaptive Navigator Milieu | 0.01 | 0.08 | 1.000 | -0.32 | 0.34 |
|  |  | Social Ecological Milieu | 0.07 | 0.06 | 0.990 | -0.21 | 0.34 |
|  |  | Traditional Milieu | -0.11 | 0.09 | 0.975 | -0.50 | 0.28 |
|  |  | Precarious Milieu | -0.33* | 0.07 | **< 0.001** | -0.64 | -0.02 |
|  |  | Hedonist Milieu | -0.07 | 0.07 | 0.992 | -0.38 | 0.23 |
|  | Traditional Milieu (*N* = 98) | Established Milieu | 0.47* | 0.10 | **< 0.001** | 0.05 | 0.88 |
|  |  | Liberal Intellectual Milieu | 0.59* | 0.10 | **< 0.001** | 0.19 | 1.00 |
|  |  | Performer Milieu | 0.40 | 0.11 | 0.016 | -0.08 | 0.88 |
|  |  | Cosmopolitan Avant-garde Milieu | 0.29 | 0.11 | 0.177 | -0.16 | 0.74 |
|  |  | Adaptive Navigator Milieu | 0.12 | 0.09 | 0.958 | -0.28 | 0.52 |
|  |  | Social Ecological Milieu | 0.18 | 0.08 | 0.522 | -0.18 | 0.53 |
|  |  | Modern Mainstreamer Milieu | 0.11 | 0.09 | 0.975 | -0.28 | 0.50 |
|  |  | Precarious Milieu | -0.22 | 0.09 | 0.285 | -0.60 | 0.16 |
|  |  | Hedonist Milieu | 0.04 | 0.09 | 1.000 | -0.34 | 0.42 |
|  | Precarious Milieu (*N* = 229) | Established Milieu | 0.69* | 0.08 | **< 0.001** | 0.35 | 1.03 |
|  |  | Liberal Intellectual Milieu | 0.82* | 0.08 | **< 0.001** | 0.49 | 1.14 |
|  |  | Performer Milieu | 0.62* | 0.10 | **< 0.001** | 0.21 | 1.04 |
|  |  | Cosmopolitan Avant-garde Milieu | 0.51* | 0.09 | **< 0.001** | 0.13 | 0.89 |
|  |  | Adaptive Navigator Milieu | 0.34* | 0.07 | **< 0.001** | 0.03 | 0.66 |
|  |  | Social Ecological Milieu | 0.40* | 0.06 | **< 0.001** | 0.14 | 0.66 |
|  |  | Modern Mainstreamer Milieu | 0.33* | 0.07 | **< 0.001** | 0.02 | 0.64 |
|  |  | Traditional Milieu | 0.22 | 0.09 | 0.285 | -0.16 | 0.60 |
|  |  | Hedonist Milieu | 0.26 | 0.07 | 0.006 | -0.03 | 0.55 |
|  | Hedonist Milieu (*N* = 241) | Established Milieu | 0.43* | 0.08 | **< 0.001** | 0.10 | 0.76 |
|  |  | Liberal Intellectual Milieu | 0.56* | 0.08 | **< 0.001** | 0.23 | 0.88 |
|  |  | Performer Milieu | 0.36 | 0.10 | 0.008 | -0.05 | 0.78 |
|  |  | Cosmopolitan Avant-garde Milieu | 0.25 | 0.09 | 0.139 | -0.13 | 0.63 |
|  |  | Adaptive Navigator Milieu | 0.08 | 0.07 | 0.982 | -0.23 | 0.39 |
|  |  | Social Ecological Milieu | 0.14 | 0.06 | 0.385 | -0.12 | 0.40 |
|  |  | Modern Mainstreamer Milieu | 0.07 | 0.07 | 0.992 | -0.23 | 0.38 |
|  |  | Traditional Milieu | -0.04 | 0.09 | 1.000 | -0.42 | 0.34 |
|  |  | Precarious Milieu | -0.26 | 0.07 | 0.006 | -0.55 | 0.03 |
| **HEALTH-49 (*N* = 1,829) – (9) Social support** | | | | | | | |
| **Tukey post-hoc test** | | | | | | | |
|  | Established Milieu (*N* = 144) | Liberal Intellectual Milieu | 0.16 | 0.09 | 0.753 | -0.22 | 0.53 |
|  |  | Performer Milieu | -0.08 | 0.11 | 0.999 | -0.54 | 0.38 |
|  |  | Cosmopolitan Avant-garde Milieu | -0.10 | 0.10 | 0.994 | -0.52 | 0.33 |
|  |  | Adaptive Navigator Milieu | -0.16 | 0.09 | 0.675 | -0.52 | 0.20 |
|  |  | Social Ecological Milieu | 0.03 | 0.07 | 1.000 | -0.29 | 0.34 |
|  |  | Modern Mainstreamer Milieu | -0.17 | 0.08 | 0.623 | -0.52 | 0.19 |
|  |  | Traditional Milieu | -0.11 | 0.10 | 0.987 | -0.53 | 0.32 |
|  |  | Precarious Milieu | -0.36* | 0.08 | **< 0.001** | -0.71 | -0.02 |
|  |  | Hedonist Milieu | -0.32 | 0.08 | 0.004 | -0.66 | 0.03 |
|  | Liberal Intellectual Milieu (*N* = 157) | Established Milieu | -0.16 | 0.09 | 0.753 | -0.53 | 0.22 |
|  |  | Performer Milieu | -0.24 | 0.11 | 0.432 | -0.69 | 0.21 |
|  |  | Cosmopolitan Avant-garde Milieu | -0.25 | 0.10 | 0.237 | -0.67 | 0.17 |
|  |  | Adaptive Navigator Milieu | -0.32 | 0.08 | 0.006 | -0.67 | 0.04 |
|  |  | Social Ecological Milieu | -0.13 | 0.07 | 0.727 | -0.44 | 0.17 |
|  |  | Modern Mainstreamer Milieu | -0.32 | 0.08 | 0.004 | -0.67 | 0.03 |
|  |  | Traditional Milieu | -0.26 | 0.10 | 0.183 | -0.68 | 0.15 |
|  |  | Precarious Milieu | -0.52* | 0.08 | **< 0.001** | -0.86 | -0.18 |
|  |  | Hedonist Milieu | -0.47* | 0.08 | **< 0.001** | -0.81 | -0.14 |
|  | Performer Milieu (*N* = 76) | Established Milieu | 0.08 | 0.11 | 0.999 | -0.38 | 0.54 |
|  |  | Liberal Intellectual Milieu | 0.24 | 0.11 | 0.432 | -0.21 | 0.69 |
|  |  | Cosmopolitan Avant-garde Milieu | -0.01 | 0.12 | 1.000 | -0.51 | 0.48 |
|  |  | Adaptive Navigator Milieu | -0.08 | 0.10 | 0.999 | -0.52 | 0.36 |
|  |  | Social Ecological Milieu | 0.11 | 0.10 | 0.981 | -0.30 | 0.51 |
|  |  | Modern Mainstreamer Milieu | -0.08 | 0.10 | 0.999 | -0.52 | 0.36 |
|  |  | Traditional Milieu | -0.02 | 0.12 | 1.000 | -0.52 | 0.47 |
|  |  | Precarious Milieu | -0.28 | 0.10 | 0.153 | -0.71 | 0.15 |
|  |  | Hedonist Milieu | -0.23 | 0.10 | 0.377 | -0.66 | 0.19 |
|  | Cosmopolitan Avant-garde Milieu (*N* = 97) | Established Milieu | 0.10 | 0.10 | 0.994 | -0.33 | 0.52 |
|  |  | Liberal Intellectual Milieu | 0.25 | 0.10 | 0.237 | -0.17 | 0.67 |
|  |  | Performer Milieu | 0.01 | 0.12 | 1.000 | -0.48 | 0.51 |
|  |  | Adaptive Navigator Milieu | -0.06 | 0.10 | 1.000 | -0.47 | 0.34 |
|  |  | Social Ecological Milieu | 0.12 | 0.09 | 0.921 | -0.24 | 0.49 |
|  |  | Modern Mainstreamer Milieu | -0.07 | 0.10 | 0.999 | -0.47 | 0.33 |
|  |  | Traditional Milieu | -0.01 | 0.11 | 1.000 | -0.48 | 0.45 |
|  |  | Precarious Milieu | -0.27 | 0.09 | 0.118 | -0.66 | 0.13 |
|  |  | Hedonist Milieu | -0.22 | 0.09 | 0.335 | -0.61 | 0.17 |
|  | Adaptive Navigator Milieu (*N* = 182) | Established Milieu | 0.16 | 0.09 | 0.675 | -0.20 | 0.52 |
|  |  | Liberal Intellectual Milieu | 0.32 | 0.08 | 0.006 | -0.04 | 0.67 |
|  |  | Performer Milieu | 0.08 | 0.10 | 0.999 | -0.36 | 0.52 |
|  |  | Cosmopolitan Avant-garde Milieu | 0.06 | 0.10 | 1.000 | -0.34 | 0.47 |
|  |  | Social Ecological Milieu | 0.19 | 0.07 | 0.154 | -0.10 | 0.48 |
|  |  | Modern Mainstreamer Milieu | 0.00 | 0.08 | 1.000 | -0.34 | 0.33 |
|  |  | Traditional Milieu | 0.05 | 0.10 | 1.000 | -0.35 | 0.46 |
|  |  | Precarious Milieu | -0.20 | 0.08 | 0.202 | -0.52 | 0.12 |
|  |  | Hedonist Milieu | -0.16 | 0.08 | 0.557 | -0.47 | 0.16 |
|  | Social Ecological Milieu (*N* = 410) | Established Milieu | -0.03 | 0.07 | 1.000 | -0.34 | 0.29 |
|  |  | Liberal Intellectual Milieu | 0.13 | 0.07 | 0.727 | -0.17 | 0.44 |
|  |  | Performer Milieu | -0.11 | 0.10 | 0.981 | -0.51 | 0.30 |
|  |  | Cosmopolitan Avant-garde Milieu | -0.12 | 0.09 | 0.921 | -0.49 | 0.24 |
|  |  | Adaptive Navigator Milieu | -0.19 | 0.07 | 0.154 | -0.48 | 0.10 |
|  |  | Modern Mainstreamer Milieu | -0.19 | 0.07 | 0.113 | -0.47 | 0.09 |
|  |  | Traditional Milieu | -0.13 | 0.09 | 0.870 | -0.50 | 0.23 |
|  |  | Precarious Milieu | -0.39* | 0.06 | **< 0.001** | -0.66 | -0.12 |
|  |  | Hedonist Milieu | -0.34* | 0.06 | **< 0.001** | -0.61 | -0.08 |
|  | Modern Mainstreamer Milieu (*N* = 195) | Established Milieu | 0.17 | 0.08 | 0.623 | -0.19 | 0.52 |
|  |  | Liberal Intellectual Milieu | 0.32 | 0.08 | 0.004 | -0.03 | 0.67 |
|  |  | Performer Milieu | 0.08 | 0.10 | 0.999 | -0.36 | 0.52 |
|  |  | Cosmopolitan Avant-garde Milieu | 0.07 | 0.10 | 0.999 | -0.33 | 0.47 |
|  |  | Adaptive Navigator Milieu | 0.00 | 0.08 | 1.000 | -0.33 | 0.34 |
|  |  | Social Ecological Milieu | 0.19 | 0.07 | 0.113 | -0.09 | 0.47 |
|  |  | Traditional Milieu | 0.06 | 0.10 | 1.000 | -0.34 | 0.46 |
|  |  | Precarious Milieu | -0.20 | 0.07 | 0.203 | -0.51 | 0.12 |
|  |  | Hedonist Milieu | -0.15 | 0.07 | 0.567 | -0.46 | 0.16 |
|  | Traditional Milieu (*N* = 98) | Established Milieu | 0.11 | 0.10 | 0.987 | -0.32 | 0.53 |
|  |  | Liberal Intellectual Milieu | 0.26 | 0.10 | 0.183 | -0.15 | 0.68 |
|  |  | Performer Milieu | 0.02 | 0.12 | 1.000 | -0.47 | 0.52 |
|  |  | Cosmopolitan Avant-garde Milieu | 0.01 | 0.11 | 1.000 | -0.45 | 0.48 |
|  |  | Adaptive Navigator Milieu | -0.05 | 0.10 | 1.000 | -0.46 | 0.35 |
|  |  | Social Ecological Milieu | 0.13 | 0.09 | 0.870 | -0.23 | 0.50 |
|  |  | Modern Mainstreamer Milieu | -0.06 | 0.10 | 1.000 | -0.46 | 0.34 |
|  |  | Precarious Milieu | -0.25 | 0.09 | 0.154 | -0.65 | 0.14 |
|  |  | Hedonist Milieu | -0.21 | 0.09 | 0.405 | -0.60 | 0.18 |
|  | Precarious Milieu (*N* = 229) | Established Milieu | 0.36* | 0.08 | **< 0.001** | 0.02 | 0.71 |
|  |  | Liberal Intellectual Milieu | 0.52* | 0.08 | **< 0.001** | 0.18 | 0.86 |
|  |  | Performer Milieu | 0.28 | 0.10 | 0.153 | -0.15 | 0.71 |
|  |  | Cosmopolitan Avant-garde Milieu | 0.27 | 0.09 | 0.118 | -0.13 | 0.66 |
|  |  | Adaptive Navigator Milieu | 0.20 | 0.08 | 0.202 | -0.12 | 0.52 |
|  |  | Social Ecological Milieu | 0.39* | 0.06 | **< 0.001** | 0.12 | 0.66 |
|  |  | Modern Mainstreamer Milieu | 0.20 | 0.07 | 0.203 | -0.12 | 0.51 |
|  |  | Traditional Milieu | 0.25 | 0.09 | 0.154 | -0.14 | 0.65 |
|  |  | Hedonist Milieu | 0.05 | 0.07 | 1.000 | -0.25 | 0.35 |
|  | Hedonist Milieu (*N* = 241) | Established Milieu | 0.32 | 0.08 | 0.004 | -0.03 | 0.66 |
|  |  | Liberal Intellectual Milieu | 0.47* | 0.08 | **< 0.001** | 0.14 | 0.81 |
|  |  | Performer Milieu | 0.23 | 0.10 | 0.377 | -0.19 | 0.66 |
|  |  | Cosmopolitan Avant-garde Milieu | 0.22 | 0.09 | 0.335 | -0.17 | 0.61 |
|  |  | Adaptive Navigator Milieu | 0.16 | 0.08 | 0.557 | -0.16 | 0.47 |
|  |  | Social Ecological Milieu | 0.34* | 0.06 | **< 0.001** | 0.08 | 0.61 |
|  |  | Modern Mainstreamer Milieu | 0.15 | 0.07 | 0.567 | -0.16 | 0.46 |
|  |  | Traditional Milieu | 0.21 | 0.09 | 0.405 | -0.18 | 0.60 |
|  |  | Precarious Milieu | -0.05 | 0.07 | 1.000 | -0.35 | 0.25 |
| **HEALTH-49 (*N* = 1,829) – (10) Social stress** | | | | | | | |
| **Tukey post-hoc test** | | | | | | | |
|  | Established Milieu (*N* = 144) | Liberal Intellectual Milieu | 0.15 | 0.08 | 0.673 | -0.19 | 0.50 |
|  |  | Performer Milieu | -0.15 | 0.10 | 0.896 | -0.57 | 0.27 |
|  |  | Cosmopolitan Avant-garde Milieu | -0.08 | 0.09 | 0.996 | -0.48 | 0.31 |
|  |  | Adaptive Navigator Milieu | -0.26 | 0.08 | 0.033 | -0.59 | 0.07 |
|  |  | Social Ecological Milieu | -0.08 | 0.07 | 0.975 | -0.37 | 0.21 |
|  |  | Modern Mainstreamer Milieu | -0.25 | 0.08 | 0.041 | -0.58 | 0.08 |
|  |  | Traditional Milieu | -0.25 | 0.09 | 0.161 | -0.64 | 0.14 |
|  |  | Precarious Milieu | -0.44* | 0.08 | **< 0.001** | -0.76 | -0.13 |
|  |  | Hedonist Milieu | -0.34* | 0.07 | **< 0.001** | -0.66 | -0.03 |
|  | Liberal Intellectual Milieu (*N* = 157) | Established Milieu | -0.15 | 0.08 | 0.673 | -0.50 | 0.19 |
|  |  | Performer Milieu | -0.30 | 0.10 | 0.065 | -0.72 | 0.11 |
|  |  | Cosmopolitan Avant-garde Milieu | -0.24 | 0.09 | 0.211 | -0.62 | 0.15 |
|  |  | Adaptive Navigator Milieu | -0.41* | 0.08 | **< 0.001** | -0.74 | -0.09 |
|  |  | Social Ecological Milieu | -0.24 | 0.07 | 0.014 | -0.52 | 0.05 |
|  |  | Modern Mainstreamer Milieu | -0.41* | 0.08 | **< 0.001** | -0.73 | -0.08 |
|  |  | Traditional Milieu | -0.41* | 0.09 | **< 0.001** | -0.79 | -0.02 |
|  |  | Precarious Milieu | -0.60* | 0.07 | **< 0.001** | -0.91 | -0.29 |
|  |  | Hedonist Milieu | -0.50* | 0.07 | **< 0.001** | -0.80 | -0.19 |
|  | Performer Milieu (*N* = 76) | Established Milieu | 0.15 | 0.10 | 0.896 | -0.27 | 0.57 |
|  |  | Liberal Intellectual Milieu | 0.30 | 0.10 | 0.065 | -0.11 | 0.72 |
|  |  | Cosmopolitan Avant-garde Milieu | 0.07 | 0.11 | 1.000 | -0.39 | 0.52 |
|  |  | Adaptive Navigator Milieu | -0.11 | 0.10 | 0.980 | -0.52 | 0.30 |
|  |  | Social Ecological Milieu | 0.07 | 0.09 | 0.999 | -0.31 | 0.44 |
|  |  | Modern Mainstreamer Milieu | -0.10 | 0.10 | 0.988 | -0.51 | 0.30 |
|  |  | Traditional Milieu | -0.10 | 0.11 | 0.994 | -0.56 | 0.35 |
|  |  | Precarious Milieu | -0.29 | 0.09 | 0.053 | -0.69 | 0.10 |
|  |  | Hedonist Milieu | -0.19 | 0.09 | 0.550 | -0.59 | 0.20 |
|  | Cosmopolitan Avant-garde Milieu (*N* = 97) | Established Milieu | 0.08 | 0.09 | 0.996 | -0.31 | 0.48 |
|  |  | Liberal Intellectual Milieu | 0.24 | 0.09 | 0.211 | -0.15 | 0.62 |
|  |  | Performer Milieu | -0.07 | 0.11 | 1.000 | -0.52 | 0.39 |
|  |  | Adaptive Navigator Milieu | -0.18 | 0.09 | 0.613 | -0.55 | 0.20 |
|  |  | Social Ecological Milieu | 0.00 | 0.08 | 1.000 | -0.33 | 0.34 |
|  |  | Modern Mainstreamer Milieu | -0.17 | 0.09 | 0.668 | -0.54 | 0.20 |
|  |  | Traditional Milieu | -0.17 | 0.10 | 0.813 | -0.60 | 0.26 |
|  |  | Precarious Milieu | -0.36 | 0.09 | **0.001** | -0.72 | 0.00 |
|  |  | Hedonist Milieu | -0.26 | 0.08 | 0.074 | -0.62 | 0.10 |
|  | Adaptive Navigator Milieu (*N* = 182) | Established Milieu | 0.26 | 0.08 | 0.033 | -0.07 | 0.59 |
|  |  | Liberal Intellectual Milieu | 0.41* | 0.08 | **< 0.001** | 0.09 | 0.74 |
|  |  | Performer Milieu | 0.11 | 0.10 | 0.980 | -0.30 | 0.52 |
|  |  | Cosmopolitan Avant-garde Milieu | 0.18 | 0.09 | 0.613 | -0.20 | 0.55 |
|  |  | Social Ecological Milieu | 0.18 | 0.06 | 0.122 | -0.09 | 0.45 |
|  |  | Modern Mainstreamer Milieu | 0.01 | 0.07 | 1.000 | -0.30 | 0.32 |
|  |  | Traditional Milieu | 0.01 | 0.09 | 1.000 | -0.37 | 0.38 |
|  |  | Precarious Milieu | -0.18 | 0.07 | 0.211 | -0.48 | 0.11 |
|  |  | Hedonist Milieu | -0.08 | 0.07 | 0.976 | -0.38 | 0.21 |
|  | Social Ecological Milieu (*N* = 410) | Established Milieu | 0.08 | 0.07 | 0.975 | -0.21 | 0.37 |
|  |  | Liberal Intellectual Milieu | 0.24 | 0.07 | 0.014 | -0.05 | 0.52 |
|  |  | Performer Milieu | -0.07 | 0.09 | 0.999 | -0.44 | 0.31 |
|  |  | Cosmopolitan Avant-garde Milieu | 0.00 | 0.08 | 1.000 | -0.34 | 0.33 |
|  |  | Adaptive Navigator Milieu | -0.18 | 0.06 | 0.122 | -0.45 | 0.09 |
|  |  | Modern Mainstreamer Milieu | -0.17 | 0.06 | 0.148 | -0.43 | 0.09 |
|  |  | Traditional Milieu | -0.17 | 0.08 | 0.482 | -0.51 | 0.16 |
|  |  | Precarious Milieu | -0.36* | 0.06 | **< 0.001** | -0.61 | -0.12 |
|  |  | Hedonist Milieu | -0.26* | 0.06 | **< 0.001** | -0.50 | -0.02 |
|  | Modern Mainstreamer Milieu (*N* = 195) | Established Milieu | 0.25 | 0.08 | 0.041 | -0.08 | 0.58 |
|  |  | Liberal Intellectual Milieu | 0.41* | 0.08 | **< 0.001** | 0.08 | 0.73 |
|  |  | Performer Milieu | 0.10 | 0.10 | 0.988 | -0.30 | 0.51 |
|  |  | Cosmopolitan Avant-garde Milieu | 0.17 | 0.09 | 0.668 | -0.20 | 0.54 |
|  |  | Adaptive Navigator Milieu | -0.01 | 0.07 | 1.000 | -0.32 | 0.30 |
|  |  | Social Ecological Milieu | 0.17 | 0.06 | 0.148 | -0.09 | 0.43 |
|  |  | Traditional Milieu | 0.00 | 0.09 | 1.000 | -0.37 | 0.37 |
|  |  | Precarious Milieu | -0.19 | 0.07 | 0.138 | -0.48 | 0.10 |
|  |  | Hedonist Milieu | -0.09 | 0.07 | 0.947 | -0.38 | 0.20 |
|  | Traditional Milieu (*N* = 98) | Established Milieu | 0.25 | 0.09 | 0.161 | -0.14 | 0.64 |
|  |  | Liberal Intellectual Milieu | 0.41* | 0.09 | **< 0.001** | 0.02 | 0.79 |
|  |  | Performer Milieu | 0.10 | 0.11 | 0.994 | -0.35 | 0.56 |
|  |  | Cosmopolitan Avant-garde Milieu | 0.17 | 0.10 | 0.813 | -0.26 | 0.60 |
|  |  | Adaptive Navigator Milieu | -0.01 | 0.09 | 1.000 | -0.38 | 0.37 |
|  |  | Social Ecological Milieu | 0.17 | 0.08 | 0.482 | -0.16 | 0.51 |
|  |  | Modern Mainstreamer Milieu | 0.00 | 0.09 | 1.000 | -0.37 | 0.37 |
|  |  | Precarious Milieu | -0.19 | 0.09 | 0.433 | -0.55 | 0.17 |
|  |  | Hedonist Milieu | -0.09 | 0.08 | 0.989 | -0.45 | 0.27 |
|  | Precarious Milieu (*N* = 229) | Established Milieu | 0.44* | 0.08 | **< 0.001** | 0.13 | 0.76 |
|  |  | Liberal Intellectual Milieu | 0.60* | 0.07 | **< 0.001** | 0.29 | 0.91 |
|  |  | Performer Milieu | 0.29 | 0.09 | 0.053 | -0.10 | 0.69 |
|  |  | Cosmopolitan Avant-garde Milieu | 0.36 | 0.09 | 0.001 | 0.00 | 0.72 |
|  |  | Adaptive Navigator Milieu | 0.18 | 0.07 | 0.211 | -0.11 | 0.48 |
|  |  | Social Ecological Milieu | 0.36* | 0.06 | **< 0.001** | 0.12 | 0.61 |
|  |  | Modern Mainstreamer Milieu | 0.19 | 0.07 | 0.138 | -0.10 | 0.48 |
|  |  | Traditional Milieu | 0.19 | 0.09 | 0.433 | -0.17 | 0.55 |
|  |  | Hedonist Milieu | 0.10 | 0.07 | 0.866 | -0.17 | 0.38 |
|  | Hedonist Milieu (*N* = 241) | Established Milieu | 0.34* | 0.07 | **< 0.001** | 0.03 | 0.66 |
|  |  | Liberal Intellectual Milieu | 0.50* | 0.07 | **< 0.001** | 0.19 | 0.80 |
|  |  | Performer Milieu | 0.19 | 0.09 | 0.550 | -0.20 | 0.59 |
|  |  | Cosmopolitan Avant-garde Milieu | 0.26 | 0.08 | 0.074 | -0.10 | 0.62 |
|  |  | Adaptive Navigator Milieu | 0.08 | 0.07 | 0.976 | -0.21 | 0.38 |
|  |  | Social Ecological Milieu | 0.26* | 0.06 | **< 0.001** | 0.02 | 0.50 |
|  |  | Modern Mainstreamer Milieu | 0.09 | 0.07 | 0.947 | -0.20 | 0.38 |
|  |  | Traditional Milieu | 0.09 | 0.08 | 0.989 | -0.27 | 0.45 |
|  |  | Precarious Milieu | -0.10 | 0.07 | 0.866 | -0.38 | 0.17 |

MD, mean difference; SE, standard error; *p*, value of *p*; CI, confidence interval; LL, lower limit; UL, upper limit.

Comparisons were based on the observed means and were performed for the average of the two levels of the within-subject factor (time). The Bonferroni adjusted significance level is α_adj_ ≤ 0.001, significant mean differences are marked with an asterisk (*).
