# Supplementary material for: Milieu-specific differences in symptom severity and treatment outcome in psychosomatic rehabilitation in Germany
Source: Front Psychiatry. 2023 Aug 15;14:1198146. doi: 10.3389/fpsyt.2023.1198146 (PMC10465793; doi:10.3389/fpsyt.2023.1198146)
Supplement: Supplementary file 2 [file Table_2.docx]

Supplementary Material

Milieu-specific differences in symptom severity and treatment outcome in psychosomatic rehabilitation in Germany

Henrika Kleineberg-Massuthe^*^, Lilia Papst, Markus Bassler, Volker Köllner

*** Correspondence:** Henrika Kleineberg-Massuthe: henrika.kleineberg-massuthe@charite.de

**Supplementary Table 2.** Group differences in symptom and impairment improvement in Tukey and Games-Howell post-hoc tests for the BDI-II and HEALTH-49 Psychological well-being, Interactional difficulties and Activity and Participation.

|  |  |  |  |  |  | **99.9% CI** | |
| --- | --- | --- | --- | --- | --- | --- | --- |
|  | **Milieu (A)** | **Milieu (B)** | **MD (A-B)** | **SE** | ***p*** | **LL** | **UL** |
| **BDI-II (*N* = 1,832)** | | | | | | | |
| **Games-Howell post-hoc test** | | | | | | | |
|  | Established Milieu (*N* = 144) | Liberal Intellectual Milieu | 0.20 | 1.03 | 1.000 | -4.22 | 4.61 |
|  |  | Performer Milieu | -0.51 | 1.12 | 1.000 | -5.36 | 4.35 |
|  |  | Cosmopolitan Avant-garde Milieu | -2.52 | 1.21 | 0.540 | -7.75 | 2.71 |
|  |  | Adaptive Navigator Milieu | -1.43 | 1.03 | 0.932 | -5.86 | 3.00 |
|  |  | Social Ecological Milieu | 0.48 | 0.83 | 1.000 | -3.10 | 4.05 |
|  |  | Modern Mainstreamer Milieu | -2.12 | 0.99 | 0.504 | -6.38 | 2.13 |
|  |  | Traditional Milieu | 1.22 | 1.29 | 0.995 | -4.37 | 6.82 |
|  |  | Precarious Milieu | -1.46 | 0.96 | 0.884 | -5.57 | 2.65 |
|  |  | Hedonist Milieu | 0.12 | 0.95 | 1.000 | -3.96 | 4.20 |
|  | Liberal Intellectual Milieu (*N* = 157) | Established Milieu | -0.20 | 1.03 | 1.000 | -4.61 | 4.22 |
|  |  | Performer Milieu | -0.70 | 1.17 | 1.000 | -5.77 | 4.36 |
|  |  | Cosmopolitan Avant-garde Milieu | -2.71 | 1.26 | 0.487 | -8.14 | 2.71 |
|  |  | Adaptive Navigator Milieu | -1.63 | 1.09 | 0.895 | -6.30 | 3.04 |
|  |  | Social Ecological Milieu | 0.28 | 0.90 | 1.000 | -3.59 | 4.16 |
|  |  | Modern Mainstreamer Milieu | -2.32 | 1.05 | 0.457 | -6.83 | 2.19 |
|  |  | Traditional Milieu | 1.03 | 1.34 | 0.999 | -4.75 | 6.81 |
|  |  | Precarious Milieu | -1.66 | 1.02 | 0.837 | -6.03 | 2.71 |
|  |  | Hedonist Milieu | -0.08 | 1.01 | 1.000 | -4.42 | 4.26 |
|  | Performer Milieu (*N* = 76) | Established Milieu | 0.51 | 1.12 | 1.000 | -4.35 | 5.36 |
|  |  | Liberal Intellectual Milieu | 0.70 | 1.17 | 1.000 | -4.36 | 5.77 |
|  |  | Cosmopolitan Avant-garde Milieu | -2.01 | 1.33 | 0.885 | -7.78 | 3.75 |
|  |  | Adaptive Navigator Milieu | -0.92 | 1.17 | 0.999 | -6.00 | 4.15 |
|  |  | Social Ecological Milieu | 0.98 | 1.00 | 0.993 | -3.41 | 5.37 |
|  |  | Modern Mainstreamer Milieu | -1.62 | 1.14 | 0.919 | -6.55 | 3.32 |
|  |  | Traditional Milieu | 1.73 | 1.40 | 0.966 | -4.36 | 7.82 |
|  |  | Precarious Milieu | -0.95 | 1.11 | 0.997 | -5.77 | 3.86 |
|  |  | Hedonist Milieu | 0.62 | 1.10 | 1.000 | -4.16 | 5.41 |
|  | Cosmopolitan Avant-garde Milieu (*N* = 97) | Established Milieu | 2.52 | 1.21 | 0.540 | -2.71 | 7.75 |
|  |  | Liberal Intellectual Milieu | 2.71 | 1.26 | 0.487 | -2.71 | 8.14 |
|  |  | Performer Milieu | 2.01 | 1.33 | 0.885 | -3.75 | 7.78 |
|  |  | Adaptive Navigator Milieu | 1.09 | 1.26 | 0.997 | -4.35 | 6.53 |
|  |  | Social Ecological Milieu | 3.00 | 1.10 | 0.176 | -1.81 | 7.80 |
|  |  | Modern Mainstreamer Milieu | 0.39 | 1.23 | 1.000 | -4.91 | 5.70 |
|  |  | Traditional Milieu | 3.74 | 1.48 | 0.258 | -2.65 | 10.13 |
|  |  | Precarious Milieu | 1.06 | 1.20 | 0.997 | -4.14 | 6.25 |
|  |  | Hedonist Milieu | 2.64 | 1.19 | 0.455 | -2.53 | 7.81 |
|  | Adaptive Navigator Milieu (*N* = 182) | Established Milieu | 1.43 | 1.03 | 0.932 | -3.00 | 5.86 |
|  |  | Liberal Intellectual Milieu | 1.63 | 1.09 | 0.895 | -3.04 | 6.30 |
|  |  | Performer Milieu | 0.92 | 1.17 | 0.999 | -4.15 | 6.00 |
|  |  | Cosmopolitan Avant-garde Milieu | -1.09 | 1.26 | 0.997 | -6.53 | 4.35 |
|  |  | Social Ecological Milieu | 1.91 | 0.91 | 0.527 | -1.98 | 5.80 |
|  |  | Modern Mainstreamer Milieu | -0.69 | 1.06 | 1.000 | -5.21 | 3.83 |
|  |  | Traditional Milieu | 2.65 | 1.34 | 0.613 | -3.14 | 8.45 |
|  |  | Precarious Milieu | -0.03 | 1.03 | 1.000 | -4.41 | 4.35 |
|  |  | Hedonist Milieu | 1.55 | 1.02 | 0.883 | -2.80 | 5.90 |
|  | Social Ecological Milieu (*N* = 412) | Established Milieu | -0.48 | 0.83 | 1.000 | -4.05 | 3.10 |
|  |  | Liberal Intellectual Milieu | -0.28 | 0.90 | 1.000 | -4.16 | 3.59 |
|  |  | Performer Milieu | -0.98 | 1.00 | 0.993 | -5.37 | 3.41 |
|  |  | Cosmopolitan Avant-garde Milieu | -3.00 | 1.10 | 0.176 | -7.80 | 1.81 |
|  |  | Adaptive Navigator Milieu | -1.91 | 0.91 | 0.527 | -5.80 | 1.98 |
|  |  | Modern Mainstreamer Milieu | -2.60 | 0.86 | 0.080 | -6.29 | 1.09 |
|  |  | Traditional Milieu | 0.75 | 1.19 | 1.000 | -4.46 | 5.95 |
|  |  | Precarious Milieu | -1.94 | 0.82 | 0.356 | -5.45 | 1.57 |
|  |  | Hedonist Milieu | -0.36 | 0.81 | 1.000 | -3.83 | 3.11 |
|  | Modern Mainstreamer Milieu (*N* = 196) | Established Milieu | 2.12 | 0.99 | 0.504 | -2.13 | 6.38 |
|  |  | Liberal Intellectual Milieu | 2.32 | 1.05 | 0.457 | -2.19 | 6.83 |
|  |  | Performer Milieu | 1.62 | 1.14 | 0.919 | -3.32 | 6.55 |
|  |  | Cosmopolitan Avant-garde Milieu | -0.39 | 1.23 | 1.000 | -5.70 | 4.91 |
|  |  | Adaptive Navigator Milieu | 0.69 | 1.06 | 1.000 | -3.83 | 5.21 |
|  |  | Social Ecological Milieu | 2.60 | 0.86 | 0.080 | -1.09 | 6.29 |
|  |  | Traditional Milieu | 3.35 | 1.31 | 0.246 | -2.32 | 9.02 |
|  |  | Precarious Milieu | 0.66 | 0.99 | 1.000 | -3.54 | 4.87 |
|  |  | Hedonist Milieu | 2.24 | 0.98 | 0.397 | -1.94 | 6.42 |
|  | Traditional Milieu (*N* = 98) | Established Milieu | -1.22 | 1.29 | 0.995 | -6.82 | 4.37 |
|  |  | Liberal Intellectual Milieu | -1.03 | 1.34 | 0.999 | -6.81 | 4.75 |
|  |  | Performer Milieu | -1.73 | 1.40 | 0.966 | -7.82 | 4.36 |
|  |  | Cosmopolitan Avant-garde Milieu | -3.74 | 1.48 | 0.258 | -10.13 | 2.65 |
|  |  | Adaptive Navigator Milieu | -2.65 | 1.34 | 0.613 | -8.45 | 3.14 |
|  |  | Social Ecological Milieu | -0.75 | 1.19 | 1.000 | -5.95 | 4.46 |
|  |  | Modern Mainstreamer Milieu | -3.35 | 1.31 | 0.246 | -9.02 | 2.32 |
|  |  | Precarious Milieu | -2.68 | 1.28 | 0.537 | -8.25 | 2.88 |
|  |  | Hedonist Milieu | -1.10 | 1.28 | 0.997 | -6.65 | 4.44 |
|  | Precarious Milieu (*N* = 229) | Established Milieu | 1.46 | 0.96 | 0.884 | -2.65 | 5.57 |
|  |  | Liberal Intellectual Milieu | 1.66 | 1.02 | 0.837 | -2.71 | 6.03 |
|  |  | Performer Milieu | 0.95 | 1.11 | 0.997 | -3.86 | 5.77 |
|  |  | Cosmopolitan Avant-garde Milieu | -1.06 | 1.20 | 0.997 | -6.25 | 4.14 |
|  |  | Adaptive Navigator Milieu | 0.03 | 1.03 | 1.000 | -4.35 | 4.41 |
|  |  | Social Ecological Milieu | 1.94 | 0.82 | 0.356 | -1.57 | 5.45 |
|  |  | Modern Mainstreamer Milieu | -0.66 | 0.99 | 1.000 | -4.87 | 3.54 |
|  |  | Traditional Milieu | 2.68 | 1.28 | 0.537 | -2.88 | 8.25 |
|  |  | Hedonist Milieu | 1.58 | 0.94 | 0.810 | -2.45 | 5.60 |
|  | Hedonist Milieu (*N* = 241) | Established Milieu | -0.12 | 0.95 | 1.000 | -4.20 | 3.96 |
|  |  | Liberal Intellectual Milieu | 0.08 | 1.01 | 1.000 | -4.26 | 4.42 |
|  |  | Performer Milieu | -0.62 | 1.10 | 1.000 | -5.41 | 4.16 |
|  |  | Cosmopolitan Avant-garde Milieu | -2.64 | 1.19 | 0.455 | -7.81 | 2.53 |
|  |  | Adaptive Navigator Milieu | -1.55 | 1.02 | 0.883 | -5.90 | 2.80 |
|  |  | Social Ecological Milieu | 0.36 | 0.81 | 1.000 | -3.11 | 3.83 |
|  |  | Modern Mainstreamer Milieu | -2.24 | 0.98 | 0.397 | -6.42 | 1.94 |
|  |  | Traditional Milieu | 1.10 | 1.28 | 0.997 | -4.44 | 6.65 |
|  |  | Precarious Milieu | -1.58 | 0.94 | 0.810 | -5.60 | 2.45 |
| **HEALTH-49 (*N* = 1,829) – (5) Psychological well-being** | | | | | | | |
| **Tukey post-hoc test** | | | | | | | |
|  | Established Milieu (*N* = 144) | Liberal Intellectual Milieu | 0.07 | 0.09 | 0.999 | -0.32 | 0.46 |
|  |  | Performer Milieu | 0.05 | 0.11 | 1.000 | -0.43 | 0.53 |
|  |  | Cosmopolitan Avant-garde Milieu | -0.07 | 0.11 | 1.000 | -0.52 | 0.37 |
|  |  | Adaptive Navigator Milieu | -0.17 | 0.09 | 0.692 | -0.54 | 0.21 |
|  |  | Social Ecological Milieu | 0.00 | 0.08 | 1.000 | -0.33 | 0.33 |
|  |  | Modern Mainstreamer Milieu | -0.15 | 0.09 | 0.772 | -0.53 | 0.22 |
|  |  | Traditional Milieu | -0.14 | 0.10 | 0.944 | -0.58 | 0.30 |
|  |  | Precarious Milieu | -0.27 | 0.09 | 0.058 | -0.63 | 0.09 |
|  |  | Hedonist Milieu | -0.11 | 0.08 | 0.961 | -0.46 | 0.25 |
|  | Liberal Intellectual Milieu (*N* = 157) | Established Milieu | -0.07 | 0.09 | 0.999 | -0.46 | 0.32 |
|  |  | Performer Milieu | -0.01 | 0.11 | 1.000 | -0.49 | 0.46 |
|  |  | Cosmopolitan Avant-garde Milieu | -0.14 | 0.10 | 0.946 | -0.58 | 0.30 |
|  |  | Adaptive Navigator Milieu | -0.23 | 0.09 | 0.185 | -0.60 | 0.14 |
|  |  | Social Ecological Milieu | -0.07 | 0.08 | 0.997 | -0.39 | 0.25 |
|  |  | Modern Mainstreamer Milieu | -0.22 | 0.09 | 0.237 | -0.58 | 0.14 |
|  |  | Traditional Milieu | -0.21 | 0.10 | 0.592 | -0.64 | 0.23 |
|  |  | Precarious Milieu | -0.33 | 0.08 | 0.003 | -0.68 | 0.02 |
|  |  | Hedonist Milieu | -0.17 | 0.08 | 0.521 | -0.52 | 0.17 |
|  | Performer Milieu (*N* = 76) | Established Milieu | -0.05 | 0.11 | 1.000 | -0.53 | 0.43 |
|  |  | Liberal Intellectual Milieu | 0.01 | 0.11 | 1.000 | -0.46 | 0.49 |
|  |  | Cosmopolitan Avant-garde Milieu | -0.12 | 0.12 | 0.992 | -0.64 | 0.40 |
|  |  | Adaptive Navigator Milieu | -0.22 | 0.11 | 0.600 | -0.68 | 0.24 |
|  |  | Social Ecological Milieu | -0.05 | 0.10 | 1.000 | -0.48 | 0.37 |
|  |  | Modern Mainstreamer Milieu | -0.21 | 0.11 | 0.673 | -0.66 | 0.25 |
|  |  | Traditional Milieu | -0.19 | 0.12 | 0.861 | -0.71 | 0.33 |
|  |  | Precarious Milieu | -0.32 | 0.11 | 0.082 | -0.77 | 0.13 |
|  |  | Hedonist Milieu | -0.16 | 0.11 | 0.890 | -0.60 | 0.29 |
|  | Cosmopolitan Avant-garde Milieu (*N* = 97) | Established Milieu | 0.07 | 0.11 | 1.000 | -0.37 | 0.52 |
|  |  | Liberal Intellectual Milieu | 0.14 | 0.10 | 0.946 | -0.30 | 0.58 |
|  |  | Performer Milieu | 0.12 | 0.12 | 0.992 | -0.40 | 0.64 |
|  |  | Adaptive Navigator Milieu | -0.10 | 0.10 | 0.995 | -0.52 | 0.33 |
|  |  | Social Ecological Milieu | 0.07 | 0.09 | 0.999 | -0.31 | 0.45 |
|  |  | Modern Mainstreamer Milieu | -0.08 | 0.10 | 0.998 | -0.50 | 0.34 |
|  |  | Traditional Milieu | -0.07 | 0.11 | 1.000 | -0.56 | 0.42 |
|  |  | Precarious Milieu | -0.19 | 0.10 | 0.594 | -0.61 | 0.22 |
|  |  | Hedonist Milieu | -0.04 | 0.10 | 1.000 | -0.44 | 0.37 |
|  | Adaptive Navigator Milieu (*N* = 182) | Established Milieu | 0.17 | 0.09 | 0.692 | -0.21 | 0.54 |
|  |  | Liberal Intellectual Milieu | 0.23 | 0.09 | 0.185 | -0.14 | 0.60 |
|  |  | Performer Milieu | 0.22 | 0.11 | 0.600 | -0.24 | 0.68 |
|  |  | Cosmopolitan Avant-garde Milieu | 0.10 | 0.10 | 0.995 | -0.33 | 0.52 |
|  |  | Social Ecological Milieu | 0.17 | 0.07 | 0.372 | -0.14 | 0.47 |
|  |  | Modern Mainstreamer Milieu | 0.01 | 0.08 | 1.000 | -0.34 | 0.36 |
|  |  | Traditional Milieu | 0.03 | 0.10 | 1.000 | -0.40 | 0.45 |
|  |  | Precarious Milieu | -0.10 | 0.08 | 0.965 | -0.44 | 0.24 |
|  |  | Hedonist Milieu | 0.06 | 0.08 | 0.999 | -0.27 | 0.39 |
|  | Social Ecological Milieu (*N* = 410) | Established Milieu | 0.00 | 0.08 | 1.000 | -0.33 | 0.33 |
|  |  | Liberal Intellectual Milieu | 0.07 | 0.08 | 0.997 | -0.25 | 0.39 |
|  |  | Performer Milieu | 0.05 | 0.10 | 1.000 | -0.37 | 0.48 |
|  |  | Cosmopolitan Avant-garde Milieu | -0.07 | 0.09 | 0.999 | -0.45 | 0.31 |
|  |  | Adaptive Navigator Milieu | -0.17 | 0.07 | 0.372 | -0.47 | 0.14 |
|  |  | Modern Mainstreamer Milieu | -0.15 | 0.07 | 0.464 | -0.45 | 0.14 |
|  |  | Traditional Milieu | -0.14 | 0.09 | 0.870 | -0.52 | 0.24 |
|  |  | Precarious Milieu | -0.26 | 0.07 | 0.003 | -0.54 | 0.01 |
|  |  | Hedonist Milieu | -0.11 | 0.06 | 0.833 | -0.38 | 0.17 |
|  | Modern Mainstreamer Milieu (*N* = 195) | Established Milieu | 0.15 | 0.09 | 0.772 | -0.22 | 0.53 |
|  |  | Liberal Intellectual Milieu | 0.22 | 0.09 | 0.237 | -0.14 | 0.58 |
|  |  | Performer Milieu | 0.21 | 0.11 | 0.673 | -0.25 | 0.66 |
|  |  | Cosmopolitan Avant-garde Milieu | 0.08 | 0.10 | 0.998 | -0.34 | 0.50 |
|  |  | Adaptive Navigator Milieu | -0.01 | 0.08 | 1.000 | -0.36 | 0.34 |
|  |  | Social Ecological Milieu | 0.15 | 0.07 | 0.464 | -0.14 | 0.45 |
|  |  | Traditional Milieu | 0.01 | 0.10 | 1.000 | -0.41 | 0.43 |
|  |  | Precarious Milieu | -0.11 | 0.08 | 0.914 | -0.44 | 0.22 |
|  |  | Hedonist Milieu | 0.05 | 0.08 | 1.000 | -0.28 | 0.37 |
|  | Traditional Milieu (*N* = 98) | Established Milieu | 0.14 | 0.10 | 0.944 | -0.30 | 0.58 |
|  |  | Liberal Intellectual Milieu | 0.21 | 0.10 | 0.592 | -0.23 | 0.64 |
|  |  | Performer Milieu | 0.19 | 0.12 | 0.861 | -0.33 | 0.71 |
|  |  | Cosmopolitan Avant-garde Milieu | 0.07 | 0.11 | 1.000 | -0.42 | 0.56 |
|  |  | Adaptive Navigator Milieu | -0.03 | 0.10 | 1.000 | -0.45 | 0.40 |
|  |  | Social Ecological Milieu | 0.14 | 0.09 | 0.870 | -0.24 | 0.52 |
|  |  | Modern Mainstreamer Milieu | -0.01 | 0.10 | 1.000 | -0.43 | 0.41 |
|  |  | Precarious Milieu | -0.13 | 0.10 | 0.955 | -0.53 | 0.28 |
|  |  | Hedonist Milieu | 0.03 | 0.10 | 1.000 | -0.37 | 0.44 |
|  | Precarious Milieu (*N* = 229) | Established Milieu | 0.27 | 0.09 | 0.058 | -0.09 | 0.63 |
|  |  | Liberal Intellectual Milieu | 0.33 | 0.08 | 0.003 | -0.02 | 0.68 |
|  |  | Performer Milieu | 0.32 | 0.11 | 0.082 | -0.13 | 0.77 |
|  |  | Cosmopolitan Avant-garde Milieu | 0.19 | 0.10 | 0.594 | -0.22 | 0.61 |
|  |  | Adaptive Navigator Milieu | 0.10 | 0.08 | 0.965 | -0.24 | 0.44 |
|  |  | Social Ecological Milieu | 0.26 | 0.07 | 0.003 | -0.01 | 0.54 |
|  |  | Modern Mainstreamer Milieu | 0.11 | 0.08 | 0.914 | -0.22 | 0.44 |
|  |  | Traditional Milieu | 0.13 | 0.10 | 0.955 | -0.28 | 0.53 |
|  |  | Hedonist Milieu | 0.16 | 0.07 | 0.490 | -0.15 | 0.47 |
|  | Hedonist Milieu (*N* = 241) | Established Milieu | 0.11 | 0.08 | 0.961 | -0.25 | 0.46 |
|  |  | Liberal Intellectual Milieu | 0.17 | 0.08 | 0.521 | -0.17 | 0.52 |
|  |  | Performer Milieu | 0.16 | 0.11 | 0.890 | -0.29 | 0.60 |
|  |  | Cosmopolitan Avant-garde Milieu | 0.04 | 0.10 | 1.000 | -0.37 | 0.44 |
|  |  | Adaptive Navigator Milieu | -0.06 | 0.08 | 0.999 | -0.39 | 0.27 |
|  |  | Social Ecological Milieu | 0.11 | 0.06 | 0.833 | -0.17 | 0.38 |
|  |  | Modern Mainstreamer Milieu | -0.05 | 0.08 | 1.000 | -0.37 | 0.28 |
|  |  | Traditional Milieu | -0.03 | 0.10 | 1.000 | -0.44 | 0.37 |
|  |  | Precarious Milieu | -0.16 | 0.07 | 0.490 | -0.47 | 0.15 |
| **HEALTH-49 (*N* = 1,829) – (6) Interactional difficulties** | | | | | | | |
| **Tukey post-hoc test** | | | | | | | |
|  | Established Milieu (*N* = 144) | Liberal Intellectual Milieu | 0.10 | 0.11 | 0.993 | -0.34 | 0.55 |
|  |  | Performer Milieu | -0.02 | 0.13 | 1.000 | -0.57 | 0.53 |
|  |  | Cosmopolitan Avant-garde Milieu | -0.01 | 0.12 | 1.000 | -0.52 | 0.50 |
|  |  | Adaptive Navigator Milieu | -0.13 | 0.10 | 0.960 | -0.56 | 0.30 |
|  |  | Social Ecological Milieu | 0.13 | 0.09 | 0.895 | -0.24 | 0.51 |
|  |  | Modern Mainstreamer Milieu | -0.12 | 0.10 | 0.979 | -0.54 | 0.31 |
|  |  | Traditional Milieu | -0.05 | 0.12 | 1.000 | -0.56 | 0.46 |
|  |  | Precarious Milieu | -0.15 | 0.10 | 0.868 | -0.56 | 0.26 |
|  |  | Hedonist Milieu | 0.05 | 0.10 | 1.000 | -0.36 | 0.46 |
|  | Liberal Intellectual Milieu (*N* = 157) | Established Milieu | -0.10 | 0.11 | 0.993 | -0.55 | 0.34 |
|  |  | Performer Milieu | -0.12 | 0.13 | 0.994 | -0.67 | 0.42 |
|  |  | Cosmopolitan Avant-garde Milieu | -0.11 | 0.12 | 0.995 | -0.61 | 0.39 |
|  |  | Adaptive Navigator Milieu | -0.23 | 0.10 | 0.360 | -0.66 | 0.19 |
|  |  | Social Ecological Milieu | 0.03 | 0.09 | 1.000 | -0.34 | 0.39 |
|  |  | Modern Mainstreamer Milieu | -0.22 | 0.10 | 0.429 | -0.64 | 0.20 |
|  |  | Traditional Milieu | -0.15 | 0.12 | 0.956 | -0.65 | 0.35 |
|  |  | Precarious Milieu | -0.26 | 0.10 | 0.176 | -0.66 | 0.15 |
|  |  | Hedonist Milieu | -0.06 | 0.09 | 1.000 | -0.45 | 0.34 |
|  | Performer Milieu (*N* = 76) | Established Milieu | 0.02 | 0.13 | 1.000 | -0.53 | 0.57 |
|  |  | Liberal Intellectual Milieu | 0.12 | 0.13 | 0.994 | -0.42 | 0.67 |
|  |  | Cosmopolitan Avant-garde Milieu | 0.01 | 0.14 | 1.000 | -0.58 | 0.61 |
|  |  | Adaptive Navigator Milieu | -0.11 | 0.13 | 0.997 | -0.64 | 0.42 |
|  |  | Social Ecological Milieu | 0.15 | 0.11 | 0.945 | -0.33 | 0.64 |
|  |  | Modern Mainstreamer Milieu | -0.10 | 0.12 | 0.999 | -0.62 | 0.43 |
|  |  | Traditional Milieu | -0.03 | 0.14 | 1.000 | -0.62 | 0.57 |
|  |  | Precarious Milieu | -0.13 | 0.12 | 0.986 | -0.65 | 0.38 |
|  |  | Hedonist Milieu | 0.07 | 0.12 | 1.000 | -0.44 | 0.58 |
|  | Cosmopolitan Avant-garde Milieu (*N* = 97) | Established Milieu | 0.01 | 0.12 | 1.000 | -0.50 | 0.52 |
|  |  | Liberal Intellectual Milieu | 0.11 | 0.12 | 0.995 | -0.39 | 0.61 |
|  |  | Performer Milieu | -0.01 | 0.14 | 1.000 | -0.61 | 0.58 |
|  |  | Adaptive Navigator Milieu | -0.12 | 0.12 | 0.987 | -0.61 | 0.36 |
|  |  | Social Ecological Milieu | 0.14 | 0.10 | 0.944 | -0.30 | 0.58 |
|  |  | Modern Mainstreamer Milieu | -0.11 | 0.11 | 0.994 | -0.59 | 0.37 |
|  |  | Traditional Milieu | -0.04 | 0.13 | 1.000 | -0.60 | 0.51 |
|  |  | Precarious Milieu | -0.15 | 0.11 | 0.951 | -0.62 | 0.32 |
|  |  | Hedonist Milieu | 0.05 | 0.11 | 1.000 | -0.41 | 0.52 |
|  | Adaptive Navigator Milieu (*N* = 182) | Established Milieu | 0.13 | 0.10 | 0.960 | -0.30 | 0.56 |
|  |  | Liberal Intellectual Milieu | 0.23 | 0.10 | 0.360 | -0.19 | 0.66 |
|  |  | Performer Milieu | 0.11 | 0.13 | 0.997 | -0.42 | 0.64 |
|  |  | Cosmopolitan Avant-garde Milieu | 0.12 | 0.12 | 0.987 | -0.36 | 0.61 |
|  |  | Social Ecological Milieu | 0.26 | 0.08 | 0.043 | -0.08 | 0.61 |
|  |  | Modern Mainstreamer Milieu | 0.01 | 0.09 | 1.000 | -0.39 | 0.41 |
|  |  | Traditional Milieu | 0.08 | 0.11 | 0.999 | -0.40 | 0.57 |
|  |  | Precarious Milieu | -0.02 | 0.09 | 1.000 | -0.41 | 0.36 |
|  |  | Hedonist Milieu | 0.18 | 0.09 | 0.617 | -0.20 | 0.56 |
|  | Social Ecological Milieu (*N* = 410) | Established Milieu | -0.13 | 0.09 | 0.895 | -0.51 | 0.24 |
|  |  | Liberal Intellectual Milieu | -0.03 | 0.09 | 1.000 | -0.39 | 0.34 |
|  |  | Performer Milieu | -0.15 | 0.11 | 0.945 | -0.64 | 0.33 |
|  |  | Cosmopolitan Avant-garde Milieu | -0.14 | 0.10 | 0.944 | -0.58 | 0.30 |
|  |  | Adaptive Navigator Milieu | -0.26 | 0.08 | 0.043 | -0.61 | 0.08 |
|  |  | Modern Mainstreamer Milieu | -0.25 | 0.08 | 0.058 | -0.59 | 0.09 |
|  |  | Traditional Milieu | -0.18 | 0.10 | 0.765 | -0.62 | 0.26 |
|  |  | Precarious Milieu | -0.28 | 0.08 | 0.007 | -0.60 | 0.04 |
|  |  | Hedonist Milieu | -0.08 | 0.07 | 0.981 | -0.40 | 0.23 |
|  | Modern Mainstreamer Milieu (*N* = 195) | Established Milieu | 0.12 | 0.10 | 0.979 | -0.31 | 0.54 |
|  |  | Liberal Intellectual Milieu | 0.22 | 0.10 | 0.429 | -0.20 | 0.64 |
|  |  | Performer Milieu | 0.10 | 0.12 | 0.999 | -0.43 | 0.62 |
|  |  | Cosmopolitan Avant-garde Milieu | 0.11 | 0.11 | 0.994 | -0.37 | 0.59 |
|  |  | Adaptive Navigator Milieu | -0.01 | 0.09 | 1.000 | -0.41 | 0.39 |
|  |  | Social Ecological Milieu | 0.25 | 0.08 | 0.058 | -0.09 | 0.59 |
|  |  | Traditional Milieu | 0.07 | 0.11 | 1.000 | -0.41 | 0.55 |
|  |  | Precarious Milieu | -0.04 | 0.09 | 1.000 | -0.41 | 0.34 |
|  |  | Hedonist Milieu | 0.16 | 0.09 | 0.699 | -0.21 | 0.54 |
|  | Traditional Milieu (*N* = 98) | Established Milieu | 0.05 | 0.12 | 1.000 | -0.46 | 0.56 |
|  |  | Liberal Intellectual Milieu | 0.15 | 0.12 | 0.956 | -0.35 | 0.65 |
|  |  | Performer Milieu | 0.03 | 0.14 | 1.000 | -0.57 | 0.62 |
|  |  | Cosmopolitan Avant-garde Milieu | 0.04 | 0.13 | 1.000 | -0.51 | 0.60 |
|  |  | Adaptive Navigator Milieu | -0.08 | 0.11 | 0.999 | -0.57 | 0.40 |
|  |  | Social Ecological Milieu | 0.18 | 0.10 | 0.765 | -0.26 | 0.62 |
|  |  | Modern Mainstreamer Milieu | -0.07 | 0.11 | 1.000 | -0.55 | 0.41 |
|  |  | Precarious Milieu | -0.10 | 0.11 | 0.995 | -0.57 | 0.36 |
|  |  | Hedonist Milieu | 0.10 | 0.11 | 0.997 | -0.37 | 0.56 |
|  | Precarious Milieu (*N* = 229) | Established Milieu | 0.15 | 0.10 | 0.868 | -0.26 | 0.56 |
|  |  | Liberal Intellectual Milieu | 0.26 | 0.10 | 0.176 | -0.15 | 0.66 |
|  |  | Performer Milieu | 0.13 | 0.12 | 0.986 | -0.38 | 0.65 |
|  |  | Cosmopolitan Avant-garde Milieu | 0.15 | 0.11 | 0.951 | -0.32 | 0.62 |
|  |  | Adaptive Navigator Milieu | 0.02 | 0.09 | 1.000 | -0.36 | 0.41 |
|  |  | Social Ecological Milieu | 0.28 | 0.08 | 0.007 | -0.04 | 0.60 |
|  |  | Modern Mainstreamer Milieu | 0.04 | 0.09 | 1.000 | -0.34 | 0.41 |
|  |  | Traditional Milieu | 0.10 | 0.11 | 0.995 | -0.36 | 0.57 |
|  |  | Hedonist Milieu | 0.20 | 0.08 | 0.351 | -0.16 | 0.56 |
|  | Hedonist Milieu (*N* = 241) | Established Milieu | -0.05 | 0.10 | 1.000 | -0.46 | 0.36 |
|  |  | Liberal Intellectual Milieu | 0.06 | 0.09 | 1.000 | -0.34 | 0.45 |
|  |  | Performer Milieu | -0.07 | 0.12 | 1.000 | -0.58 | 0.44 |
|  |  | Cosmopolitan Avant-garde Milieu | -0.05 | 0.11 | 1.000 | -0.52 | 0.41 |
|  |  | Adaptive Navigator Milieu | -0.18 | 0.09 | 0.617 | -0.56 | 0.20 |
|  |  | Social Ecological Milieu | 0.08 | 0.07 | 0.981 | -0.23 | 0.40 |
|  |  | Modern Mainstreamer Milieu | -0.16 | 0.09 | 0.699 | -0.54 | 0.21 |
|  |  | Traditional Milieu | -0.10 | 0.11 | 0.997 | -0.56 | 0.37 |
|  |  | Precarious Milieu | -0.20 | 0.08 | 0.351 | -0.56 | 0.16 |
| **HEALTH-49 (*N* = 1,829) – (8) Activity and Participation** | | | | | | | |
| **Tukey post-hoc test** | | | | | | | |
|  | Established Milieu (*N* = 144) | Liberal Intellectual Milieu | 0.09 | 0.10 | 0.996 | -0.34 | 0.52 |
|  |  | Performer Milieu | 0.08 | 0.12 | 1.000 | -0.44 | 0.61 |
|  |  | Cosmopolitan Avant-garde Milieu | 0.01 | 0.12 | 1.000 | -0.47 | 0.50 |
|  |  | Adaptive Navigator Milieu | -0.16 | 0.10 | 0.810 | -0.58 | 0.25 |
|  |  | Social Ecological Milieu | 0.09 | 0.09 | 0.991 | -0.27 | 0.45 |
|  |  | Modern Mainstreamer Milieu | -0.24 | 0.10 | 0.286 | -0.65 | 0.17 |
|  |  | Traditional Milieu | 0.09 | 0.11 | 0.999 | -0.40 | 0.58 |
|  |  | Precarious Milieu | -0.26 | 0.09 | 0.139 | -0.66 | 0.13 |
|  |  | Hedonist Milieu | -0.05 | 0.09 | 1.000 | -0.44 | 0.34 |
|  | Liberal Intellectual Milieu (*N* = 157) | Established Milieu | -0.09 | 0.10 | 0.996 | -0.52 | 0.34 |
|  |  | Performer Milieu | -0.01 | 0.12 | 1.000 | -0.53 | 0.51 |
|  |  | Cosmopolitan Avant-garde Milieu | -0.08 | 0.11 | 1.000 | -0.56 | 0.40 |
|  |  | Adaptive Navigator Milieu | -0.26 | 0.10 | 0.179 | -0.66 | 0.15 |
|  |  | Social Ecological Milieu | -0.01 | 0.08 | 1.000 | -0.36 | 0.34 |
|  |  | Modern Mainstreamer Milieu | -0.33 | 0.09 | 0.016 | -0.73 | 0.07 |
|  |  | Traditional Milieu | 0.00 | 0.11 | 1.000 | -0.48 | 0.47 |
|  |  | Precarious Milieu | -0.35 | 0.09 | 0.004 | -0.74 | 0.03 |
|  |  | Hedonist Milieu | -0.14 | 0.09 | 0.851 | -0.52 | 0.24 |
|  | Performer Milieu (*N* = 76) | Established Milieu | -0.08 | 0.12 | 1.000 | -0.61 | 0.44 |
|  |  | Liberal Intellectual Milieu | 0.01 | 0.12 | 1.000 | -0.51 | 0.53 |
|  |  | Cosmopolitan Avant-garde Milieu | -0.07 | 0.13 | 1.000 | -0.64 | 0.50 |
|  |  | Adaptive Navigator Milieu | -0.25 | 0.12 | 0.556 | -0.75 | 0.26 |
|  |  | Social Ecological Milieu | 0.00 | 0.11 | 1.000 | -0.46 | 0.47 |
|  |  | Modern Mainstreamer Milieu | -0.32 | 0.12 | 0.172 | -0.82 | 0.18 |
|  |  | Traditional Milieu | 0.01 | 0.13 | 1.000 | -0.56 | 0.57 |
|  |  | Precarious Milieu | -0.34 | 0.12 | 0.091 | -0.84 | 0.15 |
|  |  | Hedonist Milieu | -0.13 | 0.12 | 0.979 | -0.62 | 0.36 |
|  | Cosmopolitan Avant-garde Milieu (*N* = 97) | Established Milieu | -0.01 | 0.12 | 1.000 | -0.50 | 0.47 |
|  |  | Liberal Intellectual Milieu | 0.08 | 0.11 | 1.000 | -0.40 | 0.56 |
|  |  | Performer Milieu | 0.07 | 0.13 | 1.000 | -0.50 | 0.64 |
|  |  | Adaptive Navigator Milieu | -0.18 | 0.11 | 0.841 | -0.65 | 0.29 |
|  |  | Social Ecological Milieu | 0.07 | 0.10 | 0.999 | -0.35 | 0.49 |
|  |  | Modern Mainstreamer Milieu | -0.25 | 0.11 | 0.381 | -0.71 | 0.21 |
|  |  | Traditional Milieu | 0.08 | 0.13 | 1.000 | -0.46 | 0.61 |
|  |  | Precarious Milieu | -0.27 | 0.11 | 0.225 | -0.73 | 0.18 |
|  |  | Hedonist Milieu | -0.06 | 0.11 | 1.000 | -0.51 | 0.38 |
|  | Adaptive Navigator Milieu (*N* = 182) | Established Milieu | 0.16 | 0.10 | 0.810 | -0.25 | 0.58 |
|  |  | Liberal Intellectual Milieu | 0.26 | 0.10 | 0.179 | -0.15 | 0.66 |
|  |  | Performer Milieu | 0.25 | 0.12 | 0.556 | -0.26 | 0.75 |
|  |  | Cosmopolitan Avant-garde Milieu | 0.18 | 0.11 | 0.841 | -0.29 | 0.65 |
|  |  | Social Ecological Milieu | 0.25 | 0.08 | 0.045 | -0.08 | 0.58 |
|  |  | Modern Mainstreamer Milieu | -0.07 | 0.09 | 0.998 | -0.46 | 0.31 |
|  |  | Traditional Milieu | 0.25 | 0.11 | 0.385 | -0.21 | 0.72 |
|  |  | Precarious Milieu | -0.10 | 0.09 | 0.984 | -0.47 | 0.27 |
|  |  | Hedonist Milieu | 0.11 | 0.09 | 0.950 | -0.25 | 0.48 |
|  | Social Ecological Milieu (*N* = 410) | Established Milieu | -0.09 | 0.09 | 0.991 | -0.45 | 0.27 |
|  |  | Liberal Intellectual Milieu | 0.01 | 0.08 | 1.000 | -0.34 | 0.36 |
|  |  | Performer Milieu | 0.00 | 0.11 | 1.000 | -0.47 | 0.46 |
|  |  | Cosmopolitan Avant-garde Milieu | -0.07 | 0.10 | 0.999 | -0.49 | 0.35 |
|  |  | Adaptive Navigator Milieu | -0.25 | 0.08 | 0.045 | -0.58 | 0.08 |
|  |  | Modern Mainstreamer Milieu | -0.32* | 0.08 | **0.001** | -0.65 | 0.00 |
|  |  | Traditional Milieu | 0.00 | 0.10 | 1.000 | -0.41 | 0.42 |
|  |  | Precarious Milieu | -0.35* | 0.07 | **< 0.001** | -0.65 | -0.04 |
|  |  | Hedonist Milieu | -0.14 | 0.07 | 0.654 | -0.44 | 0.16 |
|  | Modern Mainstreamer Milieu (*N* = 195) | Established Milieu | 0.24 | 0.10 | 0.286 | -0.17 | 0.65 |
|  |  | Liberal Intellectual Milieu | 0.33 | 0.09 | 0.016 | -0.07 | 0.73 |
|  |  | Performer Milieu | 0.32 | 0.12 | 0.172 | -0.18 | 0.82 |
|  |  | Cosmopolitan Avant-garde Milieu | 0.25 | 0.11 | 0.381 | -0.21 | 0.71 |
|  |  | Adaptive Navigator Milieu | 0.07 | 0.09 | 0.998 | -0.31 | 0.46 |
|  |  | Social Ecological Milieu | 0.32* | 0.08 | **0.001** | 0.00 | 0.65 |
|  |  | Traditional Milieu | 0.33 | 0.11 | 0.078 | -0.13 | 0.79 |
|  |  | Precarious Milieu | -0.02 | 0.09 | 1.000 | -0.38 | 0.34 |
|  |  | Hedonist Milieu | 0.19 | 0.08 | 0.443 | -0.17 | 0.55 |
|  | Traditional Milieu (*N* = 98) | Established Milieu | -0.09 | 0.11 | 0.999 | -0.58 | 0.40 |
|  |  | Liberal Intellectual Milieu | 0.00 | 0.11 | 1.000 | -0.47 | 0.48 |
|  |  | Performer Milieu | -0.01 | 0.13 | 1.000 | -0.57 | 0.56 |
|  |  | Cosmopolitan Avant-garde Milieu | -0.08 | 0.13 | 1.000 | -0.61 | 0.46 |
|  |  | Adaptive Navigator Milieu | -0.25 | 0.11 | 0.385 | -0.72 | 0.21 |
|  |  | Social Ecological Milieu | 0.00 | 0.10 | 1.000 | -0.42 | 0.41 |
|  |  | Modern Mainstreamer Milieu | -0.33 | 0.11 | 0.078 | -0.79 | 0.13 |
|  |  | Precarious Milieu | -0.35 | 0.11 | 0.032 | -0.80 | 0.10 |
|  |  | Hedonist Milieu | -0.14 | 0.11 | 0.946 | -0.59 | 0.31 |
|  | Precarious Milieu (*N* = 229) | Established Milieu | 0.26 | 0.09 | 0.139 | -0.13 | 0.66 |
|  |  | Liberal Intellectual Milieu | 0.35 | 0.09 | 0.004 | -0.03 | 0.74 |
|  |  | Performer Milieu | 0.34 | 0.12 | 0.091 | -0.15 | 0.84 |
|  |  | Cosmopolitan Avant-garde Milieu | 0.27 | 0.11 | 0.225 | -0.18 | 0.73 |
|  |  | Adaptive Navigator Milieu | 0.10 | 0.09 | 0.984 | -0.27 | 0.47 |
|  |  | Social Ecological Milieu | 0.35* | 0.07 | **< 0.001** | 0.04 | 0.65 |
|  |  | Modern Mainstreamer Milieu | 0.02 | 0.09 | 1.000 | -0.34 | 0.38 |
|  |  | Traditional Milieu | 0.35 | 0.11 | 0.032 | -0.10 | 0.80 |
|  |  | Hedonist Milieu | 0.21 | 0.08 | 0.220 | -0.13 | 0.55 |
|  | Hedonist Milieu (*N* = 241) | Established Milieu | 0.05 | 0.09 | 1.000 | -0.34 | 0.44 |
|  |  | Liberal Intellectual Milieu | 0.14 | 0.09 | 0.851 | -0.24 | 0.52 |
|  |  | Performer Milieu | 0.13 | 0.12 | 0.979 | -0.36 | 0.62 |
|  |  | Cosmopolitan Avant-garde Milieu | 0.06 | 0.11 | 1.000 | -0.38 | 0.51 |
|  |  | Adaptive Navigator Milieu | -0.11 | 0.09 | 0.950 | -0.48 | 0.25 |
|  |  | Social Ecological Milieu | 0.14 | 0.07 | 0.654 | -0.16 | 0.44 |
|  |  | Modern Mainstreamer Milieu | -0.19 | 0.08 | 0.443 | -0.55 | 0.17 |
|  |  | Traditional Milieu | 0.14 | 0.11 | 0.946 | -0.31 | 0.59 |
|  |  | Precarious Milieu | -0.21 | 0.08 | 0.220 | -0.55 | 0.13 |

MD, mean difference; SE, standard error; *p*, value of *p*; CI, confidence interval; LL, lower limit; UL, upper limit.

Comparisons were based on the observed means of the pre-post-differences. The Bonferroni adjusted significance level is α_adj_ ≤ 0.001, significant mean differences are marked with an asterisk (*).
